# Dynamics of cognitive variability with age and its genetic underpinning in NIHR BioResource Genes and Cognition cohort participants

**DOI:** 10.1038/s41591-024-02960-5

**Published:** 2024-05-14

**Authors:** Md Shafiqur Rahman, Emma Harrison, Heather Biggs, Chloe Seikus, Paul Elliott, Gerome Breen, Nathalie Kingston, John R. Bradley, Steven M. Hill, Brian D. M. Tom, Patrick F. Chinnery

**Affiliations:** 1 MRC Biostatistics Unit, University of Cambridge, Cambridge, UK; 2 Department of Clinical Neurosciences, University of Cambridge, Cambridge, UK; 3 National Institute for Health and Care Research BioResource, Cambridge, UK; 4 Department of Epidemiology and Biostatistics, Imperial College London School of Public Health, London, UK; 5 Social, Genetic and Developmental Psychiatry Centre, Institute of Psychiatry, Psychology and Neuroscience, King’s College London, London, UK; 6 UK National Institute for Health Research Biomedical Research Centre for Mental Health, South London and Maudsley Hospital, London, UK; 7 Dept of Haematology, Cambridge University, Cambridge, UK; 8 Department of Medicine, University of Cambridge, Cambridge, UK; 9 MRC Mitochondrial Biology Unit, University of Cambridge, Cambridge, UK; 10 Present Address: Cancer Research UK National Biomarker Centre, University of Manchester, Manchester, UK

**Keywords:** Neurology, Dementia

## Abstract

A leading explanation for translational failure in neurodegenerative disease is that new drugs are evaluated late in the disease course when clinical features have become irreversible. Here, to address this gap, we cognitively profiled 21,051 people aged 17–85 years as part of the Genes and Cognition cohort within the National Institute for Health and Care Research BioResource across England. We describe the cohort, present cognitive trajectories and show the potential utility. Surprisingly, when studied at scale, the *APOE* genotype had negligible impact on cognitive performance. Different cognitive domains had distinct genetic architectures, with one indicating brain region-specific activation of microglia and another with glycogen metabolism. Thus, the molecular and cellular mechanisms underpinning cognition are distinct from dementia risk loci, presenting different targets to slow down age-related cognitive decline. Participants can now be recalled stratified by genotype and cognitive phenotype for natural history and interventional studies of neurodegenerative and other disorders.

## Abstract (web summary)

Cognitive variability with age has been examined in 21,051 recallable volunteers and has highlighted a potential role of microglia and glycogen metabolism in the individual differences in general cognitive ability.

## Main

By 2050, approximately 139 million people are expected to have dementia worldwide^1,2^. Although there has been recent therapeutic progress (lecanemab^3^ and donanemab^4^), the vast majority of new treatments shown to be effective in animal studies do not benefit patients when evaluated in large-scale clinical trials^5–7^. Several explanations have been proposed for the translational failure, including a limited understanding of the pathophysiology and animal models that do not accurately reflect the human disorder. However, a compelling explanation is that the new drugs are genuinely effective but have been evaluated too late in the disease course to have clinically meaningful impact. Therefore, there is an urgent need to understand the disease mechanisms during the preclinical and prodromal stages of neurodegenerative diseases and test new treatments at an early stage^8^, maximizing the potential to enhance the quality of life and reduce the societal burden of disease. This requires large cohorts of participants willing to be recalled for clinical and experimental studies, but despite major international efforts, studies specifically focused on dementia are typically in the order of a few thousands with low recallable capability^9–11^.

The National Institute for Health and Care Research (NIHR) BioResource in England was established to facilitate the recall of volunteers keen to engage in experimental medicine and clinical trials across the whole of medicine^12^. Most of the participants are healthy, are extensively phenotyped and have genome-wide genetic data available. Recognizing the unmet need to develop treatments for neurodegenerative disorders, we partnered with patients and carers from the UK Alzheimer’s Society to design and deliver the Genes and Cognition (G&C) cohort as an open-ended study nested within the NIHR BioResource. Individuals undertook cognitive profiling and genetic testing mirroring UK Biobank (UKB), enabling targeted recall studies in 21,051 NIHR BioResource participants from the UK population for both discovery and experimental validation. This also offers an opportunity to study the dynamics of cognitive variability across the lifespan and its genetic underpinnings. In this Article, we report the demographic, cognitive and genetic data available for participant recall, including educational status, measures of deprivation, comorbidities and 13 cognitive phenotypes. To show the potential power of the resource, we determine the heritability of each cognitive phenotypes, show phenotypic and genetic correlation between cognitive phenotypes, and determine the genetic landscape for two novel measures of cognitive ability, discovering novel genetic loci influencing cognitive performance throughout the life course.

## Results

### Participant data on demographics, cognition and genetics for recall

**Fig. 1:**
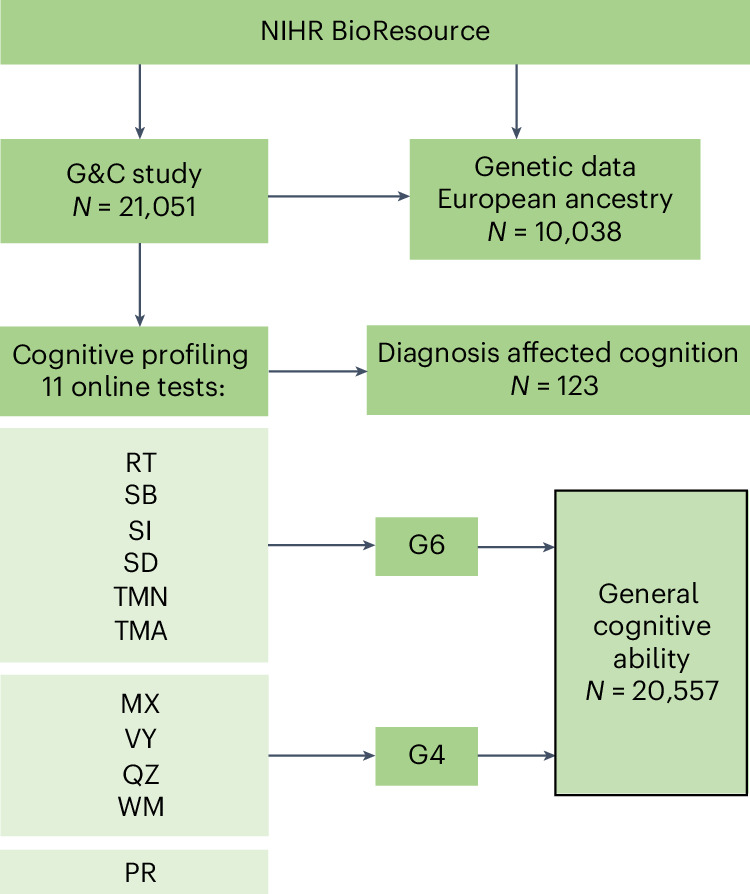
Study flow chart and derivation of two measures of general cognitive ability (G4 and G6). Diagnoses that affected cognition (*N* = 123) and participants with missing values in cognitive tests were excluded while measuring G4 and G6.

**Extended Data Fig. 1:**
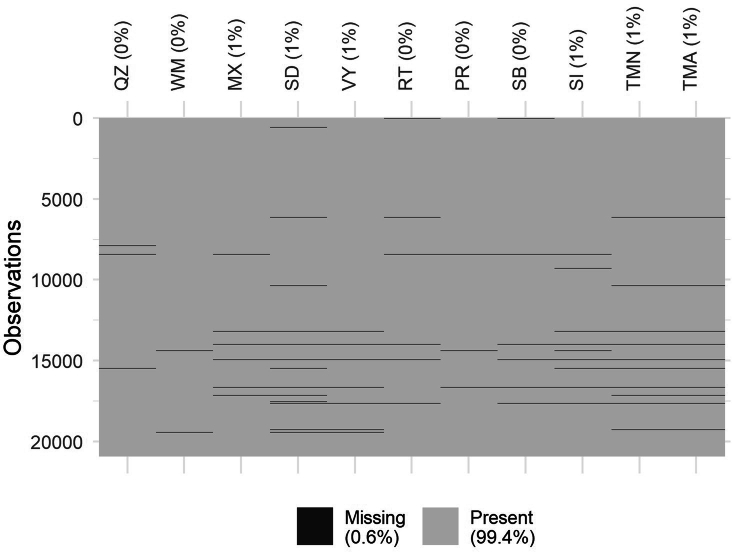
Proportion of missing values in cognitive test scores. QZ: Quiz; WM: Working memory; MX: Matrices; SD: Symbols Digit; VY: Vocabulary; RT: Reaction Test; PR: Pairing 7; SB: Stroop Box; SI: Stroop Ink; TMN: Trail Making Numeric; TMA: Trail Making Alpha Numeric.

**Extended Data Fig. 2:**
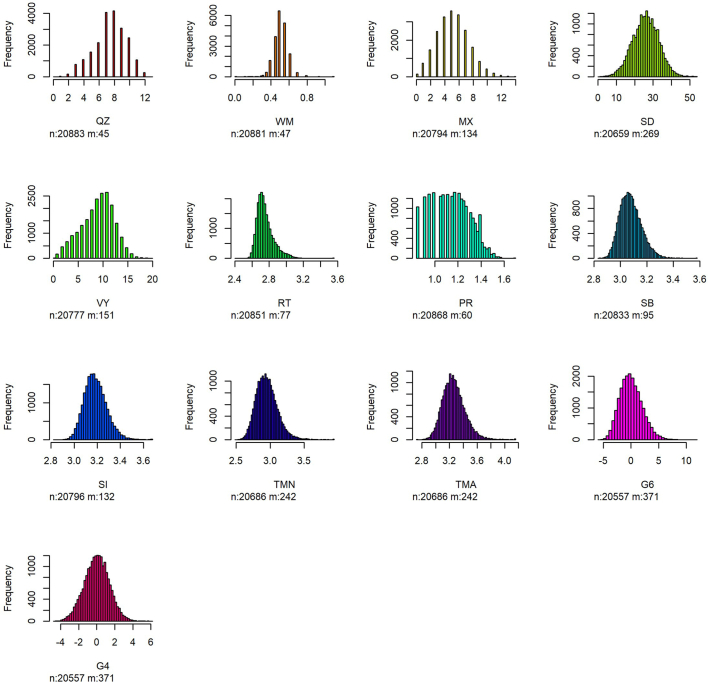
Distribution of cognitive test scores and two summary scores (G4 and G6). In the x-axis subtitle, n indicates the number of non-missing values and m indicates the number of missing values. QZ: Quiz; WM: Working memory; MX: Matrices; SD: Symbols Digit; VY: Vocabulary; RT: Reaction Test; PR: Pairing 7; SB: Stroop Box; SI: Stroop Ink; TMN: Trail Making Numeric; TMA: Trail Making Alpha Numeric.

Eleven cognitive tests (Reaction test, RT; Stroop box, SB; Stroop ink, SI; Symbol digits, SD; Trail making: numeric, TMN; Trail making: alpha numeric, TMA; Matrices, MX; Quiz, QZ; Vocabulary, VY; Working memory, WM; Pairing 7, PR) spanning different cognitive domains were undertaken at the participants’ convenience using downloaded software (Fig. 1 and Methods). The tests were those used in the Airwave study^13^ adapted to work on a range of different devices. Data from 21,051 participants were available (Table 1). Self-reported clinical information is presented in Supplementary Table 1, and a summary of 11 tests (phenotypes) is presented in Supplementary Tables 2 and 3, and Extended Data Figs. 1 and 2. Test scores from QZ (a measure of fluid intelligence), WM, MX, VY (a measure of crystallized intelligence) and SD were reversed so that higher scores indicate poorer performance, facilitating a direct comparison between all cognitive phenotypes. Those reporting a diagnosis known to affect cognition (*n* = 123) were excluded from subsequent analyses.

**Table 1:** Characteristics of G&C study participants

Characteristics	*N* = 21,051	Missing (%)
**Age**^a^ **(years)**, mean (s.d.)/median (IQR)	50.48 (14.81)/52 (39, 62)	—
**Age (years) category**, *n* (%)		
17–25	1,238 (5.9)	—
26–35	2,900 (13.8)	
36–45	3,439 (16.3)	
46–55	4,701 (22.3)	
56–65	5,084 (24.2)	
66–75	3,322 (15.8)	
76+	367 (1.7)	
**Gender**^a^, female/male/other, *n* (%)	13,298 (63.2)/7,692 (36.5)/61 (0.3)	—
**Ethnicity**^a^, *n* (%)		
African	73 (0.4)	5.4
Asian	148 (0.7)	
Mixed	283 (1.4)	
Other	126 (0.6)	
White	19,292 (96.8)	
**Smoking status**^a^, *n* (%)		
Current smoker	428 (5.4)	62.2
Nonsmoker	4,568 (57.4)	
Past smoker	2,959 (37.2)	
**Alcohol use**^a^ **(yes)**, *n* (%)	7,424 (84.4)	58.2
**BMI**^a^ **(kg** **m**^**−2**^**)**, *n* (%)		
Underweight (<18.5)	122 (1.4)	
Healthy weight (18.5–24.9)	3,812 (43.4)	58.3
Overweight (25–29.9)	3,144 (35.8)	
Obese (≥30)	1,709 (19.4)	
**Multiple deprivation index**, *n* (%)		
High (1–3)	3,474 (17.2)	4.2
Medium (4–7)	8,334 (41.3)	
Low (8–10)	8,351 (41.4)	
**Education**^**a**^, *n* (%)		
1 (lowest)	295 (3.9)	64.4
2	2,019 (27.0)	
3	748 (10.0)	
4 (highest)	4,427 (59.1)	
**Worked nights 72** **h before test**^a^ **(yes)**, *n* (%)	431 (2.0)	—
**First language is English**^a^, *n* (%)	20,082 (96.9)	1.5

^a^Self-reported in response to questionnaire provided either by NIHR BioResource or Cognitive Test application.

IQR, interquartile range.participants will be no different

**Extended Data Fig. 3:**
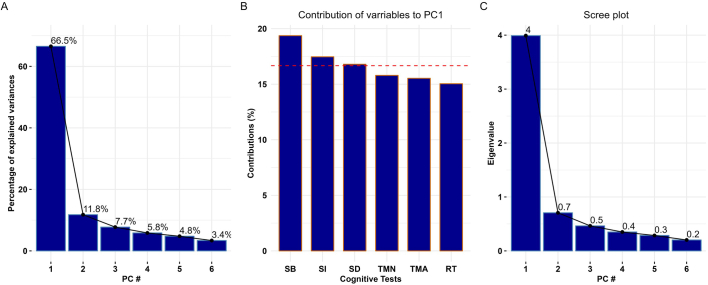
(**A**) Percentage of variance explained by principal components (PCs) derived from six cognitive tests (SB: Stroop Box, SI: Stroop Ink, SD: Symbols Digit, TMN: Trail Making Numeric, TMA: Trail Making Alpha Numeric, RT: Reaction Test), (**B**) Contribution of each of the six cognitive tests to PC1. Horizontal red (dashed) line expected value of each test if the contribution where uniform, and (**C**) Generalised scree plot for PCs derived from SB, SI, SD, TMN, TMA and RT tests.

**Extended Data Fig. 4:**
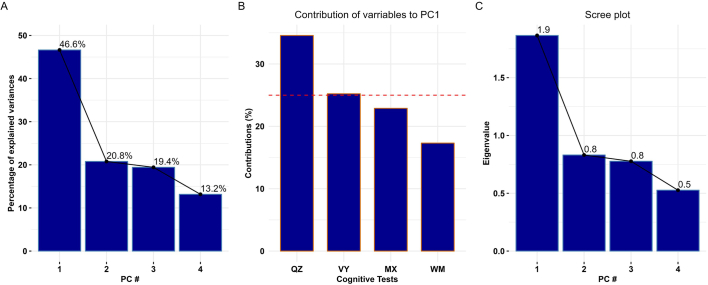
(**A**) Percentage of variance explained by principal components (PCs) derived from four cognitive tests (QZ: Quiz, VY: Vocabulary, MX: Matrices, WM: Working Memory), (**B**) Contribution of each of the four cognitive tests to PC1. Horizontal red (dashed) line expected value of each test if the contribution where uniform, and (**C**) Generalised scree plot for PCs derived from QZ, VY, MX and WM tests.

**Extended Data Fig. 5:**
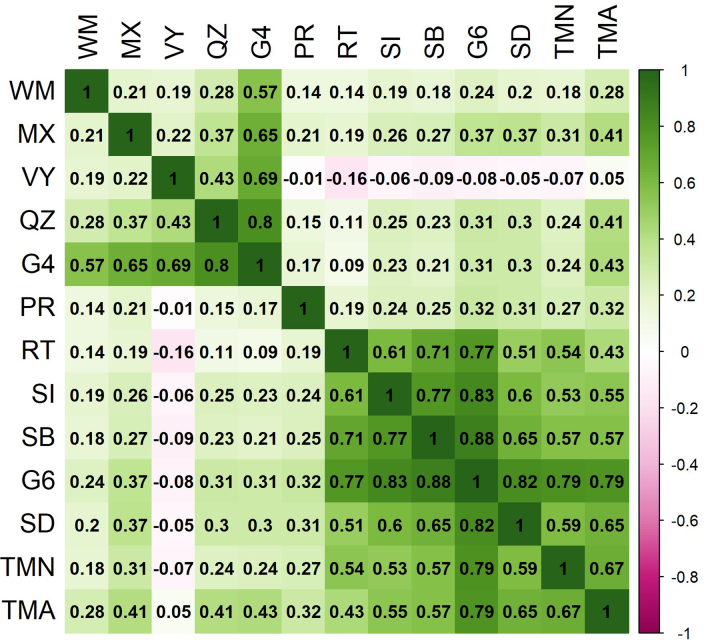
Phenotypic correlation for 11 cognitive test scores and two measures of general cognitive ability (G4 and G6). QZ: Quiz; WM: Working memory; MX: Matrices; SD: Symbols Digit; VY: Vocabulary; RT: Reaction Test; PR: Pairing 7; SB: Stroop Box; SI: Stroop Ink; TMN: Trail Making Numeric; TMA: Trail Making Alpha Numeric. Each coloured cell indicates magnitudes of phenotypic correlations. The corresponding colour scale is presented on the right side of the heatmap where dark green represents the highest genetic correlation, and darker moderate pink represents highest negative correlation. On the x- and y-axis, all cognitive phenotypes are presented maintaining the order.

Common variance underlying cognitive tasks is known as general cognitive ability, general intelligence or g-factor^14^. We obtained two data-driven measures of general cognitive ability (G6 and G4) using principal component (PC) analysis across participants based on disjoint subsets of the cognitive phenotypes (Methods and Extended Data Figs. 2, 3 and 4). G6 corresponds to the first PC (explaining 66.5% of variation) derived from RT, SB, SI, SD, TMN and TMA (Methods and Extended Data Fig. 3a–c). G4 corresponds to the first PC (explaining 46.6% of variation) derived from MX, QZ, VY and WM (Methods and Extended Data Fig. 4a–c). All 13 cognitive phenotypes (11 cognitive tests, G4 and G6) were positively correlated with each other except VY, which was positively correlated with QZ, MX, WM, TMA and G4, and negatively correlated with the other cognitive phenotypes (Extended Data Fig. 5).

**Extended Data Fig. 6:**
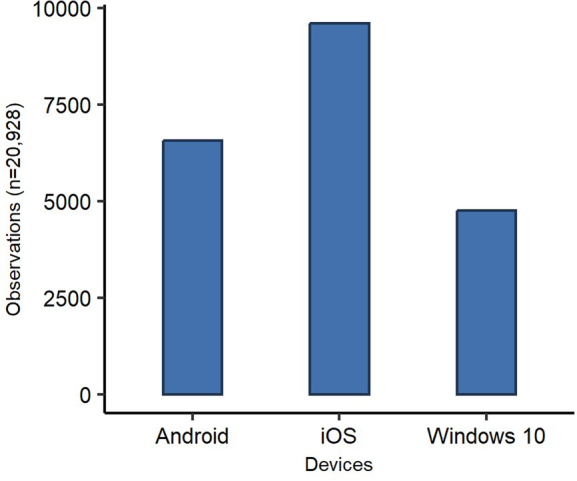
Devices used to take the cognitive tests. The x-axis represents types of devices used to take cognitive test and the y-axis represents number of observations.

**Extended Data Fig. 7:**
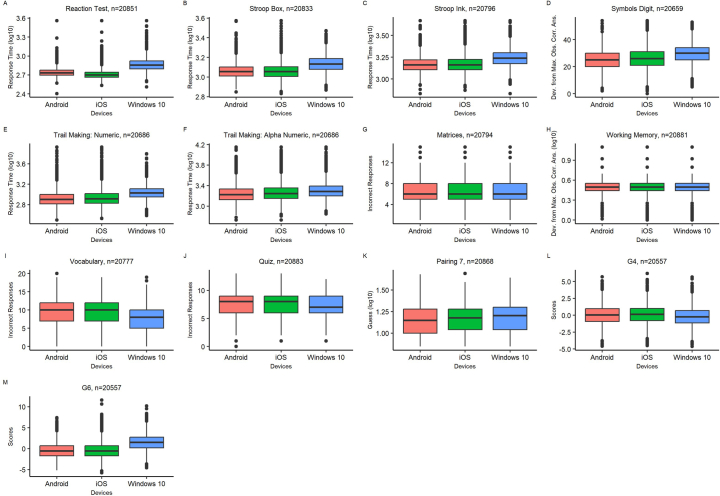
(A-K) Cognitive tests Scores by devices used to take the test and (L-M) Measures of general cognitive ability (G4 and G6) by device used to complete all cognitive tests. Across all plots, the x-axis represents types of devices used to take cognitive test and the y-axis represents score for the corresponding test or measures of general cognitive ability. Response Time is the average time taken per item. In each box plot (**A**–**L**), the box represents the interquartile range, with the center line denoting the median. The edges of the box indicate the first and third quartiles, while the whiskers extend to span a range of 1.5 interquartile distances from the edges. Individual data points that fall beyond the whiskers are presented as circles.

The majority of participants used iOS devices (46%), followed by Android (31%) and Windows (23%) devices to take the tests (Extended Data Fig. 6). With the exception of WM, there were systematic differences in test scores between the device types, which remained after adjusting for age and gender, possibly reflecting differences in input interface (touchscreen versus mouse; Extended Data Fig. 7 and Supplementary Table 4). The device type was thus factored into all subsequent analysis other than WM. Although there were differences in device use between different age, socioeconomic and educational groups (Supplementary Table 5), potentially influencing some of the cognitive phenotypes (except WM and PR). However, this should be borne in mind if participants are recalled on the basis of their cognitive profiles.

Available genome-wide genotype array data (based on UKB Axiom Array) confirmed the self-reported ethnicity (99.3%) in a subgroup of participants (*N* = 10,038) representative of the whole G&C cohort (Supplementary Tables 3, 6 and 7).

### Cognition, gender, education, deprivation and health

**Fig. 2:**
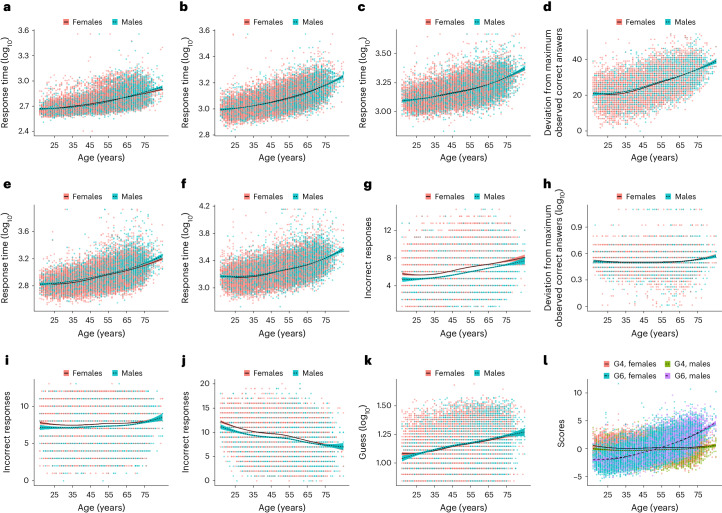
Cognitive tests and measures of general cognitive ability (G4 and G6) by age and gender. **a**–**l**, Cognitive test scores for RT (**a**), SB (**b**), SI (**c**), SD (**d**), TMN (**e**), TMA (**f**), MX (**g**), WM (**h**), QZ (**i**), VY (**j**) and PR (**k**) and G4 and G6 scores (**l**) plotted against age. Lines of best fit with standard error are stratified by gender (indicated by line color). Response time is the average time taken per item.

As expected, performance across all cognitive tests decreased with age, except VY, which increased with age (Bonferroni–Holm-adjusted *P* < 0.05; Fig. 2 and Supplementary Table 8). Previous reports have shown that VY performance declines beyond age 60 years^15,16^, but this was not apparent across 20,777 NIHR BioResource participants. Males had, on average, higher SD, TMN, TMA and PR scores, and lower scores in other phenotypes when compared with females (Bonferroni–Holm-adjusted *P* < 0.05; Fig. 2 and Supplementary Table 8) except for G6 where there was no clear evidence for a gender difference. A significant age-by-gender interaction effect was observed for SD, VY and G4 (Bonferroni–Holm-adjusted *P* < 0.05; Supplementary Table 8, model 1). An indication of age-by-gender interaction was observed for RT, SB and QZ. However, age and gender terms did not make a major contribution to the variance of WM (1.09%), QZ (1.16%) and G4 (2.53%). Although several previous studies reported differences in cognition between males and females, these have been inconsistent^17–22^. Here, we confirm that the overall pattern of cognitive change between males and females is strikingly similar, with gender only accounting for 0.1–1.33% of the variation in cognitive phenotypes. Adjusting for deprivation and ethnicity did not influence this analysis (Supplementary Table 8, model 2).

**Extended Data Fig. 8:**
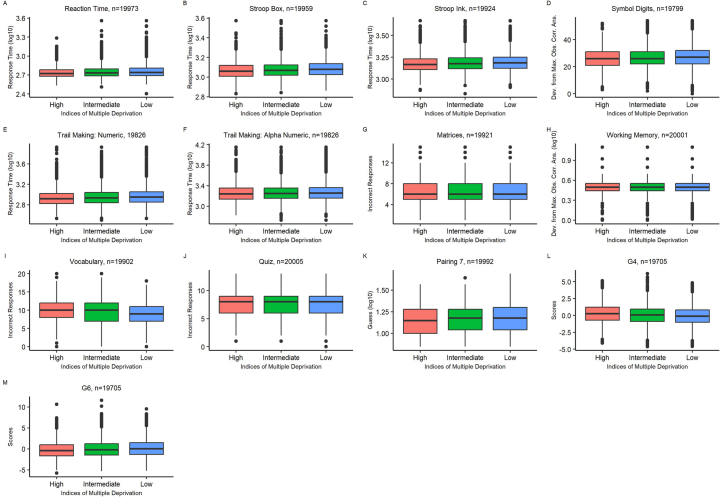
(A-K) Cognitive tests Scores by groups of multiple deprivation and (L-M) Measures of general cognitive ability (G4 and G6) by groups of multiple deprivation. Across all plots, the x-axis represents groups of multiple deprivation, and the y-axis represents score for the corresponding test or measures of general cognitive ability. Response Time is the average time taken per item. In each box plot (**A-L**), the box represents the interquartile range, with the center line denoting the median. The edges of the box indicate the first and third quartiles, while the whiskers extend to span a range of 1.5 interquartile distances from the edges. Individual data points that fall beyond the whiskers are presented as circles.

Likewise, in keeping with previous studies^23^, the lowest two education groups had higher scores (worse performance) across all cognitive phenotypes when compared with the highest education group (Bonferroni–Holm-adjusted *P* < 0.05; Supplementary Table 9), and there was a linear trend between cognitive performance and educational attainment (Bonferroni–Holm-adjusted *P* < 0.05; Supplementary Table 9). All cognitive phenotypes except PR correlated with levels of multiple deprivation (Bonferroni–Holm-adjusted *P* < 0.05; Extended Data Fig. 8 and Supplementary Table 10), with a significant linear trend indicating worse performance with higher levels of multiple deprivation (Bonferroni–Holm-adjusted *P* < 0.05). Associations between cognitive profiles and self-reported health-related issues are presented in Supplementary Table 11. Given the correlation between all of these parameters and cognition, these data have been made available for recall, allowing participants to be matched by potential confounders of cognition.

### Cognitive trajectory and *APOE* genotype

*APOE e4* allele status has a major impact on Alzheimer’s disease (AD) risk^24^. *APOE* genotype is also thought to influence cognition and brain activity in healthy individuals, but studies have been small, with inconsistent findings^25–29^. To show the utility of the NIHR BioResource G&C cohort, we determined whether *APOE* genotype influences cognitive performance throughout adult life.

**Extended Data Fig. 9:**
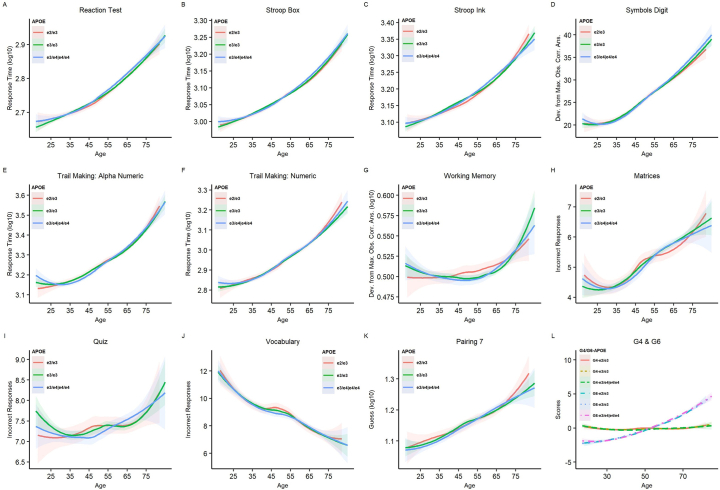
Cognitive tests and two measures of general cognitive ability (G4 and G6) stratified by APOE alleles status. (**A**–**K**) Cognitive test scores and (**L**) G4 and G6 scores plotted against age. Lines of best fit with standard error are stratified by APOE allele status (indicated by line colour). Response Time is the average time taken per item.

**Extended Data Fig. 10:**
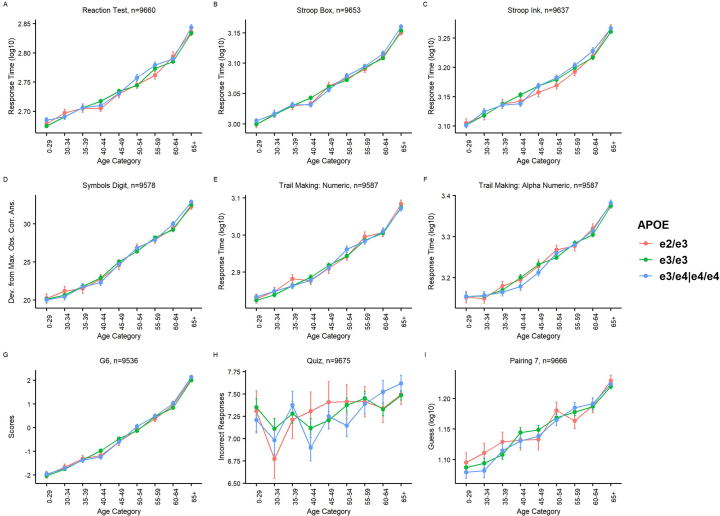
(A-I) Mean and standard error for nine cognitive phenotypes across age categories stratified by APOE alleles. In each of the plots, mean score differences for APOE allele carriers across different age groups were assessed using analysis of variance (ANOVA). The x-axis represents age categories and y-axis indicate scores for the corresponding cognitive phenotype. Bars are aligned based on age category and indicates standard error for the mean. The red, green, and blue lines across plots represent APOE allele status, specified in the right side of the plot with colour coded legend. Response Time is the average time taken per item.

*APOE e4* carriers showed a subtle increase in RT, SB, SI, SD, TMA, G6, QZ and PR emerging in late middle age (45–64 years) and TMN in late old age (>65 years) when compared with *e3*/*e3* carriers (Extended Data Fig. 9), but this did not withstand adjustment for covariates (Supplementary Table 12). On further inspection of those nine cognitive phenotypes showing subtle increase, RT, SB, SI and G6 showed a trend toward having pointwise higher mean scores for *e4* allele carriers after the age 45 when using categorized age (Extended Data Fig. 10). An age-by-*APOE* interaction was observed for SD and G6, where *e4* carriers had higher scores than *e3*/*e3* carriers (uncorrected *P* < 0.05), and an age^2^-by-*APOE* interaction effect was observed for SI, where *e2*/*e3* carriers had higher scores compared with *e3*/*e3* carriers (uncorrected *P* < 0.05; Supplementary Table 12). Previous studies reported associations with *APOE* for specific age groups, including 60–65 years^30,31^, and between 47 and 56 years^32^, particularly for processing speed (similar to SD) and visual episodic memory (similar to PR). However, in our study, none of these associations survived correction for multiple testing. In conclusion, across the age range studied we saw no compelling evidence that *APOE* genotype influenced performance of the 11 established cognitive phenotypes in the 9,691 individuals where the genotype could be unambiguously called (Methods).

### Stratification by AD polygenic risk scores

**Fig. 3:**
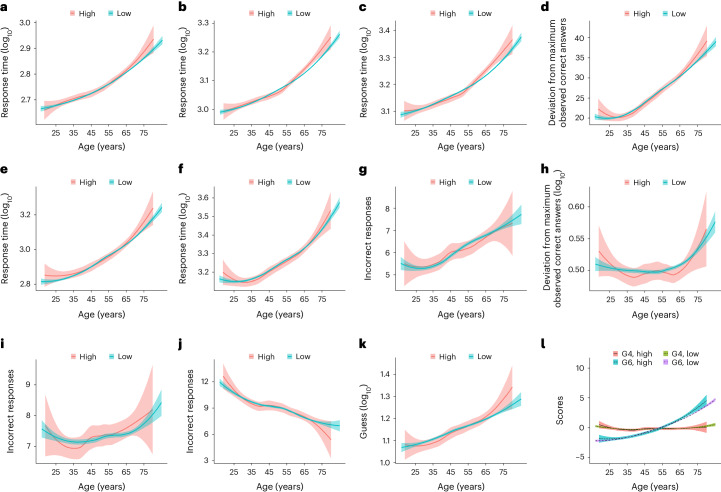
11 Cognitive tests and measures of general cognitive ability (G4 and G6) stratified by AD-PRS (including APOE region; AD-PRS_APOE_) group. **a**–**l**, Cognitive test scores for RT (**a**), SB (**b**), SI (**c**), SD (**d**), TMN (**e**), TMA (**f**), MX (**g**), WM (**h**), QZ (**i**), VY (**j**) and PR (**k**) and G4 and G6 scores (**l**) plotted against age. Lines of best fit with standard error are stratified by AD-PRS_APOE_ group (indicated by line color); The ‘high’ group is the top 5th percentile of AD-PRS_APOE_ and the ‘low’ group is the bottom 95th percentile of AD-PRS_APOE_. The response time is the average time taken per item.

Given the interest in polygenic risk scores (PRS) in AD risk stratification, AD-PRS were calculated for participants to facilitate informed recall. AD-PRS obtained from Lambert et al.^33,34^ were used to test whether AD genetic risk was associated with cognitive performance across the age range. Two PRS were created (Supplementary Table 13), one including *APOE* (AD-PRS_APOE_) and the other without *APOE* (AD-PRS_noAPOE_) to determine the value of non-*APOE* PRS in risk prediction. The 11 cognitive scores, G4 and G6 were compared between the top 5th percentile of AD-PRS (‘AD-PRS-high’ group) and the bottom 95th percentile of AD-PRS (‘AD-PRS-low’ group). For AD-PRS_APOE_, positive deviation in RT, SB, SI, SD, TMN, PR, QZ and G6 scores were observed for the AD-PRS_APOE_-high group starting between ages 55 and 65. A similar score deviation was observed around late adulthood (over 65 years) for TMA (Fig. 3). For AD-PRS_noAPOE_, a positive score deviation in RT, SB, TMN and VY was observed for the AD-PRS_noAPOE_-high group beginning in either late middle age or late adulthood (Supplementary Fig. 1). In the adjusted analysis, these score deviations did not differ between the AD-PRS_APOE_ (Supplementary Table 14) and AD-PRS_noAPOE_ groups (Supplementary Table 15). However, an age-by-AD-PRS_APOE_ risk group interaction was observed for SB, SI and G6 (Supplementary Table 14), but only the SI association remained following multiple testing corrections (Bonferroni–Holm-adjusted *P* = 0.039). Our exploratory analysis using categorized age showed that mean values for SB, SI, SD and G6 between AD-PRS_APOE_ groups differed (*P* < 0.05) for the 60–64-year-old age category (Supplementary Fig. 2). No age-by-AD-PRS_noAPOE_ risk group interaction effect was observed for RT, SB, TMN and VY (Supplementary Fig. 3). Thus, AD-PRS had a minimal impact on cognitive performance, with effects being noticeable only in later life. The use of AD-PRS had inferior discriminatory ability than the *APOE* genotype alone to identify early changes in cognitive ability.

### Heritability, genetic and phenotypic correlation

Having annotated the cohort for recall studies based on cognition and genotype, we moved on to estimate single-nucleotide polymorphism (SNP) heritability for each cognitive phenotype, as well as the genetic and phenotypic correlations between these phenotypes. Based on individual-level genetic data, the heritability of each cognitive phenotype ranged from 0.06 to 0.28 (Methods and Supplementary Tables 16 and 17), confirming published findings for QZ^35^, RT^36^, TMA^37^ and general cognitive ability^38^. The correlations between genetic profiles associated with cognitive phenotypes were stronger than the correlations between the cognitive phenotypes themselves (Methods and Supplementary Fig. 4a,b).

### Genome-wide association study of general cognitive ability

**Fig. 4:**
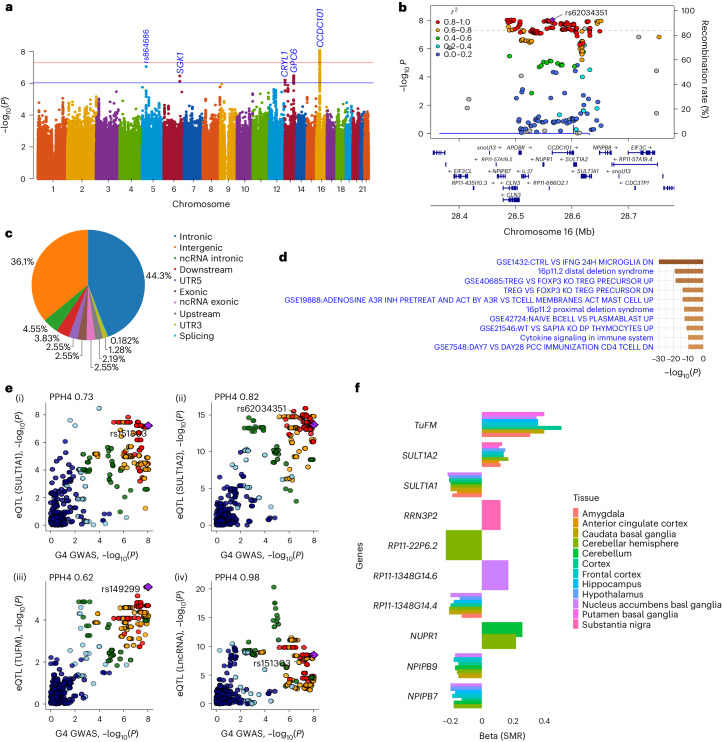
Genetic associations with G4 and likely functional relevance. **a**, A Manhattan plot of the genome-wide association analysis of G4. The *x* axis shows SNP chromosome positions, and the *y* axis shows the corresponding −log_10_ two-tailed *P* values from the two-sided BOLT infinitesimal model. The horizontal red line indicates the genome-wide significance threshold at *P* = 5 × 10^−8^. The horizontal blue line indicates the suggestive genome-wide significance threshold at *P* = 1 × 10^−6^. The nearest gene or top SNP is highlighted for loci associated at *P* < 1 × 10^−6^. **b**, Regional association and LD plots for G4-associated genome-wide significant locus. The *x* axis shows the SNP position on the chromosome, and the *y* axis shows the −log_10_(*P* value). The independent SNP is indicated by the purple diamond. The circles show other SNPs in pairwise LD with the independent SNP, with color indicating the strength of LD (*r*^2^). The strength of LD (*r*^2^) is presented in the upper left corner of the plot. The dashed horizontal line indicates genome-wide significant threshold. Estimated recombination rates are marked in light blue. Bottom: genes within ±200 kb of the independent SNP. **c**, A pie chart showing the proportion of the functional consequences of the G4-associated independent SNP and its proxies as annotated with ANNOVAR. **d**, Pathway and process enrichment analysis of genes mapped for G4 locus. The figure presents the top ten clusters along with their respective enriched terms (one per cluster). *P* values (−log_10_ transformed) are computed using the cumulative hypergeometric distribution, and the most statistically significant term within each cluster is selected to represent it. **e**, Colocalization of G4-associated signals with microglia eQTLs at SULT1A1 (i), SULT1A2 (ii), TUFM (iii) and long noncoding RNA (lncRNA) (iv). Each colored point indicates the strength of LD (red, ≥0.8; orange, 0.6–0.8; green, 0.4–0.6; light blue, 0.2–0.4; dark blue, <0.2) with candidate SNP (purple diamond labeled with rsID). PPH4 values indicate PP in support of shared single causal variants between the traits. PPH3 values indicate PP in support of sharing different causal variants between traits. **f**, A bar graph showing evidence from SMR between G4-GWAS and GTEx (v8) Brain eQTLs (*cis*) for G4-associated locus. The *x* axis represents coefficients from SMR for associated brain tissues (indicated by color), and the *y* axis represents prioritized genes.

**Fig. 5:**
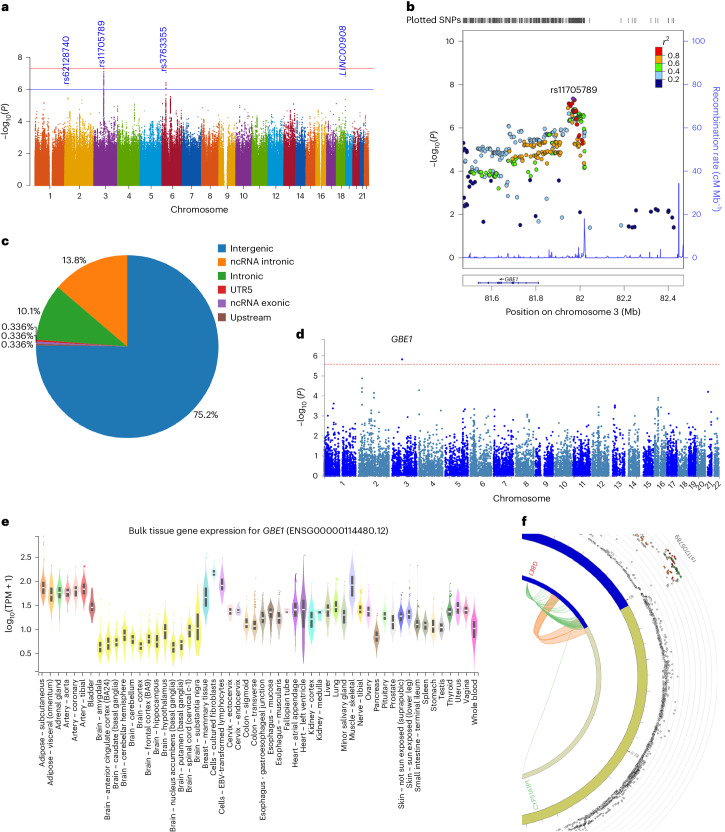
Genetic associatons with G6 and likely functional relevance. **a**, A Manhattan plot of the genome-wide association analysis of G6. The *x* axis shows SNP chromosome positions, and the *y* axis shows the corresponding −log_10_ two-tailed *P* values from the two-sided BOLT infinitesimal model. The horizontal red line indicates the genome-wide significance threshold at *P* = 5 × 10^−8^. The horizontal blue line indicates the suggestive genome-wide significance threshold at *P* = 1 × 10^−6^. The nearest gene or top SNP is highlighted for loci associated at *P* < 1 × 10^−6^. **b**, Regional association and LD plots for G6-associated genome-wide significant locus. The *x* axis shows SNP position on the chromosome, and the *y* axis shows −log_10_(*P* value). Tick marks at the top of the plot indicate SNP position. The independent SNP is indicated by the purple diamond. The circles show other SNPs in pairwise LD with the independent SNP, with color indicating the strength of LD (*r*^2^). The strength of LD (*r*^2^) is presented in the upper left corner of the plot. Estimated recombination rates are marked in light blue. Bottom: genes within ±500 kb of the independent SNP. **c**, A pie chart showing the proportion of the functional consequences of G4-associated independent SNP and its proxies as annotated with ANNOVAR. **d**, A Manhattan plot for the GWGBA analysis of G6. The *y* axis shows the −log_10_-transformed two-tailed *P* value of each gene from a linear model, and the *x* axis shows the chromosomal position. The dotted red line indicates the Bonferroni-corrected threshold (*P* = 2.614 × 10^−6^) for the genome-wide significance of the gene-based test. The gene with the lowest *P* value is highlighted. **e**, Bulk tissue expression of the *GBE1* gene across tissue types from GTEx v8. The *y* axis represents transcripts per million (TPM), and the *x* axis represents the GTEx (V.8) tissues. Box plots feature the median, 25th and 75th percentiles. Points are displayed as outliers if they fall beyond 1.5 times the interquartile range. The figure was adapted from the GTEx Portal (https://www.gtexportal.org/home/gene/GBE1). **f**, A circos plot displaying chromatin interactions (Ci) and eQTLs for *rs11705789*. The outermost layer shows the Manhattan plot with −log_10_(*P* value) for the G6-associated locus, and SNPs with *P* < 0.05 are displayed. The LD relationship between *rs11705789* and other SNPs is indicated with red (*r*^2^ > 0.8), orange (*r*^2^ > 0.6) and green (*r*^2^ > 0.4) colors. Gray SNPs show minimal LD with *r*^2^ ≤ 0.20. The second circle represents the chromosome ring with coordinates, where the genomic risk locus is highlighted in blue. The third circle shows the same chromosome ring, but with Ci- and eQTL-mapped genes represented by orange and green lines, respectively. Genes mapped by both approaches are colored red.

Given that G4 and G6 explained most of the variation seen in the individual tests (Extended Data Figs. 3 and 4), we conducted two genome-wide association studies (GWAS) to identify known or novel genetic loci determining general cognitive ability. Covariates included in the GWAS are listed in Supplementary Table 17. G4 and G6 were associated with distinct genome-wide significant loci (Figs. 4a and 5a and Supplementary Fig. 5). There was no evidence of confounding due to population stratification (G4: *λ*_GC_ = 1.0466, linkage disequilibrium score regression (LDSR)^39^ intercept 0.9974, and G6: *λ*_GC_ = 1.0466, LDSR intercept 1.0095), indicating that the different cognitive domains probably have different molecular bases. The strongest association for G4 spanned 75 SNPs (*P* < 5 × 10^−8^) including the independent SNP, *rs62034351* (intronic variant, *P* = 9.1 × 10^−9^), within *CCDC101* (*SGF29*) in a gene-dense region on chromosome 16 (Fig. 4b and Supplementary Tables 18 and 19). *Rs62034351* explained 185-fold more of the variance in G4 (0.37%, analysis of variance (ANOVA) *P* = 1.38 × 10^−8^) than *APOE* (0.002%, ANOVA *P* = 0.93). Four additional loci were suggestive of genome-wide association with G4 (*P* < 1 × 10^−6^; Supplementary Table 20). For G6, the strongest association was on chromosome 3, with the independent SNP at this locus (*rs11705789*; *P* = 4.5 × 10^−8^) near *GBE1* (Fig. 5b and Supplementary Tables 18 and 21). Three additional loci were suggestive of an association with G6 (Supplementary Table 22). *Rs11705789* explained 5.5-fold more variance in G6 (0.11%, ANOVA *P* = 2.52 × 10^−5^) than *APOE* (0.02%, ANOVA *P* = 0.21). To validate these findings, we reviewed two previous meta-analyses of intelligence^40,41^. The G4/*rs62034351* discovery replicated in the same direction in both studies^40,41^, but the G6/*rs11705789* discovery did not replicate, possibly reflecting differences in the cognitive profiling and its contribution to G6 (Supplementary Table 23).

### Functional mapping of the G4 locus

SNPs in linkage disequilibrium (LD) with G4/*rs62034351* were annotated using ANNOVAR (*n* = 423). The majority of SNPs were intronic (44.3%) or intergenic (36.1%), but 14 lay within exons of which 7 were predicted to change the amino acid sequence (Fig. 4c and Supplementary Table 24). Thirteen SNPs (3.7%) were predicted to be deleterious (combined annotation-dependent depletion (CADD)^42^ score >12.37), 17 (4%) were likely to regulate gene expression (Regulome DB^43^ (RDB) score <2) and 385 (91.25%) had regulatory potential (minimum chromatin state <8). Genome-wide gene-based association (GWGBA) analysis identified 16 genes associated with G4 (*CLN3* was the highest ranked; Supplementary Fig. 6). Collectively, GWGBA, positional, expression quantitative trait loci (eQTL) and chromatin interaction mapping identified 128 genes for G4, including *NUPR1*, *ATXN2L*, *CCDC101* and *SULT1A1* observed through all mapping strategies (Supplementary Table 25 and Supplementary Fig. 7).

To cast light on the mechanisms underpinning G4 we investigated tissue-specific expression of the mapped gene set for 53 specific GTEx (v8)^44^ tissue types. Most of the implicated genes were downregulated across multiple tissues, particularly in the brain (Supplementary Fig. 8). The majority of the top 10 enriched terms identified by pathway and process analysis were immunological, with microglial response to γ-interferon being the highest ranked (Fig. 4d and Supplementary Table 26) and INTERFERON_GAMMA_RESPONSE being the top hallmark gene set (*P* = 3.68 × 10^−19^; Supplementary Fig. 9). In keeping with this, SNPs associated with G4 also influenced the expression of *TUFM*, *SULT1A1* and *SULT1A2* in microglia (microglial eQTLs^45^; Fig. 4e). To investigate whether the effects of G4 were restricted to different anatomical locations in the brain, we performed summary-based Mendelian randomization (SMR) analysis using GTEx (v8) eQTL on G4-GWAS summary statistics on tissue from 12 brain regions. This indicated a potential causal link between SNVs in 11 genes (seven protein coding), including *TUFM* (seven brain regions), *SULT1A1* (eight brain regions) and *SULT1A2* (eight brain regions), and G4-cognitive phenotype through differential microglial gene expression (Fig. 4f). Statistical fine mapping identified *rs3743963*, *rs11074904*, *rs62031607* and *rs2411453* as most plausible causal variants (Supplementary Fig. 10).

### Functional mapping of the G6 locus

A total of 186 SNPs in LD were annotated for the G6/*rs11705789* locus. The majority of the SNPs were intergenic (Fig. 5c). Nine SNPs (4.83%) were predicted to be deleterious, and 152 SNPs (81.72%) were identified with regulatory potential. GWGBA analysis identified *GBE1* as the only associated gene (Fig. 5d). The overall expression of *GBE1* was lower in all bulk brain tissues than the other tissue types (Fig. 5e). Independently, positional, eQTL and chromatin interaction mapping also prioritized *CYP51A1P1*, *RP11-359D24.1* and *RP11-142L1.1*, none of which are protein coding. G6/*rs11705789* is an expression quantitative locus for *GBE1* (Fig. 5f). There was no instrumental variable available for *GBE1* locus precluding SMR analysis. Statistical fine mapping showed *rs12635671*, *rs820270* and *rs2691073* to be the likely causal variant regulating *GBE1* expression.

### Correlation of general cognitive ability and related phenotypes

To assess the life course stability of general cognitive ability, we examined the association of G4 and G6 with childhood^46^ and adulthood^40,41^ intelligence quotient using GWAS summary statistics. Childhood and adulthood intelligence quotient had a high genetic correlation (GC) with G4 and G6, and the estimate for G4 was higher than G6 (Supplementary Table 27), suggesting that fluid and crystallized intelligence domains might be less variable within an individual across the life course than processing speed and executive function. We assessed the relevance of G4 and G6 in educational attainment^47^ using GC. G4 had a 2.4 times higher GC estimate with educational attainment than G6 (Supplementary Table 27), indicating that fluid and crystallized intelligence domains might predict better educational attainment than processing speed and executive function. We also looked for a GC between summary measures of cognitive abilities (G4 and G6) and AD^34^. A strong GC would imply a shared biological processes between two phenotypes^48^ (in this instance, cognition in healthy people and AD). However, our analysis only revealed a very weak correlation between the genetic factors associated with normal cognition and genetic factors associated with AD (Supplementary Table 27), implying different underlying biological mechanisms.

## Discussion

Here, we report cross-sectional data for 11 cognitive tests and two summary statistics (G4 and G6) in 20,928 healthy individuals aged 17–85 years who participated in the newly established NIHR BioResource G&C cohort. Analyzing data at this scale confirmed well-established determinants of cognition, including age, socioeconomic status and educational status, and showed negligible differences in cognitive performance between males and females across the life course. Contrary to previous reports from smaller studies, genetic risk factors for dementia, including *APOE* genotype and AD-PRS, have a minimal impact on cognition in healthy individuals. However, a small effect of e4 and AD-PRS on cognitive performance in certain domains emerges in mid-life, potentially reflecting the presence of patients with early AD neuropathological changes or demographic characteristics of the study influencing the e4-mediated effect on cognition. On the other hand, our unbiased genome-wide approach identified novel risk factors for different cognitive parameters. Thus, the genetic and biological basis of cognition in healthy individuals appears to be distinct from the pathogenesis of neurodegenerative dementia, and characterizing the different molecular pathways has the potential to uncover new targets to prevent age-related cognitive decline.

For G4, which summarizes short-term memory, fluid and crystallized intelligence, our functional annotation implicated microglial-mediated immunological processes in the age-related cognitive trajectory, supporting previous circulating cytokine measurements^49,50^. Multiple lines of evidence implicated three plausible genes (*TUFM*, *SULT1A1* and *SULT1A2*) with G4. *TUFM* encodes the mitochondrial elongation factor Tu, which is involved in mitochondrial protein synthesis and has been implicated with cognitive trajectory^51^ and AD pathology^52^. *SULT1A1* (sulfotransferase family 1A member 1) and *SULT1A2* (sulfotransferase family 1A member 2) encode sulfotransferase enzymes responsible for the metabolism of hormones, and xenobiotics^53^. While the functional roles of *SULT1A1* and *SUKT1A2* in the brain remain largely unexplored, both genes are expressed in the adult brain and are implicated in the local metabolism of catecholamines and toxin clearance^54,55^. However, the region is genetically complex, raising the possibility that other genes play a critical role through LD with the four likely causal SNVs: *rs3743963*, *rs11074904*, *rs62031607* and *rs2411453*. The locus also contains *IL27* coding for interleukin 27, which can be both pro-inflammatory and anti-inflammatory^56^ and influence microglial activation^57^. In addition, several proximal candidates have been implicated with brain function and cognition such as *CLN3*^58^, *KIF22*^59^, *ALDOA*, *SEZ6L2* and *TAOK2*^60,61^. Functional studies are required to clarify whether these genes play a role in general cognition, but this will be very challenging because phenotypes in cellular or animal models are unlikely to closely reflect cognitive function in healthy humans as they age.

For G6, which summarizes reaction time, attention, processing speed and executive functioning, only one protein-coding gene was associated with cognition: *GBE1*, which codes for 1,4-α-glucan-branching enzyme and plays a critical role in glycogen synthesis and glucose storage. Rare recessive mutations in *GBE1* cause adult polyglucosan body disease, which often affects cognition including executive function^62–64^, and in a recent GWAS, *GBE1* was implicated in musical beat synchronization^65^, which is closely related to attention and executive function (planning, organizing and controlling action). These independent observations support our findings indicating that *GBE1*—and more broadly, glycogen metabolism—probably play a role in general cognitive ability. Glycogen’s presence in the brain has not been considered to be as important as glucose, but its role in cognition has attracted recent interest^66–68^, warranting further investigation.

The strengths of this resource include online cognitive assessment allowing rapid data collection of thousands of individuals, cognitive phenotyping covering various domains, and genotyping mirroring the UKB. However, unlike UKB, the NIHR BioResource is designed specifically for participant recall, which is now possible based on both cognitive and genetic profiles. Several limitations also require consideration. So far, the cognitive data are cross-sectional, and measurement error may have diluted associations. The cognitive tests were also device dependent. Although this was taken into account in our analysis, this could confound recall studies unless factored into subsequent designs. It is important to note that our choice of cognitive tests does not represent all possible cognitive domains, such as verbal episodic memory and visuospatial skills. In addition, our findings are based on an analysis of participants of white European background, with the majority having benefited from higher education. Thus, our findings cannot be generalized across all ethnicities with confidence at this stage. Finally, it is important to note that, other than genetic and cognitive characterization, we have not yet measured any biomarkers specific for neurodegenerative diseases. It is therefore possible that recalled participants will be no different from the background population for specific neurodegeneration biomarkers such as brain imaging. On the other hand, this emphasizes the potential utility of the NIHR BioResource for a wide range of studies beyond neurodegeneration, including age-related cognitive decline and other common human disorders.

Our analyses of APOE genotypes and AD-PRS and G4 and G6 were chosen to illustrate the potential use of the data generated through the NIHR G&C study. However, the potential for further analysis extends way beyond what has been explored so far. The participants of the NIHR BioResource G&C cohort have consented to be recalled for clinical studies and clinical data linkage from across England. Defining the principal demographic and genetic factors that explain why any two individuals differ allows careful matching of participants in early proof-of-concept clinical trials, thus reducing the risk of confounding variables influencing experimental studies. It is also possible to recall specific genetic subgroups to optimize the chance of observing a specific treatment effect based on known mechanisms of action. We are currently repeating the cognitive profiling of all participants to determine cognitive trajectories over time, expanding to include more diverse ethnic groups and carrying out long-read genome sequencing to enrich the recall potential for both academic and industry researchers. The data access procedure for the NIHR BioResource is described at https://bioresource.nihr.ac.uk/using-our-bioresource/apply-for-bioresource-data-access/, and the participant recall process for the NIHR BioResource is explained at https://bioresource.nihr.ac.uk/using-our-bioresource/apply-for-recall/.

## Methods

### Study population and data collection

The G&C study is a prospective open cohort nested within the NIHR BioResource, which recruits participants from the general population and National Health Service organizations in England. The G&C study participants were recruited via NIHR BioResource with the objective of gaining insights into brain and cognitive function within healthy populations and facilitating early experimental studies in people at risk of neurodegenerative diseases such as dementia.

The NIHR BioResource operates under two separate set of ethics: a study for the recruitment of patients with rare disease (REC REF: 13/EE/0325) and a research tissue bank for the recruitment of all other participants (REC REF: 17/EE/0025). All participants of NIHR BioResource were invited to take part in the G&C study in two phases: (1) pilot phase (~June 2020 to ~August 2020) and (2) main phase (~November 2020 to ~November 2021). A total of 315 participants took part in the pilot study, and 20,869 participants participated in the main study. Combining both phases (excluding those who withdrew their consent or were missing vital information), 21,052 participants served as the study base. These participants were considered cognitively healthy at the time of recruitment for the G&C study. They donated their DNA via a blood sample and completed a questionnaire containing basic lifestyle and health-related information, including self-reported height and weight, ethnicity, current smoking status, alcohol consumption and diagnosis of certain diseases (for example, diabetes, stroke and mental health issues), all at recruitment to NIHR BioResource. Ethical approval for the G&C study was obtained from the North of Scotland Research Ethics Committee (REC REF: 19/NS/0118). All participants consented to be part of NIHR BioResource and to be recalled for future studies.

### Cognitive tests and measures of general cognitive ability

The G&C study participants were invited to take online cognitive tests using the ‘Cognitive Test (v4.4.7-v5.6.7)’ application that was downloadable onto a compatible device. The ‘Cognitive Test’ application was composed of a short pretest questionnaire and ten cognitive tests (RT; SB; SI; SD; Trail making: TMN and TMA; MX; WM; QZ; VY; and PR). The total time to complete all these tests was approximately 30 min. We reversed some test scores to make the direction of all tests similar. In this work, a higher score across cognitive tests signifies poorer performance. The majority of these tests are similar to cognitive tests performed in UKB. The 4-week test–retest reliability of the UKB cognitive tests was moderate to high (range 0.40–0.83), with most showing a modest to good correlation with reference datasets^69^. A brief discussion of each test and measures of general cognitive ability (G4 and G6) is presented in Supplementary Note.

### Other covariates

Information on age, gender, body mass index (BMI), self-reported ethnicity, smoking status, alcohol use and multiple deprivation index was collected centrally by NIHR BioResource. Age reflects the age at the time of cognitive testing. In this work, we used age as both a continuous and categorical variable. For the continuous use, age was centered by subtracting off the mean age in the G&C cohort, which was used to create a second-degree polynomial term. Self-reported gender was categorized as male, female and other. We categorized BMI into underweight (<18.5 kg m^−2^), healthy weight (18.5–24.9 kg m^−2^), overweight (25–29.9 kg m^−2^) and obese (≥30 kg m^−2^), following the criteria of the World Health Organization^70^. The multiple deprivation index is a relative measure of deprivation assigned to each participant on the basis of post codes^71^. Deprivation indices were available in deciles, where higher score correspond to lesser deprivation. In this study, we categorized deciles of multiple deprivations into three groups: (1) high (first three deciles), (2) medium (fourth to seventh deciles) and (3) low deprivation (eighth to tenth deciles). Information on education and participants’ first language was collected using the ‘Cognitive Test’ application. We categorized education into four groups, where the first category represents the lowest level of education and covers certificates of secondary education (CSEs)/equivalent/equivalent or other professional degrees/not specified. The second category covers A-level/O-level/national vocational qualification (NVQ)/higher national diploma (HND)/higher national certificate (HNC)/equivalent education, while the third category covers A-level/O-level/NVQ/HND/HNC/equivalent education with a professional degree and the fourth category covers college/university/equivalent professional degree.

### Self-reported diagnosis

Several self-reported diagnoses were available for G&C study participants. Information on arthritis, diabetes, the presence of autism, attention-deficit/hyperactivity disorder, any heart condition, high blood pressure, mental health issues and stroke or related conditions was collected using a questionnaire centrally by NIHR BioResource. Information on color blindness, learning disability and conditions that participants thought would interfere with their cognition was collected via the ‘Cognitive Test’ application before cognitive testing.

### Genotyping, imputation and quality control

DNA was extracted from whole blood and/or saliva. Aliquoted samples were sent to Affymetrix for genotyping and processing with the standard pipeline. Participants were genotyped using either Affymetrix v1.0 or v2.1 array by ThermoFisher Scientific^72^. Samples on the v1.0 and v2.1 chips were genotyped on the genome build hg37 and hg38, respectively. Before the imputation, we lifted variants on the genome build hg38 to hg37 using Liftover^73^ and 708,654 variants common in both chips were used for pre-imputation quality control. Genotyped markers were used to infer genetic sex and determine European ancestry (EU) using the 1000 Genomes dataset. The multidimensional scaling approach incorporating the 1000 Genomes dataset was used to infer the genetic ethnicity of the samples. Plink 1.9 (ref. ^74^) was used for multidimensional scaling analysis^75^. Only genetically inferred EU participants were used for imputation. We applied the following filters before the imputation: minor allele frequency (<0.01), marker missingness (>0.01), individual missingness (>0.01), Hardy–Weinberg equilibrium (*P* < 1 × 10^−6^), exclusion of individuals with extreme heterozygosity (±3 standard deviations (s.d.) from the mean heterozygosity rate) and exclusion of mono-morphic variants and those who had an allelic mismatch with Haplotype Reference Consortium (HRC)^76^. A total of 518,164 high-quality autosomal markers (genotyping rate 99.6%) were used for imputation using the HRC reference panel on the Michigan imputation server^77^. HRC consisted of whole-genome sequence data from cohorts of EU ancestry, providing large coverage for the common genetic variants in European ancestry population. To analyze the samples with genetic data, we excluded participants for whom there was a mismatch between genetically inferred sex and self-reported gender. To account for population stratification, 20 genetic PCs were created using post-imputation quality-controlled data, implemented on Plink 1.9 (ref. ^74^).

### Identification of *APOE* alleles

We use *rs429358* and *rs7412* to determine *APOE* alleles^78^. Both SNPs were imputed in our data. We used the method specified at GitHub (https://github.com/neurogenetics/APOE_genotypes). There were 279 participants for whom the *APOE* allele was ambiguous or unknown, and these were therefore excluded. In the remaining sample, the proportion of *e2*/*e2*, *e2*/*e3*, *e3*/*e3*, *e3*/*e4* and *e4*/*e4* carriers was 0.01 (*n* = 69), 0.13 (*n* = 1,238), 0.61 (*n* = 5,931), 0.24 (*n* = 2,304) and 0.02 (*n* = 218), respectively. We combined e4 carriers into one group (*e3*/*e4* and *e4*/*e4*, *n* = 2,522) and e2 carriers into another group (*e2*/*e2* and *e2*/*e3*, *n* = 1,307).

### Derivation of AD-PRS

The PRS provides an individual-level estimate of genetic liability for any given phenotype. The PRS is measured by combining weighted effect sizes (odds ratios or *β*) of multiple SNPs into one score, where weights are obtained from previous GWAS performed for that phenotype of interest^79^. The most widely used PRS for AD is obtained from Lambert et al.^34^ study, which included EU ancestry participants. We used previously created PRS based on Lambert et al.^34^ from the polygenic score (PGS) catalog. The PGS ID PGS002289 included 23 SNPs, of which *rs11218343*, *rs670139* and *rs8093731* were not available for G&C study participants. Of the 20 available SNPs, *rs429358* and *rs7412* represent APOE. We created two PRS using PGS ID PGS002289 (refs. ^32,33^), (1) AD-PRS_APOE_: including 20 SNPs (2 *APOE* SNPs included) and (2) AD-PRS_noAPOE_: including 18 SNPs (without *APOE* SNPs). Assuming an additive model, both PRS were computed using PRSice-2 (v2.3.3)^80^ with the ‘--score std’ and ‘--missing MEAN_IMPUTE’ settings. For both PRS (AD-PRS_APOE_ and AD-PRS_noAPOE_), we categorized participants into high-risk (values >95th percentile) and low-risk (values ≤95th percentile) groups (AD-PRS-high and AD-PRS-low).

### Statistical Analysis

Demographics, clinical characteristics and scores for 13 cognitive phenotypes (11 cognitive tests, G4 and G6) are presented for both the whole sample and a subset with available genetic data. Categorical data were presented as proportions, while continuous data were summarized using mean, median, s.d. or interquartile range. A small number of individuals (*n* = 123 out of 21,051) were excluded because they had a medical disorder or disability that could bias the effect estimates. The phenotypic correlation between cognitive phenotypes was measured using Pearson correlation (the whole sample and a subset with available genetic data). The association between 13 cognitive phenotypes and devices used (iOS device user served as reference category) to take cognitive tests was examined using a linear regression model with further adjustment for age and gender. Age and gender effects on cognitive phenotypes were measured, excluding those who self-identified as ‘other’ (*N* = 61). Trajectories of each cognitive phenotype (11 tests, G4 and G6) were plotted across age, stratified by gender, using the ‘geom_smooth’ function from the ggplot2 package in R with the ‘method’ argument set to ‘loess’. The associations of cognitive phenotypes were assessed in relation to age and gender. While testing associations, age (centered), age^2^, gender, an interaction term for age-by-gender and age^2^-by-gender, and devices used to take cognitive tests (except WM) were considered as covariates in a stepwise linear regression model using the ‘stepAIC’ function with both forward and backward selection implemented with MASS package in R to choose the best model for each cognitive phenotype. Henceforth, variables selected using stepwise regression (base model) remained consistent for each cognitive phenotype while testing association in relation to other factors, unless stated otherwise. Additionally, base models were adjusted for self-reported ethnicity and multiple deprivation. Since self-reported ethnicity and multiple deprivation had negligible effects on the cognitive phenotypes, none of the associations tested from this point onward included those factors. We assessed the association for cognitive phenotypes with education and multiple deprivation using linear regression model adjusting for the cognitive phenotype-specific base model. A linear trend in the association between cognitive phenotypes and both education and multiple deprivation was also examined. The association between cognitive phenotypes and self-reported diagnosis was explored using the linear regression model, which was adjusted for age terms, gender and device used to take the test. The association of cognitive phenotypes with age, gender, education, multiple deprivation and the self-reported diagnosis was corrected using the Bonferroni–Holm correction for 13 tests (considering the 13 cognitive phenotypes). The terms age^2^, age-by-gender, or age^2^-by-gender were corrected (Bonferroni–Holm) in accordance with the number of times they were subjected to testing against cognitive phenotypes.

Each cognitive phenotype was plotted against age, with the smooth line fitted and stratified by the *APOE* allele. Following visual inspection, nine cognitive phenotypes were selected to undergo testing for their association with age term(s) and *APOE* utilizing the linear mixed-effects model adjusting for sex (genetically determined), devices used for cognitive tests, genotyping batch as a random effect, genotyping array and first five genomic PCs. We used *e3*/*e3* carriers as a reference while assessing the association between cognitive phenotypes and *APOE*. The model also examined the interaction effect between age term(s) and *APOE* on cognitive phenotypes. The results of the linear mixed-effects model were corrected using Bonferroni–Holm correction for nine tests. Furthermore, the mean difference in all nine cognitive phenotypes across different age groups was explored using the ANOVA test.

The correlation between PRSs was measured using Pearson correlation. Cognitive phenotype trajectories across the age continuum (fitted smooth line) were inspected for an indication of score deviation in the AD-PRS-high group compared with the AD-PRS-low group. Based on the observations, the association for candidate cognitive phenotypes in relation to age term(s) and AD-PRS group (high versus low) was examined using the linear mixed-effects model adjusting for sex, devices used for cognitive tests, genotyping batch as a random effect, genotyping array and the first five genomic PCs. The model also assessed the interaction effect of age term(s) and AD-PRS group for each cognitive phenotype. The findings were presented following the Bonferroni–Holm correction. Based on the outcome of linear mixed-effects models, the mean difference in four cognitive phenotypes (for each PRS) were explored across age groups between AD-PRS-high and AD-PRS-low groups (based on AD-PRS_APOE_ and AD-PRS_noAPOE_) using *t*-tests.

### Heritability and GC analysis

We used individual-level genetic data to estimate SNP heritability and GC for 13 cognitive phenotypes. SNP heritability for cognitive phenotypes was estimated using BOLT-REML (V.2.4)^81,82^. Covariates adjusted in the heritability analysis are specified in Supplementary Table 17. GC between cognitive phenotypes was measured using Bivariate GREML analysis on GCTA (v1.94.1)^83^. Before the analysis, we removed related individuals using the ‘--grm-cutoff’ value of 0.125. For each cognitive phenotype, residuals were obtained from the separate linear regression model adjusted for covariates (except batch, genotyping chips and genetic PCs) specified in Supplementary Table 17. These residuals were used for GC analysis, which was adjusted for batch, genotyping chips and the first ten genomic PCs as covariates. Moreover, we measured summary statistics based GC for G4 and G6 in relation to childhood^46^ and adulthood^40,41^ intelligence, educational attainment^47^ and AD^34^ using LDSR (v1.0.1)^39^. Precomputed LD scores based on 1000 Genomes European data restricted to HapMap release-3 SNPs (*n* = 1,217,311) were used to calculate SNP heritability and GCs. Precomputed LD scores and the list of HapMap3 SNPs were obtained from https://data.broadinstitute.org/alkesgroup/LDSCORE/eur_w_ld_chr.tar.bz2 and https://data.broadinstitute.org/alkesgroup/LDSCORE/w_hm3.snplist.bz2.

### GWAS of general cognitive ability

We performed GWAS on G6 and G4 using the linear mixed model implemented in BOLT-LMM (V.2.3.6)^81^, which accounts for population structure and cryptic relatedness. These analyses were performed assuming an additive SNP effect on both phenotypes. Covariates adjusted for in the genome-wide association analysis of G4 and G6 are specified in Supplementary Table 17. We applied the following filters for the genome-wide association analysis of G4 and G6: minor allele frequency ≥0.05, imputation quality scores (INFO) ≥0.50, and HWE threshold *P* value <1 × 10^−6^. A *P*-value threshold of 5 × 10^−8^ (for suggestive significance, *P* value <1 × 10^−6^) was used to determine genome-wide significance. LDSR (v1.0.1)^39^ was used to assess inflation (*λ*_GC_) and to distinguish confounding from polygenicity in GWAS summary statistics. SNPs with *P* value <1 × 10^−5^ at each genome-wide significant locus were considered to identify independent SNP at *r*^2^ ≥ 0.4 using the publicly available web-based application FUMA (functional mapping and annotation)^84^. We measured the percentage of variance explained in G4 by the *rs62034351* and APOE using linear regression models that included age, age^2^, sex, age-by-sex interaction, batch, array and first five genomic PCs as covariates. Likewise, the variance explained in G6 by *rs11705789* and APOE was measured using linear regression models that included age, age^2^, sex, batch, array and first five genomic PCs as covariates. Model significances were examined by comparing with the model that included all relevant covariates using ANOVA.

### Replication of G&C locus in UKB

For the replication of the G4- and G6-associated locus (SNP with lowest *P* value considered), we used previously published GWAS studies by Sniekers et al.^41^ and Savage et al.^40^. Sniekers and colleagues^41^ performed a genome-wide association meta-analysis on human intelligence using 78,308 European descent individuals from 13 cohorts where phenotype was either Spearman’s *g* or a measure of fluid intelligence. The majority of the study participants (*N* = 54,119) were from UKB. For these participants, only fluid intelligence (either touchscreen or web-based) test score was used, which was considered to correlate highly with *g* (ref. ^85^). We obtained summary statistics for Sniekers et al.^41^ from http://ctg.cncr.nl/software/summary_statistics. The Savage et al.^40^ study performed a genome-wide association meta-analysis in 269,867 European descent individuals from 14 cohorts where various cognitive phenotypes were used to measure intelligence. Most of the study participants (72.5%) were obtained from UKB (*N* = 195,653), for which either touchscreen or web-based fluid intelligence test scores were used. Savage et al.^40^ summary statistics were obtained from https://ctg.cncr.nl/. In both intelligence GWAS studies^40,85^, the imputation of participating cohorts varied. However, the authors provided no details regarding the direction of test scores across participating cohorts. Given that UKB forms the large majority of their participants and fluid intelligence measures were used for UKB-GWAS, we can assume that, overall, a higher score for the phenotype in both studies meant better performance. In contrast, we performed GWAS on G4 and G6, where a higher score meant poor performance. To resolve confusion, we reported replication findings from Sniekers et al.^41^ and Savage et al.^40^, harmonizing the summary statistics in line with the G&C study.

### Functional annotation

FUMA^84^ was used to annotate genome-wide significant loci for G4 and G6. SNP2GENE function in FUMA was used to annotate SNPs and prioritize genes at each locus using gene-based association analysis (implemented in MAGMA^86^) and three gene mapping strategies (positional, eQTL and chromatin interaction). ANNOVAR^87^ implemented in FUMA^84^ annotated SNPs (minimum minor allele frequency threshold set at 0.0001) in LD with independent SNP within a 250 kb window based on the 1000 Genome Phase3 reference panel. SNPs with CADD scores >12.37 are predicted to be pathogenic, RDB scores <2 are predicted to have a regulatory function and chromatin state ≥7 indicates open chromatin region.

### Gene mapping strategies

ANNOVAR^87^-annotated SNPs were used to prioritize genes on the basis of positional, eQTL and chromatin interaction mapping. Positional mapping considered a 10 kb window from the human reference assembly GRCh37/hg19 to map each SNP to genes. For eQTL mapping, SNPs were mapped to eQTL data repositories available by default to annotate SNP effect on gene expression at a false discovery rate threshold <0.05. For chromatin interaction mapping, SNPs were linked to chromatin interaction data available by default to map SNP to gene promoter regions (250 bp upstream and 500 bp downstream of the transcription start site). Also, we opted for annotating enhancer/promoter regions based on Roadmap 111 epigenomes and filtered SNPs overlapping with those regions. A false discovery rate threshold <1 × 10^−6^ was used to detect significant interaction. In addition, we performed GWGBA analysis implemented with MAGMA^86^ to prioritize genes for each genome-wide significant locus where all SNPs from GWAS summary data were mapped to 19,128 protein-coding genes. Genome-wide significance was defined at *P* value of 0.05/19,128 = 2.614 × 10^−6^.

### Tissue specificity and gene expression

Genes prioritized using all mapping strategies (positional, eQTL, chromatin interaction and GWGBA) were used for tissue specificity analysis using the GENE2FUNC option on FUMA^84^. For G4, tissue specificity analysis was performed using predefined differentially expressed gene (DEG) sets for GTEx v8 54 tissue^44^. The gene set was characterized as (1) upregulated DEG, (2) downregulated DEG and (3) DEG, both sides. All FUMA-mapped genes were used as input to test each DEG using default parameters. For G6, bulk tissue gene expression for *GBE1* across GTEx v8 (ref. ^44^) tissues were visualized using GTEx Portal (https://www.gtexportal.org/home/gene/GBE1).

### Gene-set enrichment

FUMA^84^-mapped genes for G4 were used for pathway and process enrichment analysis using ‘Metascape’ (http://metascape.org/)^88^ with input and analysis species set to *Homo sapiens*. Of the 128 genes, Metascape considered 106 genes for the enrichment analysis. The following ontology sources were used in the analysis: KEGG Pathway, GO Molecular Functions, GO Cellular Components, GO Biological Processes, Immunologic Signatures, Oncogenic Signatures, Reactome Gene Sets, Hallmark Gene Sets, Canonical Pathways, Chemical and Genetic Perturbations, BioCarta Gene Sets, CORUM and WikiPathways. We used default Metascape settings. All genes in the genome were used as background for the enrichment in Metascape^88^. Metascape findings were validated using GENE2FUNCTION option on FUMA^84^.

### Colocalization

We examined evidence of shared colocalization between microglia eQTL and G4-associated significant locus at the level of individual genes within a 1 MB window around GWAS-independent SNP. Meta-analyzed (random effects) eQTL summary statistics (out_mfg_stg_svz_tha.metasoft.gz) of four microglial brain regions (medial frontal gyrus, superior temporal gyrus, thalamus and subventricular zone) with random effects were used for colocalization and downloaded from Zenodo (10.5281/zenodo.4118676). We used a Bayesian colocalization method (COLOC^89^) assuming one single causal variant underlying the locus. A total of five hypotheses were tested to evaluate colocalization: H0, there is no causal variant for both traits (PP0); H1 or H2, causal variant associated with either trait 1 or trait 2 (PP1 or PP2); H3, two independent causal variants for trait 1 and trait 2 (PP3); H4, one single causal variant associated with both traits (PP4). COLOC generates a posterior probability (PP) for each hypothesis, with higher values indicating the degree to which we favor a hypothesis. A higher PP for H3 (PP3) supports the presence of two independent variants for both traits. A higher PP for H4 (PP4) supports the presence of single independent variants affecting both traits. We considered thresholds of PP H4 (PP4) ≥0.5 for suggestive, ≥0.7 for moderate and ≥0.8 for strong colocalization, respectively.

### SMR and HEIDI analysis

The SMR method uses principals of Mendelian randomization to integrate summary-level data of an exposure (for example, gene expression) and outcome (that is, intelligence) to test for an association between the two due to a shared and potentially causal variant at a locus^90^. We used SMR to prioritize brain regions and genes associated with G4. We retained 2 Mb regions around GWAS independent SNPs for the analysis where *cis*-eQTLs from 12 GTEx (version 8) brain regions were used as the instrumental variable, gene expression of each brain region as exposure and G4 as the outcome. For each gene, heterogeneity in dependent instruments (HEIDI)^90^ test was performed, which distinguishes pleiotropy (that is, gene expression and G4 are associated owing to a single shared genetic variant) from linkage (that is, two variants in LD independently affecting gene expression and G4). We performed SMR and HEIDI analysis on the Complex-Traits Genetics Virtual Lab^91^ platform. Threshold levels of significance for SMR tests were adjusted for multiple comparisons by Bonferroni correction (*P*_SMR_ < 0.05/number of genes in each eQTL analysis). Genes with *P*_HEIDI_ < 0.05 were considered as linkage and removed.

### Statistical fine mapping

We performed statistical fine mapping of G4- and G6-associated locus. First, GWAS-associated regions were analyzed using GCTA-COJO (v1.94.1)^92^ to identify conditionally independent lead variants. All variants within a 1 MB window of the lead variant were analyzed using FINEMAP (v1.4.2)^93^, a Bayesian fine-mapping method, to identify high-confidence putative causal SNPs for G4 and G6. We allowed for a maximum number of five causal variants for fine mapping. FINEMAP calculates PPs and assigns a Bayes factor to each variant. We considered variants with PP >0.95 and log_10_ Bayes factor ≥2 as plausibly causal.

### Reporting summary

Further information on research design is available in the Nature Portfolio Reporting Summary linked to this article.

## Online content

Any methods, additional references, Nature Portfolio reporting summaries, source data, extended data, supplementary information, acknowledgements, peer review information; details of author contributions and competing interests; and statements of data and code availability are available at 10.1038/s41591-024-02960-5.

### Supplementary information

Supplementary Information Supplementary note, Figs. 1–10 and Tables 1–28.

Reporting Summary

## Data Availability

Summary statistics for G4 and G6 GWAS were deposited in Zenodo at 10.5281/zenodo.10836380 (ref. ^94^). Other data relevant to the study are included in the article or uploaded as online supplementary information. NIHR BioResource holds individual-level genetic and phenotypic data for genes and cognitive study participants that can be accessed through https://bioresource.nihr.ac.uk/using-our-bioresource/.

## Code availability

All software used in this study is publicly available. The codes used for cognitive data cleaning are available on GitHub (https://github.com/shafiqnoa/Genes-and-Cognition-Phase-1/tree/main/Phase1_Cognitive_Data_Clean).
