# Supplementary material for: Dynamics of cognitive variability with age and its genetic underpinning in NIHR BioResource Genes and Cognition cohort participants
Source: Nat Med. 2024 May 14;30(6):1739–48. doi: 10.1038/s41591-024-02960-5 (PMC11186791; doi:10.1038/s41591-024-02960-5)
Supplement: Supplementary file 1 — Supplementary note, Figs. 1–10 and Tables 1–28. [file 41591_2024_2960_MOESM1_ESM.pdf]

nature medicine

Article https://doi.org/10.1038/s41591-024-02960-5

Dynamics of cognitive variability with age and its genetic underpinning in NIHR BioResource Genes and Cognition cohort participants

In the format provided by the
authors and unedited

Contents

Supplementary Note....................2
Supplementary Figures....................5
Supplementary Tables....................14
Reference....................46

Supplementary Note

RT. The test involved 60 questions, which required participants to select the correct pane (left-and right) based on the stimulation provided. The time taken for each question was recorded in milliseconds. This test measures overall alertness and motor speed. The mean reaction time was used as the primary outcome of the test. Prior to analysis, RT scores underwent a log10 transformation.

SB. Each of the participants was shown a box coloured in red, blue, black, or white. Below the box, two names (in black text) were given, one of which described the box colour shown above. Below the names, participants were shown two arrows within the box (left and right) to choose the right colour corresponding to the box. There were 30 questions, each requiring participants to select the right colour displayed in the box as fast and accurately as possible. The test had a pale background and red color was brightened to red orange (a shade that falls between red and orange) to accommodate color blindness, where participants perceive red as dark and mix up red and blue colors. No feedback regarding the correct or incorrect responses was provided. The primary outcome of this test was the average response time, which underwent a log10 transformation. This test was designed to measure processing speed and attention.

SI. Each of the participants was shown a written word describing a color. The word itself was colored in red, blue, black, or white. Below the colored written word, two color names (in black) were provided, one of which correctly described the color of the written word presented above. Below the black-inked color names, the participant was shown two arrows in the box (left and right) to choose the right color indicated above. There were 30 questions requiring the participant to select the right color as fast and accurately as possible. No feedback regarding the correct or incorrect responses was provided. The primary outcome of this test was the average response time, which was log10 transformed. This test was designed to measure processing speed and attention. The test was also designed to accommodate color blindness.

SD. This is also known as symbol digit substitution test. In the test, participants were shown symbols paired with numbers in the top row. Below, in the test row, all of the symbols from the top row were presented in an unorganised manner. Participants were instructed to enter the number that belongs to each symbol as shown in the top row, using the keypad on the screen. Rows were selected at random from the 33 code strings available. The test updated the test row when the participant had completed entering digits for the test row (all 8 codes) and would move onto the next row in the array until the timer of 60 seconds had expired. The test allowed participants to finish their current test row if the timer had expired but did not record any responses after the timeout. The outcome of this test was the total number of correct symbol-digit matches made. Prior to the analysis, we reversed the test scores by subtracting the number of correct matches from the observed maximum number of correct matches seen in the population. This test assesses psychomotor speed.

TMN and TMA. The trail making test consisted of two parts: (i) TMN and (ii) TMA. In the TMN task, participants were shown numbers 1-25 pseudo randomly on the screen and instructed to touch them in numeric order as quickly and accurately as possible. In the TMA task, participants were shown numbers 1-13 and letters A-L together pseudo randomly on the screen and instructed to touch first the number and then letter in numeric and alphabetical order (i.e., 1-A-2-B-3-C) as quickly and accurately as possible. The main outcome for both parts of the tests was mean response time in milliseconds to sequentially identify the correct element of the sequence, which was log10 transformed. Both tests assess executive functioning. In the analysis, we considered both parts of the test separately.

MX. Participants were shown a 3x3 matrix of tiles with one missing piece and provided with eight options at the bottom of the screen to select the missing tile that completed the 3x3 matrix displayed on top. There were 15 questions of this nature, each of which was timed for 60 seconds. If the time for a question expired, the app automatically progressed to the next question and considered the expired question incorrect. The primary outcome of this test was the total number of correct answers made, which was reversed prior to the analysis by using the total number of incorrect answers. This test measures non-verbal fluid intelligence.

WM. This test involved a number shown to participants for memorization, and a proportionate amount of time was given for memorization and recall depending on the length of the number (number of digits). Correct recall of the number resulted in the number string expanding in length (by one digit). All numeric strings of numbers were randomly generated with an algorithm that prevents repeated numbers. Before starting the test, the participants were allowed to have unlimited attempts to practice with single- and double-digit length numbers. The test ended either when the participant made two incorrect recalls of number string consecutively or when the participant reached the end of the test (a 24-digit length string). The primary outcome of this test was the total number of correct responses made by participants, transformed with log10 and reverse-scored in the similar way to SD prior to analysis. The WM test is also known as the numeric memory test.

QZ. This test comprised of 13 questions, which required participants to choose the correct answer from multiple choices (5 options, with one exception having 4 options). The test had a fixed duration of 2 minutes. After that, participants were not allowed to complete the rest of the test. The time taken to answer each question was recorded in milliseconds. The primary outcome of this test was the total number of correct responses made within 2 minutes. Prior to the analysis, test scores were reversed by using the total number of incorrect answers. This test is also recognized as a measure of fluid intelligence, which measures verbal and numerical reasoning abilities.

VY. This test involved answering 20 questions (stimuli provided in Supplementary Table 28). For each question, a word was shown on the left side of the screen and participants were provided with 6 words on the right side of the screen to choose the correct synonym. The time limit for each question was 21 seconds.

The primary outcome of this test was the total number of correct answers made, which was reversed by using the total number of incorrect answers. This test assesses crystallized intelligence.

PR. This test involved participants recalling the positions of different shapes. First, participants were presented with 7 different shapes in 7 different locations, one after another. Once all shapes were displayed, one of the shapes was shown in the middle of the screen and participants were invited to identify its correct location based on its previous appearance. The primary measure for this test was the total number of guesses required to complete the task which underwent a log10 transformation. This test is designed to measure episodic memory and attention.

G4 and G6. Initially, we performed PC analysis using all cognitive test scores. Eigenvalues depicted on the scree plot indicated three PCs where 6 tests (RT, TMN, TMA, SB, SI and SD) fall onto the 1st PC (explaining 41.3% variance), 4 tests (WM, MX, QZ and VY) fall onto the 2nd PC (explaining 15.6% of the variance) and PR falls onto the 3rd PC. Based on the initial observations, we decided to create two measures of general cognitive ability. We used scores of RT, TMN, TMA, SB, SI and SD tests to create PCs. Scores on the first rotated PC, which accounted for 66.5% of the total variance (Extended Data Fig 3A and C), were used as a measure of general cognitive ability (we named it *G6*). The expected contribution of TMN, TMA and RT to G6 was lower than SB, SI and SD (Extended Data Fig 3B). Likewise, we used WM, MX, QZ and VY to create PCs and the first rotated PC, which accounted for 46.6% of the total variance (Extended Data Fig 4A and C), were used as the 2nd measure of general cognitive ability (we named it *G4*). The expected contribution of MX and WM to G4 was lower (Extended Data Fig 4B). We used the "prcomp" function from *the stats* package in *R* for PCA analysis. Note, PR was not included in the calculation of general cognitive ability.

Supplementary Figures

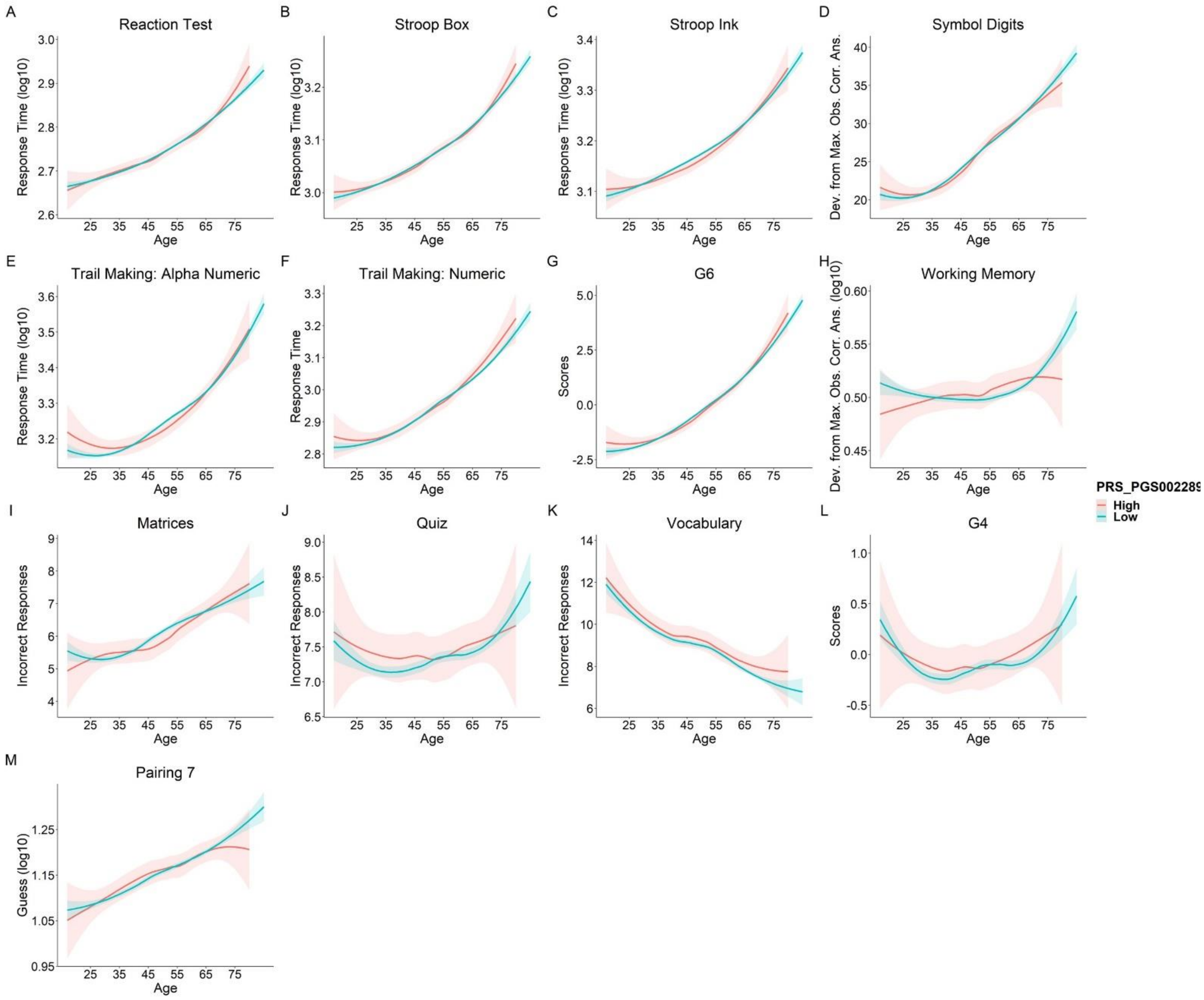

Supplementary Fig. 1 | 11 Cognitive tests and two measures of general cognitive ability (G4 and G6) stratified by Alzheimer's disease polygenic risk score (AD-PRS) groups (derived from the polygenic score catalogue ID: PGS002289, excluded APOE region). The x-axis represents the age, and the y-axis represents scores for the corresponding test (A-K) or measures of general cognitive ability (L-M). Red and blue lines across the plots represent lines of best fit with standard error for AD risk groups (High and Low), specified on the right side of the plot with a color-coded legend. Response Time is the average time taken per item.

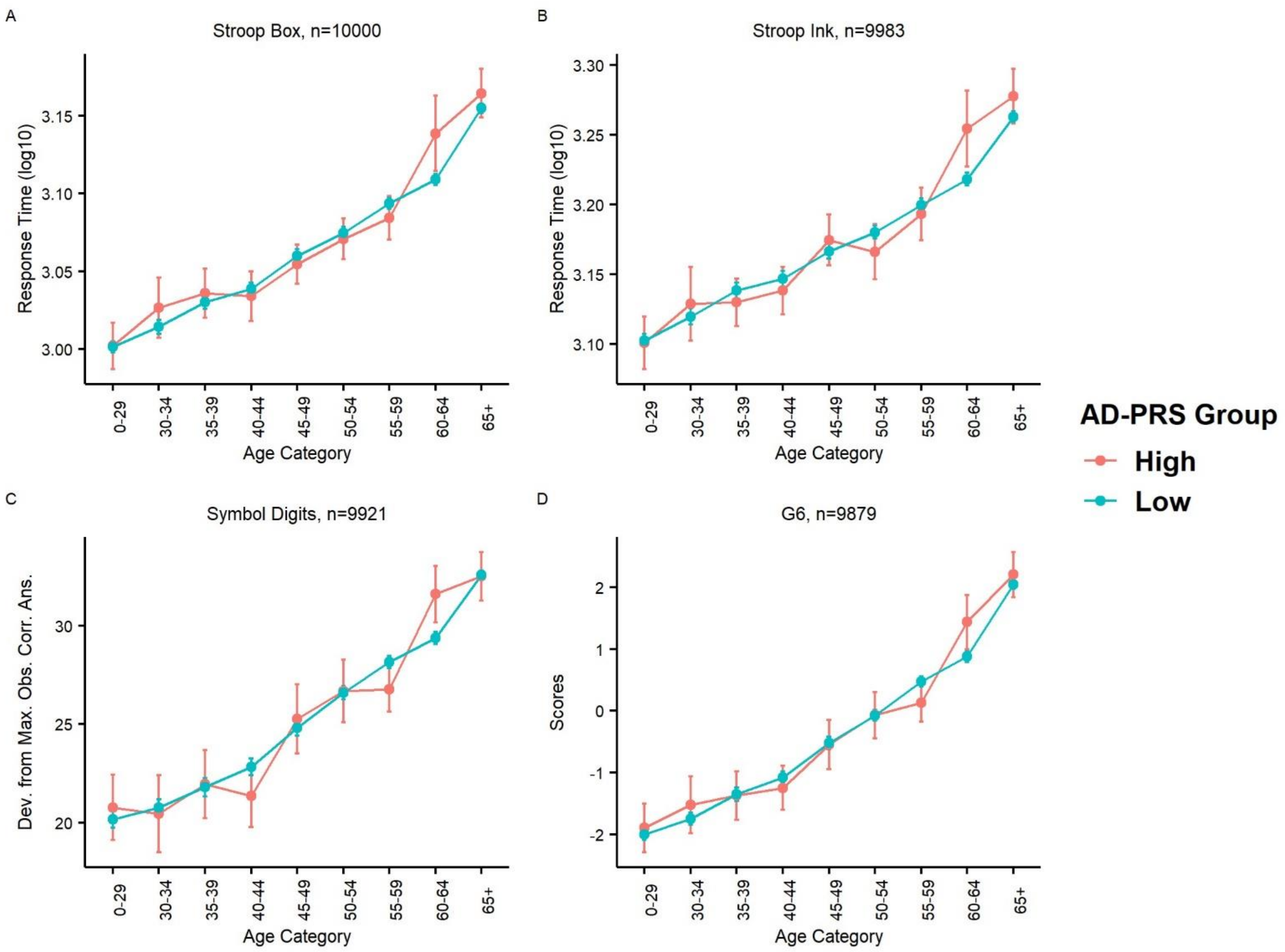

Supplementary Fig. 2 | Mean cognitive phenotype scores across age categories stratified by Alzheimer's disease polygenic risk score (AD-PRS) group (derived from the polygenic score catalogue ID: PGS002289, included APOE region). (A) Stroop box, (B) Stroop Ink, (C) Symbol Digits and (D) G6. (A-D) In each of the plots, mean score differences between AD-PRS groups across different age groups were assessed using t-tests. The x-axis represents age categories and y-axis indicate scores for the corresponding cognitive phenotype. Bars are aligned based on age category and indicates 95% confidence interval for mean. Red and blue lines across plots represent AD risk groups (High and Low), specified in the right side of the plot with colour coded legend. Response Time is the average time taken per item.

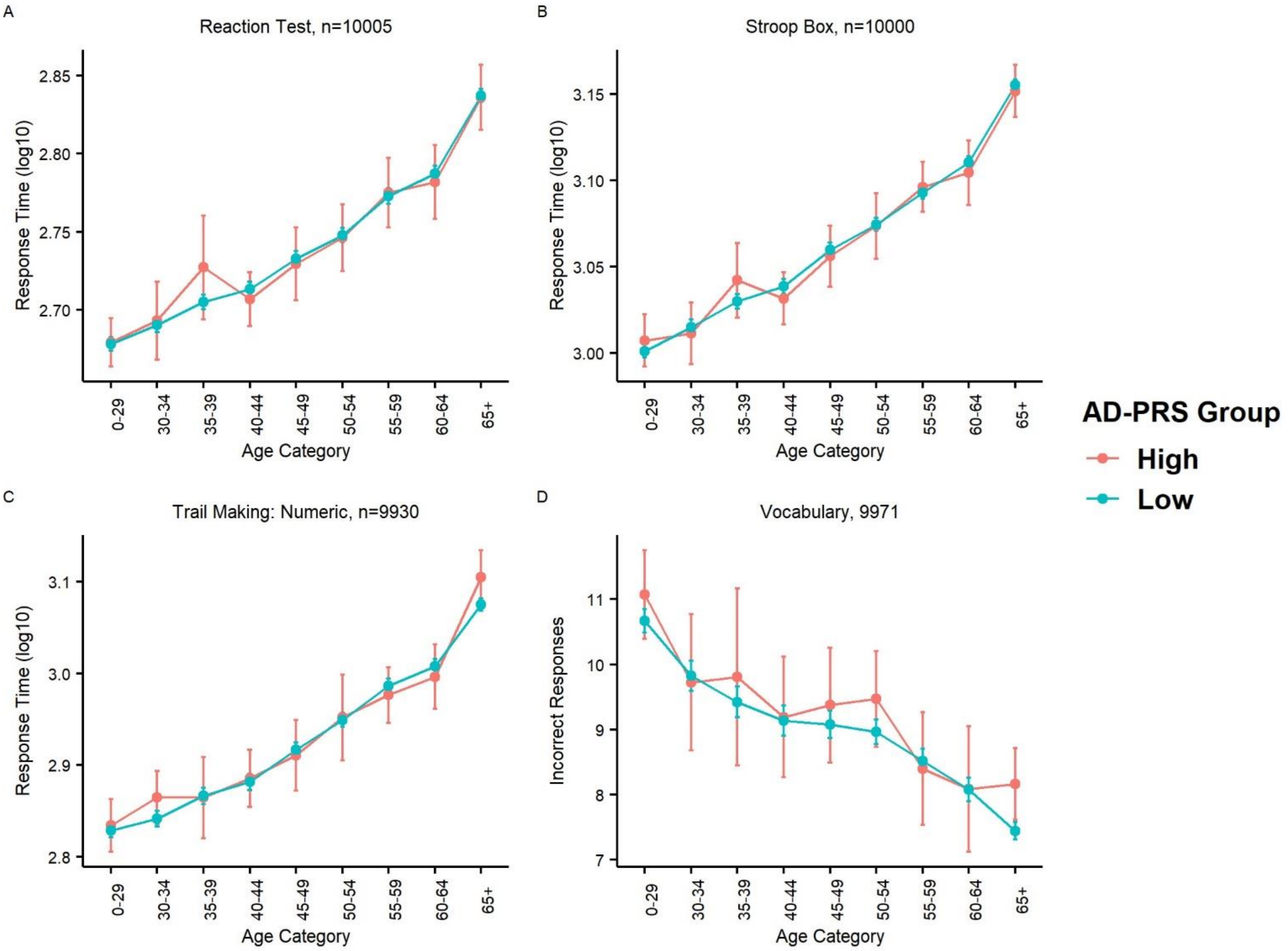

Supplementary Fig. 3 | Mean cognitive phenotype scores across age categories stratified by Alzheimer's disease polygenic risk score (AD-PRS) group (derived from the polygenic score catalogue ID: PGS002289, excluded APOE region). (A) Reaction test, (B) Stroop Box, (C) Trail Making: Numeric and (D) Vocabulary. (A-D) In each of the plots, mean score differences between AD-PRS groups across different age groups were assessed using t-tests. The x-axis represents age categories and y-axis indicate scores for the corresponding cognitive phenotype. Bars are aligned based on age category and indicates 95% confidence interval for mean. Red and blue lines across plots represent AD risk groups (High and Low), specified in the right side of the plot with colour coded legend. Response Time is the average time taken per item.

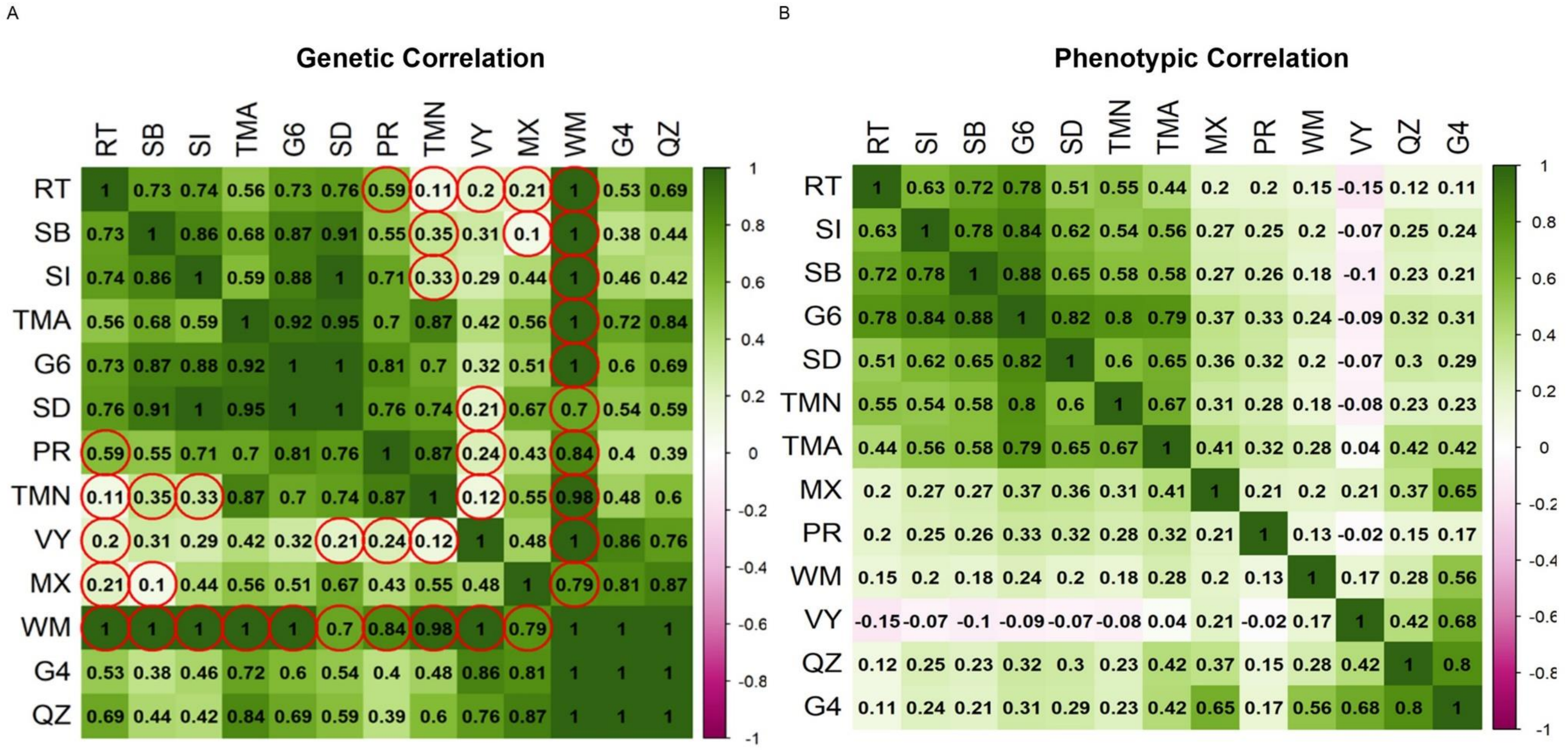

Supplementary Fig. 4 | (A) Heatmap of genetic correlation for 11 cognitive tests and two measures of general cognitive ability (G4 and G6). Cells highlighted in red circle indicate non-significant correlation (two-tailed p>0.05), and **(B) Heatmap of phenotypic correlation for 11 cognitive tests and two measures of general cognitive ability (G4 and G6). (A-B)** QZ: Quiz; WM: Working memory; MX: Matrices; SD: Symbols Digit; VY: Vocabulary; RT: Reaction Test; PR: Pairing 7; SB: Stroop Box; SI: Stroop Ink; TMN: Trail Making Numeric; TMA: Trail Making Alpha Numeric. Phenotypic and genetic correlation analyses was confined to participants for whom genetic data was available and completed all cognitive tests. In both plots, each coloured cell indicates magnitudes of correlations. Each coloured cell indicates magnitudes of correlations. The corresponding colour scale is presented on the right side of the heatmap where dark green represents the highest correlation, and darker moderate pink represents highest negative correlation. On the x- and y-axis, all cognitive phenotypes are presented maintaining the order.

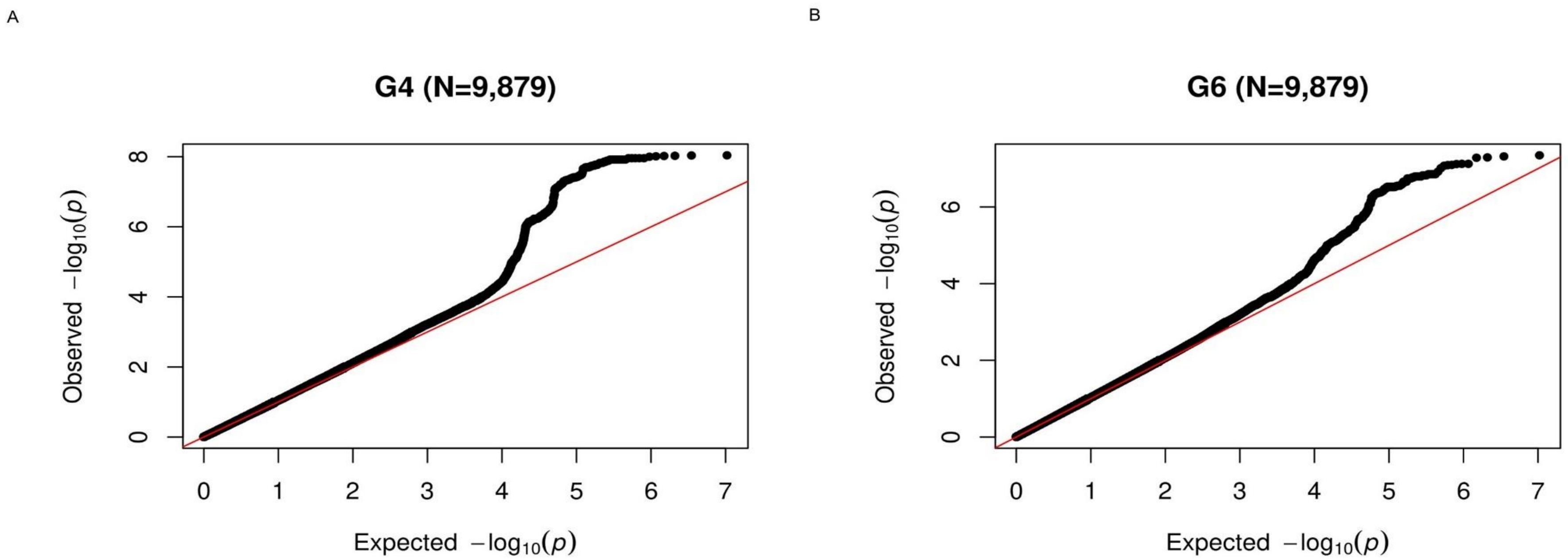

Supplementary Fig. 5 | The QQ plot of GWAS summary statistics of G4 **(A)** and G6 **(B)**. **(A-B)** The x-axis shows the expected – log10 transformed p-values and the y-axis shows the observed –log10 transformed p-values from the two-sided BOLT infinitesimal model.

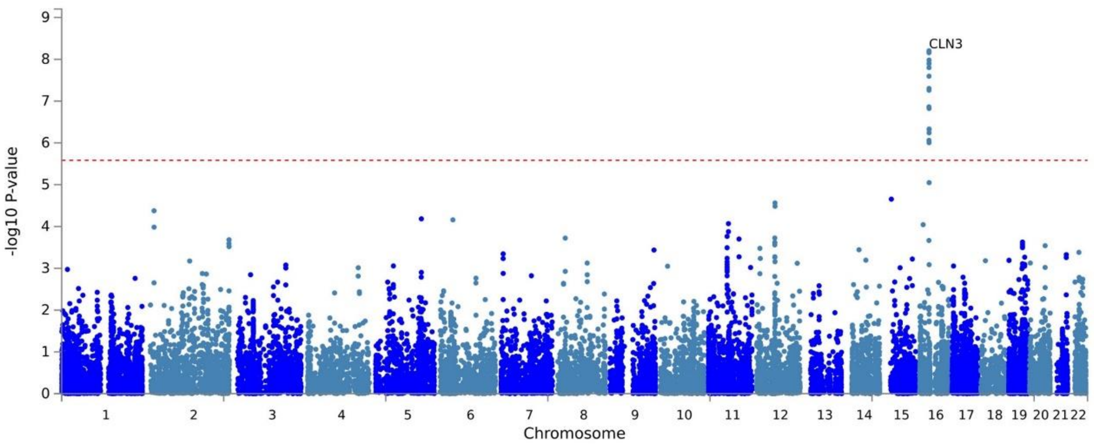

Supplementary Fig. 6 | Manhattan plot for the genome-wide gene-based association analysis of G4. The y-axis shows the ─log10 transformed two-tailed p-value of each gene from a linear model and the chromosomal position on the x-axis. The red dotted line indicates the Bonferroni-corrected threshold for the genome-wide significance of the gene-based test. The gene with the lowest p-value was highlighted.

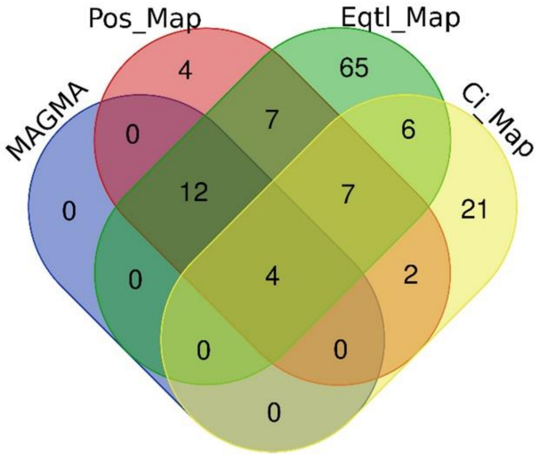

Supplementary Fig. 7 | Venn diagram showing overlap of prioritised genes for G4 implicated by genome wide gene-based analysis (implemented in MAGMA), positional mapping (Pos_Map), chromatin interaction mapping (Ci_Map), and expression quantitative trait locus mapping (Eqtl_Map).

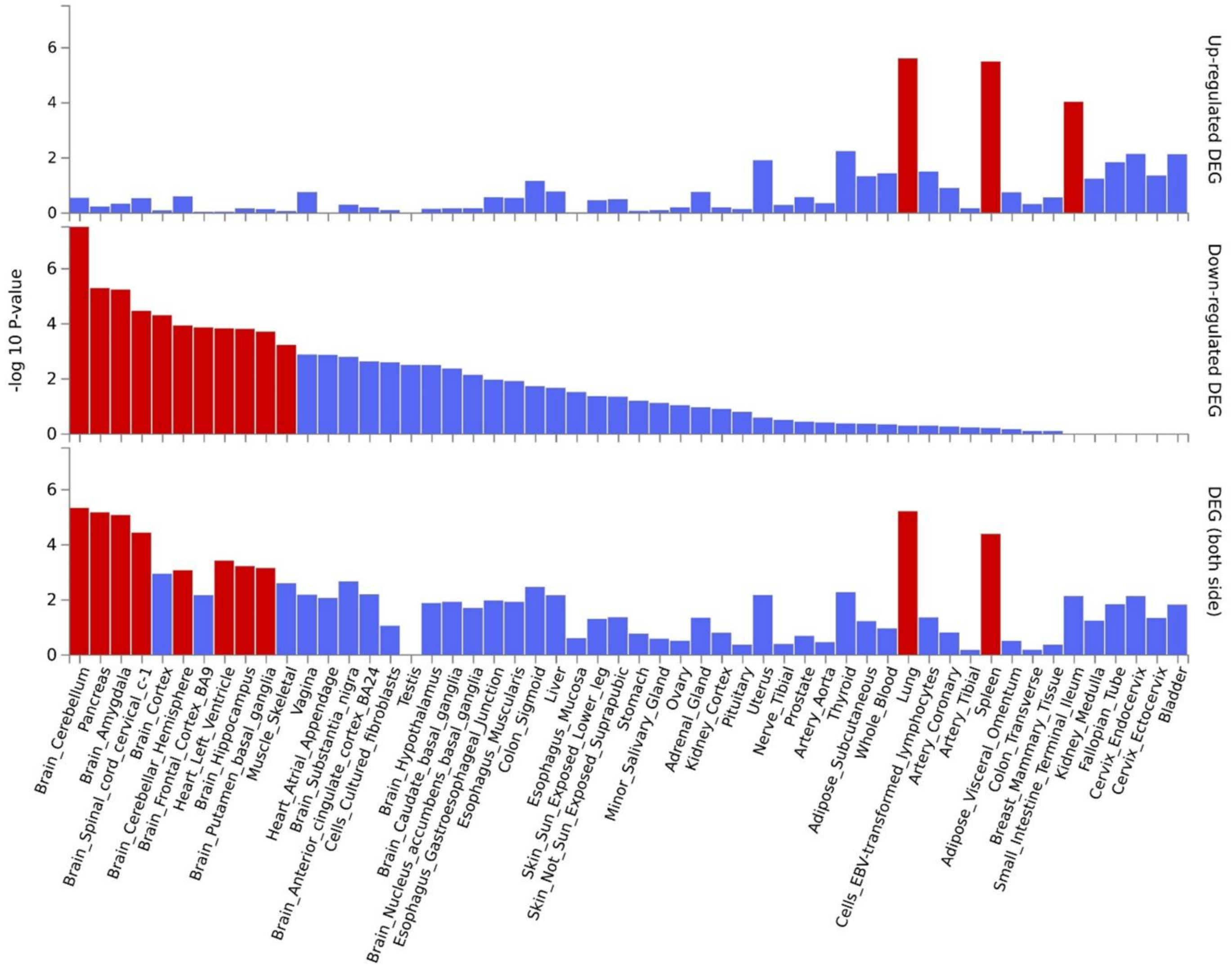

Supplementary Fig. 8 | Differentially expressed gene (DEG) plots for G4 in 54 tissue types from GTEX v8. The x-axis represents the GTEx (v8) tissues, and the y-axis represents the −log10 transformed two-tailed p-value of the hypergeometric test. Significantly enriched DEG sets (Bonferroni-corrected p value <0.05) are highlighted in red.

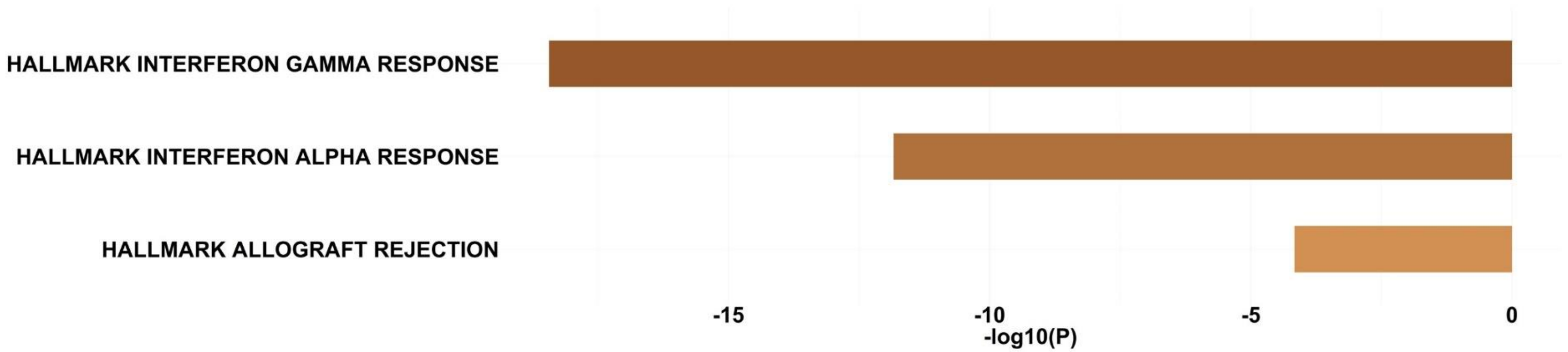

Supplementary Fig. 9 | Enrichment for hallmark gene-sets for G4. Two-tailed p-values (-log10 transformed) are computed using the cumulative hypergeometric distribution.

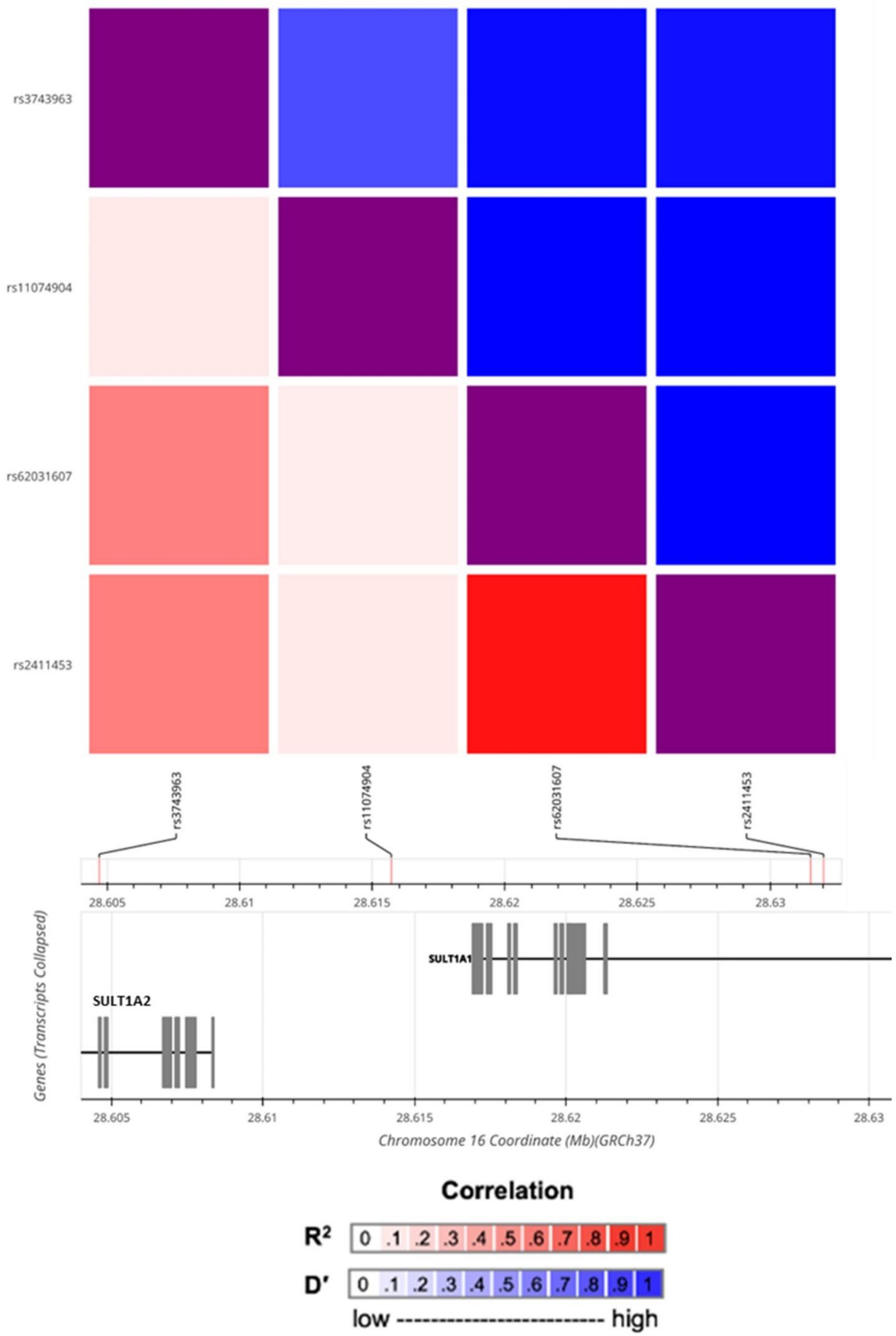

Supplementary Fig. 10 | Fine mapped SNPs for G4 and their intercorrelation. The strength of correlation is presented at the bottom of the plot. This plot was created using the LDmatrix tool (https://ldlink.nih.gov/?tab=ldmatrix)[95].

Supplementary Tables

Supplementary Table 1 | Self-reported clinical information available on G&C study participants.

Diagnosis [1]	N=21,051
Colour Blindness, yes; n (%)	605 (2.9)
Learning Disability, yes; n (%)	720 (3.5)
Brain Surgery (affect day to day life), n	1
Charcot-Marie-Tooth, n	1
Mild Cognitive Impairment, n	1
Memory Issue, n	1
Myopic Maculopathy, n	1
Osteoarthritis (thumbs and wrist), n	1
Arthritis, yes; n (%)	1155 (5.5)
Diabetes, yes; n (%)	561 (2.7)
Heart Conditions, yes; n (%)	368 (1.7)
High Blood Pressure, yes; n (%)	1844 (8.8)
Mental Health Disorders, yes; n (%)	192 (0.9)
Stroke and Associated Conditions, yes; n (%)	110 (0.5)
ADHD or Autism, yes; n (%)	7 (0.0)

[1] All diagnoses were self-reported in response to questionnaire provided either by NIHR BioResource or Cognitive Test application.

Supplementary Table 2 | Types of transformation performed to cognitive test scores, what domain of cognition these tests cover and what the score means.

Tests	Transformation	Intelligence Type/Domain	Scores Meaning
Reaction Test (RT)	Log10	Reaction time	A higher score means poorer performance[1]
Stroop Box (SB)	Log10	Attention, processing speed	
Stroop Ink (SI)	Log10	Attention, processing speed	
Trail Making: Numeric (TMN)	Log10	Executive functioning	
Trail Making: Alpha Numeric (TMA)	Log10	Executive functioning	
Symbols Digit (SD)	None	Psychomotor speed (processing speed)	
Matrices (MX)	None	Non-verbal reasoning (non-verbal fluid intelligence)	
Working memory (WM)	Log10	Short term memory, attention, executive function	
Quiz (QZ)	None	Fluid intelligence (verbal and numeric reasoning)	
Vocabulary (VY)	None	Vocabulary level (Crystalized intelligence)	
Pairing 7 (PR)	Log10	Visual episodic memory	

[1] *SD, MX, WM, QZ, and VY* scores were reversed (following the transformation for WM) using formula: max (test score)-tests score to have same direction of interpretation as other tests.

Supplementary Table 3 | Median and mean values for different cognitive test scores for all participants and for those whose genetic data were available.

	All samples (N=20,928)			Genetic data Available (N=10,038)		
Tests[1]	Max-Min	Median [IQR[1]]	Mean (S.Dev[1])	Max-Min	Median [IQR[1]]	Mean (S.Dev[1])
RT	3.56-2.40	2.73 [2.69, 2.80]	2.75 (0.10)	3.56-2.40	2.74 [2.69, 2.81]	2.76 (0.10)
SB	3.57-2.83	3.07 [3.02, 3.13]	3.08 (0.08)	3.57-2.85	3.07 [3.02, 3.13]	3.08 (0.08)
SI	3.75-2.83	3.18 [3.12, 3.24]	3.18 (0.10)	3.75-2.83	3.18 [3.12, 3.25]	3.19 (0.10)
SD	54-0	27.00 [22.00, 31.00]	26.44 (7.09)	54-2	27.00 [22.00, 32.00]	26.73 (7.15)
TMN	4.02-2.50	2.94 [2.84, 3.04]	2.95 (0.16)	4.02-2.50	2.95 [2.85, 3.05]	2.96 (0.16)
TMA	4.15-2.73	3.25 [3.15, 3.36]	3.26 (0.16)	4.15-2.78	3.25 [3.15, 3.36]	3.26 (0.16)
MX	14-0	5.00 [4.00, 7.00]	5.22 (2.26)	14-0	5.00 [4.00, 7.00]	5.21 (2.25)
WM	1.10-0	0.49 [0.44, 0.55]	0.51 (0.09)	1.10-0	0.49 [0.44, 0.55]	0.50 (0.08)
QZ	13-0	8.00 [6.00, 9.00]	7.51 (2.11)	12-0	7.00 [6.00, 9.00]	7.34 (2.12)
VY	20-0	9.00 [7.00, 11.00]	9.12 (3.29)	10-0	9.00 [6.00, 11.00]	8.73 (3.31)
PR	1.69-0.85	1.18 [1.04, 1.28]	1.16 (0.16)	1.64-0.85	1.18 [1.04, 1.28]	1.16 (0.16)

[1] RT: Reaction Test; SB: Stroop Box; SI: Stroop Ink; SD: Symbols Digit; TMN: Trail Making Numeric; TMA: Trail Making Alpha Numeric; MX: Matrices; WM: Working memory; QZ: Quiz; VY: Vocabulary; PR: Pairing7; IQR: interquartile range; and S.Dev: Standard Deviation.

Supplementary Table 4 | The association between cognitive phenotypes (including measures of general cognitive ability) and device used to take the cognitive tests.

		Unadjusted				Adjusted [c]			
Phenotypes [a]	Devices [b]	Beta	SE [a]	P [a]	R-square [a]	Beta	SE [a]	P [a]	R-square [a]
RT	Android	0.0336	0.0012	<0.0001	0.4035	0.0419	0.0010	<0.0001	0.5605
	Windows	0.1581	0.0013	<0.0001		0.1338	0.0012	<0.0001	
SB	Android	0.0012	0.0012	0.3032	0.1487	0.0102	0.0010	<0.0001	0.4303
	Windows	0.0751	0.0013	<0.0001		0.0466	0.0011	<0.0001	
SI	Android	-0.0030	0.0015	0.0383	0.0996	0.0062	0.0013	<0.0001	0.3195
	Windows	0.0714	0.0016	<0.0001		0.0413	0.0015	<0.0001	
SD	Android	-0.9170	0.1111	<0.0001	0.0577	-0.1891	0.0929	0.0419	0.3460
	Windows	3.6016	0.1230	<0.0001		0.9597	0.1066	<0.0001	
TMN	Android	-0.0134	0.0024	<0.0001	0.0812	0.0003	0.0021	0.8857	0.3006
	Windows	0.1013	0.0027	<0.0001		0.0491	0.0024	<0.0001	
TMA	Android	-0.0211	0.0026	<0.0001	0.0191	-0.0069	0.0023	0.0025	0.3006
	Windows	0.0407	0.0029	<0.0001		-0.0115	0.0026	<0.0001	
MX	Android	-0.1644	0.0364	<0.0001	0.0012	-0.0188	0.0349	0.5897	0.0862
	Windows	-0.1668	0.0403	<0.0001		-0.5108	0.0401	<0.0001	
WM	Android	-0.0012	0.0014	0.3928	0.0002	-0.0002	0.0014	0.8740	0.0035
	Windows	0.0028	0.0015	0.0646		0.0013	0.0016	0.4212	
QZ	Android	-0.1530	0.0339	<0.0001	0.0028	-0.1150	0.0339	0.0007	0.0098
	Windows	-0.2830	0.0375	<0.0001		-0.3351	0.0388	<0.0001	
VY	Android	-0.0185	0.0519	0.7219	0.0353	-0.1639	0.0501	0.0011	0.1082
	Windows	-1.4846	0.0575	<0.0001		-0.8346	0.0575	<0.0001	
PR	Android	-0.0097	0.0026	0.0002	0.0041	-0.0012	0.0026	0.6397	0.0756
	Windows	0.0196	0.0029	<0.0001		-0.0115	0.0029	0.0001	
G6	Android	0.0017	0.0297	0.9540	0.1593	0.2255	0.0230	<0.0001	0.5007
	Windows	1.9057	0.0327	<0.0001		1.1145	0.0262	<0.0001	
G4	Android	-0.0785	0.0220	0.0004	0.0088	-0.0551	0.0220	0.0122	0.0208
	Windows	-0.3287	0.0243	<0.0001		-0.3242	0.0251	<0.0001	

[a] RT: Reaction Test; SB: Stroop Box; SI: Stroop Ink; SD: Symbols Digit; TMN: Trail Making Numeric; TMA: Trail Making Alpha Numeric; MX: Matrices; WM: Working memory; QZ: Quiz; VY: Vocabulary; PR: Pairing7; G6: a summary measure computed using RT, SB, SI, SD, TMN and TMA; G4: a summary measure computed using WM, MX, QZ and VY; SE: Standard Error; P: two-tailed p-value and R-square (R^2): variance explained by the linear regression model.

[b] Reference Category for Devices: iOS users.

[c] Model adjusted for age and gender.

Supplementary Table 5 | The differences in age, deprivation, and education among device users.

	Android (n=6572)	iOS (n=9599)	Windows (n=4757)	P[b]
**Age (yrs),** Mean (S.Dev[a])	46.3 (13.7)	49.2 (14.7)	58.7 (13.1)	<0.0001[c]
**Age (yrs),** Median (IQR[a])	46 (22)	51 (23)	62 (15)	
**Multiple Deprivation Index**, n (%)				
High (1-3)	1472 (0.7)	1393 (0.7)	586 (0.3)	<0.0001[d]
Medium (4-7)	2635 (0.13)	3770 (0.19)	1878 (0.09)	
Low (8-10)	2115 (0.11)	4049 (0.20)	2147 (0.11)	
**Education, n (%)**				
1 (lowest)	84 (0.01)	134 (0.02)	73 (0.01)	0.024 [d]
2	609 (0.08)	909 (0.12)	490 (0.07)	
3	192 (0.03)	331 (0.04)	221 (0.03)	
4 (highest)	1370 (0.18)	1884 (0.25)	1145 (0.15)	

[a]S.Dev: Standard Deviation and IQR: Interquartile Range
[b]P: Two-tailed p-value.
[c]P-value obtained using one-way ANOVA test.
[d]P-value obtained using Chi-squared test.

Supplementary Table 6 | Study characteristics of participants with genetically inferred European ancestry.

**Characteristics**	**N=10,038**	**Missing (%)**
**Age[a]**, Mean (S.Dev[b]) / Median (IQR[b])	51.91 (14.70) / 54 (41, 64)	-
**Age category;** n (%)		
17-25	464 (4.6)	-
26-35	1269 (12.6)	-
36-45	1559 (15.5)	-
46-55	2150 (21.4)	-
56-65	2527 (25.2)	-
66-75	1848 (18.4)	-
76+	221 (2.2)	-
**Sex,** Female/Male; n (%)	6474 (64.5) / 3565 (35.5)	
**Ethnicity[a],** n (%)		
African	1 (0.0)	
Mixed	20 (0.2)	
Other	28 (0.3)	2.2
White	9763 (99.5)	
**Smoking status[a],** n (%)		
Current Smoker	251 (4.5)	44.5
Non-Smoker	3298 (59.2)	
Past Smoker	2024 (36.3)	
**Alcohol use[a] (yes),** n (%)	5010 (86.8)	42.5
**Body Mass Index[a],** n (%)		
Underweight	68 (1.2)	
Healthy weight	2523 (44.2)	43.1
Overweight	2054 (36.0)	
Obese	1063 (18.6)	
**Multiple Deprivation index,** n (%)		
High (1-3)	1593 (16.4)	
Medium (4-7)	3998 (41.3)	3.5
Low (8-10)	4095 (42.3)	
**Education[a],** n (%)		
1 (lowest)	212 (3.9)	
2	1416 (26.3)	46.5
3	548 (10.2)	
4 (highest)	3199 (59.5)	
**Worked nights 72 hours before test[a] (yes),** n (%)	166 (1.7)	-
**First language is English[a],** n (%)	9656 (97.4)	1.3

[a]Self-reported in response to questionnaire provided either by NIHR BioResource or Cognitive Test application.
[b]S.Dev: Standard Deviation and IQR: Interquartile Range

Supplementary Table 7 | Self-reported clinical information available on G&C study participants with genetically inferred European ancestry.

Diagnosis [1]	N=10,038
Colour Blindness, yes; n (%)	284 (2.8)
Learning Disability, yes; n (%)	297 (3.0)
Arthritis, yes; n (%)	617 (6.1)
Diabetes, yes; n (%)	313 (3.1)
Heart Conditions, yes; n (%)	201 (2.0)
High Blood Pressure, yes; n (%)	1,082 (10.8)
Mental Health Disorders, yes; n (%)	83 (0.8)

[1] All diagnoses were self-reported in response to questionnaire provided either by Bioresource or Cognitive Test application.

Supplementary Table 8 | The association between cognitive phenotypes (including measures of general cognitive ability), age, and sex.

Phenotypes [a]	Covariates	Model-1 [c]				Model-2 [c]			
		Beta	SE [a]	P [a, d]	R-square [a]	Beta	SE	P [a, d]	R-square [a]
RT	Age	0.0029	0.00004	< 0.0001	0.5625	0.0029	0.00005	< 0.0001	0.5647
	Age^2	0.00002	0.000003	< 0.0001		0.00002	0.00000	< 0.0001	
	Gender [b]	-0.0172	0.0013	< 0.0001		-0.0168	0.0014	< 0.0001	
	Age* Gender	0.0001	0.0001	0.0731		0.0001	0.00007	0.0634	
	Age^2* Gender	0.00001	0.000004	0.0158		0.000009	0.00000	0.0702	
SB	Age	0.0033	0.00004	< 0.0001	0.4383	0.0033	0.00004	< 0.0001	0.4379
	Age^2	0.00003	0.000002	< 0.0001		0.00003	0.00000	< 0.0001	
	Gender [b]	-0.0067	0.0009	< 0.0001		-0.0066	0.0009	< 0.0001	
	Age* Gender	-0.0002	0.00006	0.0049		-0.0002	0.00007	0.0632	
SI	Age	0.0034	0.00004	< 0.0001	0.3248	0.0034	0.00004	< 0.0001	0.3289
	Age^2	0.00003	0.000003	< 0.0001		0.00003	0.00000	< 0.0001	
	Gender [b]	-0.0075	0.0016	< 0.0001		-0.0069	0.0017	< 0.0001	
	Age^2* Gender	0.000009	0.000005	0.0853		0.000008	0.00000	0.12	
SD	Age	0.289	0.0038	< 0.0001	0.354	0.295	0.0040	< 0.0001	0.3625
	Age^2	0.0033	0.0002	< 0.0001		0.0032	0.0002	< 0.0001	
	Gender [b]	0.61	0.116	< 0.0001		0.6	0.12	< 0.0001	
	Age*Gender	-0.0176	0.006	0.0094		-0.0144	0.0062	0.0632	
	Age^2*Gender	-0.0010	0.0004	0.0158		-0.0009	0.0004	0.0468	
TMN	Age	0.0054	0.00007	< 0.0001	0.305	0.0055	0.00007	< 0.0001	0.3067
	Age^2	0.00005	0.000004	< 0.0001		0.00005	0.00000	< 0.0001	
	Gender [b]	0.0158	0.0020	< 0.0001		0.0168	0.0020	< 0.0001	
TMA	Age	0.0056	0.00007	< 0.0001	0.2462	0.0058	0.00008	< 0.0001	0.2545
	Age^2	0.00009	0.000004	< 0.0001		0.00009	0.00000	< 0.0001	
	Gender [b]	0.0093	0.0021	< 0.0001		0.01	0.0022	< 0.0001	
WM	Age	0.0004	0.00004	< 0.0001	0.0109	0.0005	0.00005	< 0.0001	0.0131
	Age^2	0.00003	0.000003	< 0.0001		0.00003	0.00000	< 0.0001	
	Gender [b]	-0.0076	0.0013	< 0.0001		-0.0073	0.0013	< 0.0001	
MX	Age	0.0487	0.0011	< 0.0001	0.0882	0.0513	0.0012	< 0.0001	0.0995
	Age^2	0.0005	0.00007	< 0.0001		0.0004	0.00007	< 0.0001	
	Gender [b]	-0.558	0.0321	< 0.0001		-0.545	0.0332	< 0.0001	
QZ	Age	0.0106	0.0014	< 0.0001	0.0116	0.0125	0.0014	< 0.0001	0.0180
	Age^2	0.0003	0.00007	< 0.0001		0.0004	0.00007	< 0.0001	
	Gender [b]	-0.283	0.0313	< 0.0001		-0.288	0.0327	< 0.0001	
	Age*Gender	0.0050	0.0022	0.0401		0.0050	0.0023	0.0632	
VY	Age	-0.0638	0.0019	< 0.0001	0.1093	-0.0615	0.0020	< 0.0001	0.112
	Sex [b]	-0.544	0.0463	< 0.0001		-0.54	0.0484	< 0.0001	
	Age*Gender	0.0174	0.0031	< 0.0001		0.0185	0.0033	< 0.0001	
PR	Age	0.0031	0.00008	< 0.0001	0.0773	0.00312	0.00008	< 0.0001	0.0772
	Gender [b]	0.0099	0.0024	0.0001		0.01	0.0024	< 0.0001	
G6	Age	0.0872	0.0007	< 0.0001	0.5104	0.0888	0.0008	< 0.0001	0.5165
	Age^2	0.0009	0.00004	< 0.0001		0.0009	0.00005	< 0.0001	
	Gender [b]	-0.0388	0.0208	0.0631		-0.0298	0.0216	0.17	
G4	Age	0.0054	0.0009	< 0.0001	0.0253	0.00689	0.00091	< 0.0001	0.0368
	Age^2	0.0004	0.00004	< 0.0001		0.0004	0.00004	< 0.0001	
	Gender [b]	-0.316	0.0203	< 0.0001		-0.314	0.021	< 0.0001	
	Age*Gender	0.0048	0.0014	0.0035		0.0052	0.0015	0.0018	

[a] RT: Reaction Test; SB: Stroop Box; SI: Stroop Ink; SD: Symbols Digit; TMN: Trail Making Numeric; TMA: Trail Making Alpha Numeric; MX: Matrices; WM: Working memory; QZ: Quiz; VY: Vocabulary; PR: Pairing7; G6: a summary measure computed using RT, SB, SI, SD, TMN and TMA; G4: a summary measure computed using WM, MX, QZ and VY; SE: Standard Error; P: two-tailed p-value and R-square (R^2): adjusted R^2 obtained from respective linear regression model.
[b] Reference Category for gender: Female.
[c] Model-1 was adjusted for device use, except for working memory test. Model-2 were adjusted for Model-1 + Deprivation Index (as categorical variable) + Self-reported Ethnicity.
[d] P-values were presented after considering Bonferroni-Holm adjustment, considering the number of cognitive phenotypes each covariate was tested against.

Supplementary Table 9 | The association between cognitive phenotypes (including measures of general cognitive ability) and education.

Phenotypes [a]	Education						
	1 (lowest) [b]		2nd Highest [b]		3rd Highest [b]		
	B (SE) [a]	P [a, c]	B (SE) [a]	P [a, c]	B (SE) [a]	P [a, c]	P-trend [a, c]
RT [d]	0.0167	<0.0001	0.005 (0.0018)	0.0097	0.0023 (0.0026)	0.75	<0.0001
SB [d]	0.0232	<0.0001	0.0072 (0.0017)	<0.0001	0.005 (0.0025)	0.15	<0.0001
SI [d]	0.0299	<0.0001	0.0048 (0.0021)	0.0236	0.0072 (0.0032)	0.12	<0.0001
SD	3.47 (0.341)	<0.0001	1.10 (0.150)	<0.0001	0.868 (0.224)	0.001	<0.0001
TMN [d]	0.0522	<0.0001	0.0134 (0.0037)	0.00095	0.0044 (0.0054)	0.75	<0.0001
TMA [d]	0.0947	<0.0001	0.0265 (0.0038)	<0.0001	0.0133 (0.0057)	0.11	<0.0001
WM [d]	0.0396	<0.0001	0.0116 (0.0023)	<0.0001	0.0087 (0.0034)	0.073	<0.0001
MX	2.18 (0.128)	<0.0001	0.858 (0.0564)	<0.0001	0.673 (0.084)	<0.0001	<0.0001
QZ	2.19 (0.125)	<0.0001	0.965 (0.0551)	<0.0001	0.689 (0.082)	<0.0001	<0.0001
VY	4.44 (0.180)	<0.0001	2.47 (0.0796)	<0.0001	1.89 (0.118)	<0.0001	<0.0001
PR [d]	0.0343 (0.1)	0.0004	0.0142 (0.0042)	0.0023	0.0125 (0.0063)	0.15	<0.0001
G6	0.876 (0.086)	<0.0001	0.245 (0.0379)	<0.0001	0.160 (0.0563)	0.037	<0.0001
G4	1.94 (0.0781)	<0.0001	0.886 (0.0344)	<0.0001	0.670 (0.0511)	<0.0001	<0.0001

[a] RT: Reaction Test; SB: Stroop Box; SI: Stroop Ink; SD: Symbols Digit; TMN: Trail Making Numeric; TMA: Trail Making Alpha Numeric; MX: Matrices; WM: Working memory; QZ: Quiz; VY: Vocabulary; PR: Pairing7; G6: a summary measure computed using RT, SB, SI, SD, TMN and TMA; G4: a summary measure computed using WM, MX, QZ and VY; B: Beta; SE: Standard Error; P: two-tailed p-value; P-trend: two-tailed p-value for trend.

[b] Reference group: Highest education; Linear regression models were commonly adjusted for age, age-square (except VY and PR), gender and types of devices used to take the test. An age by gender interaction term/s was included in the model for RT, SB, SI, SD, QZ, VY and G4.

[c] P-values were presented after Bonferroni Holm adjustment for 13 tests.

[d] Log10 transformation was performed for these test scores.

Supplementary Table 10 | The association between cognitive phenotypes (including measures of general cognitive ability) and multiple deprivation.

Phenotypes [a]	Multiple Deprivation				
	High [b]		Intermediate [b]		
	B (SE) [a]	P [a. c]	B (SE) [a]	P [a, c]	P-trend [a, c]
RT [d]	0.0081 (0.0014)	<0.0001	0.0028 (0.001)	0.0197	<0.0001
SB [d]	0.0073 (0.0013)	<0.0001	0.0025(0.001)	0.0197	<0.0001
SI [d]	0.0102 (0.0016)	<0.0001	0.0043 (0.0012)	0.0022	<0.0001
SD	1.24 (0.118)	<0.0001	0.475 (0.0892)	<0.0001	<0.0001
TMN [d]	0.0173 (0.0027)	<0.0001	0.0077 (0.0021)	0.0009	<0.0001
TMA [d]	0.0276 (0.0029)	<0.0001	0.011 (0.0022)	<0.0001	<0.0001
WM [d]	0.0115 (0.0017)	<0.0001	0.0059 (0.0013)	<0.0001	<0.0001
MX	0.609 (0.0445)	<0.0001	0.240 (0.0337)	<0.0001	<0.0001
QZ	0.374 (0.0435)	<0.0001	0.204 (0.0328)	<0.0001	<0.0001
VY	0.537 (0.0644)	<0.0001	0.198 (0.0486)	0.0003	<0.0001
PR [d]	0.0015 (0.0033)	0.64	-0.0002 (0.0025)	0.94	0.71
G6	0.301 (0.0290)	<0.0001	0.116 (0.0219)	<0.0001	<0.0001
G4	0.372 (0.0280)	<0.0001	0.164 (0.0211)	<0.0001	<0.0001

[a] RT: Reaction Test; SB: Stroop Box; SI: Stroop Ink; SD: Symbols Digit; TMN: Trail Making Numeric; TMA: Trail Making Alpha Numeric; MX: Matrices; WM: Working memory; QZ: Quiz; VY: Vocabulary; PR: Pairing7; G6: a summary measure computed using RT, SB, SI, SD, TMN and TMA; G4: a summary measure computed using WM, MX, QZ and VY; B: Beta; SE: Standard Error; P: two-tailed p-value; P-trend: two-tailed p-value for trend.
[b] Reference group: Low Multiple Deprivation Group; Linear regression models were commonly adjusted for age, age-square (except VY and PR), gender and types of devices used to take the test. An age by gender interaction term/s was included in the model for RT, SB, SI, SD, QZ, VY and G4.
[c] P-values were presented after Bonferroni Holm adjustment for 13 tests.
[d] Log10 transformation was performed for these test scores.

Supplementary Table 11 | The association between cognitive phenotypes (including measures of general cognitive ability) and clinical information available in G&C cohort.

Phenotypes [a]	Color Blindness [b]		Learning Disability [b]		Mental Health Issue [b]		High Blood Pressure [b]		Heart Problem [b]		Arthritis [b]		Diabetes [b]	
	B (SE) [a]	P [a, c]	B (SE) [a]	P [a, c]	B (SE) [a]	P [a, c]	B (SE) [a]	P [a, c]	B(SE)	P [a, c]	B(SE) [a]	P [a, c]	B (SE) [a]	P [a, c]
RT [d]	-0.0009 (0.0027)	1	0.0154 (0.0025)	<0.0001	0.0142 (0.0048)	0.0206	0.0033 (0.0016)	**0.14**	0.0108 (0.0035)	0.0181	0.0076 (0.0020)	0.0008	0.0153 (0.0028)	<0.0001
SB [d]	0.0071 (0.0026)	0.07	0.0225 (0.0023)	<0.0001	0.0140 (0.0045)	0.0141	0.0073 (0.0015)	<0.0001	0.0110 (0.0033)	0.0090	0.0082 (0.0019)	0.0001	0.0150 (0.0026)	<0.0001
SI [d]	0.0066 (0.0033)	0.46	0.0309 (0.0031)	<0.0001	0.0144 (0.0059)	0.06992	0.0093 (0.0020)	<0.0001	0.0124 (0.0043)	0.0243	0.0106 (0.0025)	0.0001	0.0190 (0.0034)	<0.0001
SD	0.4978 (0.2406)	0.42	2.6760 (0.2225)	<0.0001	1.4246 (0.4236)	0.0078	1.1309 (0.1440)	<0.0001	1.2741 (0.3112)	0.0005	1.1219 (0.1776)	<0.0001	1.7709 (0.2496)	<0.0001
TMN [d]	0.0040 (0.0055)	1	0.0495 (0.0051)	<0.0001	0.0135 (0.0098)	**0.26**	0.0071 (0.0033)	**0.13**	0.0197 (0.0072)	0.0243	0.0136 (0.0041)	0.0036	0.0181 (0.0057)	0.0065
TMA [d]	0.0028 (0.0059)	1	0.0810 (0.0055)	<0.0001	0.0381 (0.0104)	0.0032	0.0200 (0.0036)	<0.0001	0.0237 (0.0077)	0.0181	0.0289 (0.0044)	<0.0001	0.0451 (0.0061)	<0.0001
WM [d]	0.0029 (0.0036)	1	0.0504 (0.0033)	<0.0001	0.0212 (0.0063)	0.0078	0.0059 (0.0021)	0.0269	0.0089 (0.0046)	**0.16**	0.0105 (0.0026)	0.0004	0.0150 (0.0037)	0.0002
MX	0.2249 (0.0909)	0.16	0.5659 (0.0839)	<0.0001	0.2916 (0.1602)	**0.21**	0.3132 (0.0545)	<0.0001	0.3382 (0.1177)	0.0243	0.3143 (0.0672)	<0.0001	0.6082 (0.0939)	<0.0001
QZ	-0.0136 (0.0882)	1	1.0794 (0.0810)	<0.0001	0.4374 (0.1552)	0.0291	0.1514 (0.0529)	0.0250	0.3393 (0.1144)	0.0211	0.2114 (0.0651)	0.0036	0.2205 (0.0912)	0.0468
VY	0.0224 (0.1305)	1	1.1191 (0.1204)	<0.0001	0.3482 (0.2299)	**0.26**	0.1287 (0.0782)	**0.20**	0.3258 (0.1689)	**0.16**	0.0634 (0.0965)	**1**	0.2885 (0.1352)	**0.0657**
PR [d]	0.0063 (0.0067)	1	0.018 (0.0061)	0.0033	0.0277 (0.0117)	0.0721	0.0012 (0.0040)	**0.77**	-0.0006 (0.0086)	**0.94**	0.0023 (0.0049)	**1**	0.0031 (0.0069)	**0.65**
G6	0.1124 (0.0589)	0.50	0.7937 (0.0544)	<0.0001	0.4067 (0.1037)	0.0011	0.2279 (0.0354)	<0.0001	0.3473 (0.0763)	<0.0001	0.2938 (0.0436)	<0.0001	0.4850 (0.0612)	<0.0001
G4	0.0637 (0.0569)	1	0.8346 (0.0525)	<0.0001	0.3271 (0.1002)	0.0098	0.1587 (0.0342)	<0.0001	0.2510 (0.0737)	0.0073	0.1892 (0.0421)	<0.0001	0.3111 (0.0591)	<0.0001

[a] RT: Reaction Test; SB: Stroop Box; SI: Stroop Ink; SD: Symbols Digit; TMN: Trail Making Numeric; TMA: Trail Making Alpha Numeric; MX: Matrices; WM: Working memory; QZ: Quiz; VY: Vocabulary; PR: Pairing7; G6: a summary measure computed using RT, SB, SI, SD, TMN and TMA; G4: a summary measure computed using WM, MX, QZ and VY; B: Beta value from regression model; SE: Standard Error; P: two-tailed p-value.

[b] Each of the linear regression model was adjusted for age (centred to mean), age square, gender and types of devices used.

[c] P-value presented after Bonferroni–Holm corrections for 13 tests. Non-significant p-values are highlighted in black.

[d] Log10 transformation was performed for these test scores.

Supplementary Table 12 | The Association between cognitive traits (including a measure of general cognitive ability), age and APOE.

Phenotypes [a]					APOE				Age by APOE interactions							
	Age		Age^2		e2/e3 [b]		e4 carriers [b]		Age x e2/e3		Age x e4 carriers		Age^2 x e2/e3		Age^2 x e4 carriers	
	B (SE) [a]	P [a,c]	B (SE) [a]	P [a,c]	B (SE) [a]	P [a,c]	B (SE) [a]	P [a,c]	B (SE) [a]	P [a,c]	B (SE) [a]	P [a, c]	B (SE) [a]	P [a, c]	B (SE) [a]	P [a, c]
RT [d]	0.003	<0.0001	0.00002	<0.0001	-0.0009	1	0.002	1	0.0002	1	0.0002	0.39	0.00001	1	0.000008	1
	(0.00006)		(0.000004)		(0.002)		(0.002)		(0.0001)		(0.0001)		(0.000009)		(0.000007)	
SB [d]	0.003	<0.0001	0.00003	<0.0001	-0.0005	1	-0.0004	1	-0.000001	1	0.0002	0.39	-0.0000003	1	0.00001	0.55
	(0.00006)		(0.000004)		(0.003)		(0.002)		(0.0001)		(0.0001)		(0.000008)		(0.000006)	
SI [d]	0.003	<0.0001	0.00003	<0.0001	-0.008	**0.3#**	0.001	1	0.0002	1	0.0002	0.39	0.00003	**0.13#**	0.000004	1
	(0.00008)		(0.000005)		(0.003)		(0.003)		(0.0002)		(0.0001)		(0.00001)		(0.000008)	
SD	0.30	<0.0001	0.002	<0.0001	-0.02	1	-0.17	1	-0.01	1	0.02	**0.14#**	-0.0003	1	0.001	0.55
	(0.005)		(0.0003)		(0.25)		(0.19)		(0.01)		(0.009)		(0.0008)		(0.0006)	
TMN [d]	0.006	<0.0001	0.00005	<0.0001	-0.001	1	-0.0008	1	0.0002	1	-0.00003	0.87	0.00002	1	0.00001	1
	(0.0001)		(0.000008)		(0.006)		(0.004)		(0.0003)		(0.0002)		(0.00002)		(0.00001)	
TMA [d]	0.006	<0.0001	0.00008	<0.0001	0.002	1	-0.006	1	0.0001	1	0.0004	0.39	-0.000003	1	0.00003	0.55
	(0.0001)		(0.000008)		(0.006)		(0.005)		(0.0003)		(0.0002)		(0.00002)		(0.00001)	
QZ	0.02	<0.0001	0.0005	0.0002	0.03	1	-0.03	1	0.002	1	0.007	0.39	-0.0003	1	0.0002	1
	(0.002)		(0.0001)		(0.09)		(0.07)		(0.004)		(0.003)		(0.0003)		(0.0002)	
PR [d]	0.003	<0.0001	-	-	0.003	1	-0.001	0.6	-0.00006	1	0.0003	0.40	-	-	-	-
	(0.0001)				(0.005)		(0.004)	1	(0.0003)		(0.0003)					
G6	0.09	<0.0001	0.0008	<0.0001	-0.04	1	-0.02	1	0.002	1	0.005	**0.17#**	0.0002	1	0.0003	0.55
	(0.001)		(0.00008)		(0.06)		(0.05)		(0.003)		(0.002)		(0.0002)		(0.0001)	

[a] RT: Reaction Test; SB: Stroop Box; SI: Stroop Ink; SD: Symbols Digit; TMN: Trail Making Numeric; TMA: Trail Making Alpha Numeric; QZ: Quiz; PR: Pairing 7; G6: a summary measure computed using RT, SB, SI, SD, TMN and TMA; B: Beta value from linear mixed-effect model; SE: Standard Error; P: two-tailed p-value; Associations were adjusted for sex, the first five genomic principal components, devices, batch, array (as random effect).

[b] e3/e3 carriers were served as reference.

[c] P-values were presented after considering Bonferroni-Holm adjustment, considering the number of cognitive traits each covariate was tested against. P-values significant before correction are indicated with "#".

[d] Log10 transformation was performed for these test scores.

[#]Significant at <0.05 before Bonferroni Holm correction.

Supplementary Table 13 | Details of source polygenic risk score (PRS) and derived PRS in the G&C study.

No.	PGS ID	# SNPs in PRS	# SNPs used in G&C	PRS Ancestry	Source GWAS	APOE inclusion	Risk Groups in the present study
1	PGS002289	23	20	European	Lambert et al. (PMID: 24162737)	Yes (2 SNPs)	High (top 5th percentiles) Vs Low (≤ 95th percentile)
2	PGS002289	23	18	European		None	

Supplementary Table 14 | The Association between cognitive phenotypes (including a measure of general cognitive ability), age and Alzheimer's disease polygenic risk groups (included APOE).

Phenotypes [a]	Age		Age^2		PRS (Low) [b]		Age x PRS (Low)		Age^2 x PRS (Low)	
	B (SE) [a]	P [a, c]	B (SE) [a]	P [a, c]	B (SE) [a]	P [a, c]	B (SE) [a]	P [a, c]	B (SE) [a]	P [a, c]
RT [d]	0.003 (0.0002)	<0.0001	0.00003 (0.00001)	0.09	-0.0027 (0.0042)	1	-0.0001 (0.0002)	1	-0.000007 (0.00001)	1
SB [d]	0.004 (0.0002)	<0.0001	0.00006 (0.00001)	0.0001	0.001 (0.003)	1	-0.0004 (0.0002)	0.34[#]	-0.00003 (0.00001)	0.32
SI [d]	0.004 (0.0002)	<0.0001	0.00007 (0.00007)	0.0002	0.003 (0.005)	1	-0.0007 (0.0003)	**0.039**[##]	-0.00004 (0.00002)	0.28[#]
SD	0.33 (0.02)	<0.0001	0.005 (0.001)	0.0002	0.52 (0.36)	1	-0.03 (0.02)	0.56	-0.002 (0.001)	0.30
TMN [d]	0.006 (0.0004)	<0.0001	0.0001 (0.00003)	0.0017	0.004 (0.009)	1	-0.0001 (0.0004)	1	-0.00005 (0.00003)	0.42
TMA [d]	0.006 (0.0005)	<0.0001	0.0001 (0.00003)	<0.0001	0.01 (0.009)	1	-0.0005 (0.0005)	0.89	-0.00004 (0.00003)	0.53
QZ	0.03 (0.007)	<0.0001	0.0007 (0.0005)	0.10	0.02 (0.14)	1	-0.01 (0.007)	0.56	-0.0002 (0.0005)	1
PR [d]	0.004 (0.0005)	<0.0001	-	-	0.0006 (0.007)	1	-0.0008 (0.0005)	0.56	-	-
G6	0.1 (0.005)	<0.0001	0.002 (0.0003)	<0.0001	0.09 (0.09)	1	-0.009 (0.005)	0.34[#]	-0.0007 (0.0003)	0.19[#]

[a] RT: Reaction Test; SB: Stroop Box; SI: Stroop Ink; SD: Symbols Digit; TMN: Trail Making Numeric; TMA: Trail Making Alpha Numeric; QZ: Quiz; PR: Pairing7; G6: a summary measure computed using RT, SB, SI, SD, TMN and TMA; B: Beta value from linear mixed effect model; SE: Standard Error; P: two-tailed p-value; Associations were adjusted for sex, the first five genomic principal components, devices, batch, array (as random effect).

[b] High PRS group was used as reference.

[c] P-values are presented after Bonferroni–Holm corrections for 9 tests. P-values significant before the multiple testing correction are indicated with "#".

[d] Log10 transformation was performed for these test scores.

[#] Significant at <0.05 before Bonferroni Holm correction.

[##] Significant at <0.01 before Bonferroni Holm correction.

Supplementary Table 15 | The Association between cognitive tests (Vocabulary, Reaction test, Stroop Box and Trail-making Numeric), age and Alzheimer's disease polygenic risk groups (APOE region excluded).

Phenotypes [a]	Age		Age^2		PRS (Low) [b]		Age x PRS (Low)		Age^2 x PRS (Low)	
	B (SE) [a]	P [a, c]	B (SE) [a]	P [a, c]	B (SE) [a]	P [a, c]	B (SE) [a]	P [a, c]	B (SE) [a]	P [a, c]
RT [d]	0.0028 (0.0002)	<0.0001	0.00002 (0.00001)	0.08	-0.001 (0.004)	1	0.0001 (0.0002)	1	-0.000001 (0.00001)	0.92
SB [d]	0.003 (0.0002)	<0.0001	0.00004 (0.00001)	0.0013	0.003 (0.004)	1	0.00005 (0.0002)	1	-0.00001 (0.00001)	0.86
TMN [d]	0.006 (0.0004)	<0.0001	0.0001 (0.00003)	0.0008	0.003 (0.008)	1	-0.0005 (0.0004)	1	-0.00004 (0.00003)	0.28
VY	-0.05 (0.009)	<0.0001	-	-	-0.28 (0.14)	0.20	-0.004 (0.01)	1	-	-

[a] RT: Reaction Test; SB: Stroop Box; TMN: Trail Making Numeric; VY: Vocabulary; B: Beta value from linear mixed effect model; SE: Standard Error; P: two-tailed p-value; Associations were adjusted for sex, the first five genomic principal components, devices, batch, array (as random effect).
[b] High PRS group was used as reference.
[c] P-values are presented after Bonferroni–Holm corrections for 4 tests.
[d] Log10 transformation was performed for these test scores.

Supplementary Table 16 | Heritability for cognitive tests and two summary measures of general cognitive ability (G4 and G6) in G&C cohort.

Phenotypes	N	Transformation	Heritability[1]
Reaction Test	10,005	log10(RT)	0.11 (0.04)
Stroop Box	10,000	log10(RT)	0.15 (0.04)
Stroop Ink	9,983	log10(RT)	0.15 (0.04)
Symbol Digits	9,921	No	0.07 (0.04)
Trail Making: Numeric	9,930	log10(RT)	0.14 (0.04)
Trail Making: Alpha Numeric	9,930	log10(RT)	0.21 (0.04)
Matrix	9,981	No	0.17 (0.04)
Working Memory	10,019	log10(RT)	0.06 (0.04)
Quiz	10,022	No	0.23 (0.04)
Vocabulary	9,971	No	0.23 (0.04)
Paring7	10,013	log10(RT)	0.14 (0.04)
G6	9,879	No	0.19 (0.04)
G4	9,879	No	0.28 (0.04)

[1] Heritability estimates are derived using individual level data.

Supplementary Table 17 | Summary of covariates adjusted in the heritability and genome-wide association analysis.

Phenotypes	Covariates Adjusted
Reaction Test	Age, Age^2, Sex, Age*Sex, Age^2*Sex, Device used for cognitive test, Batch, Array, GPCs (1-20)
Stroop Box	Age, Age^2, Sex, Age*Sex, Device used for cognitive test, Batch, Array, GPCs (1-20)
Stroop Ink	Age, Age^2, Sex, Age^2*Sex, Device used for cognitive test, Batch, Array, GPCs (1-20)
Symbol Digits	Age, Age^2, Sex, Age*Sex, Age^2*Sex, Device used for cognitive test, Batch, Array, GPCs (1-20)
Trail Making: Numeric	Age, Age^2, Sex, Device used for cognitive test, Batch, Array, GPCs (1-20)
Trail Making: Alpha Numeric	Age, Age^2, Sex, Device used for cognitive test, Batch, Array, GPCs (1-20)
Matrix	Age, Age^2, Sex, Device used for cognitive test, Batch, Array, GPCs (1-20)
Working Memory	Age, Age^2, Sex, Batch, Array, GPCs (1-20)
Quiz	Age, Age^2, Sex, Age*Sex, Device used for cognitive test, Batch, Array, GPCs (1-20)
Vocabulary	Age, Sex, Age*Sex, Device used for cognitive test, Batch, Array, GPCs (1-20)
Paring7	Age, Sex, Device used for cognitive test, Batch, Array, GPCs (1-20)
G6	Age, Age^2, Sex, Device used for cognitive test, Batch, Array, GPCs (1-20)
G4	Age, Age^2, Sex, Age*Sex, Device used for cognitive test, Batch, Array, GPCs (1-20)

Supplementary Table 18 | Independent SNPs significantly associated with G4 and G6 in Genes and Cognition cohort.

Phenotypes	SNP	CHR: BP	A1	A2	FREQ	BETA	SE	P	INFO
G4	rs62034351	16:28565489	G	A	0.648	-0.1141	0.0199	9.1E-09	0.98
G6	rs11705789	3:81969196	G	T	0.641	-0.1113	0.0203	4.5E-08	0.99

The independent SNPs at GWAS significant locus were reported with RSID; genomic coordinates (CHR: BP; GRCh37.p13/ Hg19/); A1, effect allele; A2, other allele; FREQ, effect allele frequency; Beta, coefficient from BOLT infinitesimal model; SE, standard error; P, two-tailed p-value; INFO, estimated imputation score.

Supplementary Table 19 | G4 associated genome-wide significant SNP list.

SNP	CHR	BP	A1	A2	FREQ	INFO	P	BETA	SE
rs149299	16	28485141	T	C	0.610634	0.982413	9.50E-09	-0.11175	0.019468
rs151179	16	28487056	A	G	0.609943	0.982619	1.40E-08	-0.11047	0.019464
rs151180	16	28487213	G	T	0.587392	0.952882	2.80E-08	-0.10897	0.019611
rs181195	16	28488369	A	T	0.610613	0.982556	9.70E-09	-0.11165	0.019466
rs151181	16	28490517	T	C	0.605517	0.998356	1.30E-08	-0.10947	0.019257
rs151303	16	28492510	C	A	0.610498	0.984459	9.20E-09	-0.1117	0.019444
rs34838	16	28494350	A	G	0.572067	0.981827	4.80E-08	-0.10483	0.019202
rs42861	16	28494421	A	G	0.57205	0.981582	4.70E-08	-0.10492	0.019203
rs34836	16	28494823	A	G	0.571975	0.98181	4.60E-08	-0.10498	0.019203
rs151182	16	28495752	C	T	0.61014	0.983282	1.10E-08	-0.11104	0.019453
rs151301	16	28497059	T	A	0.61031	0.98362	9.40E-09	-0.11169	0.019452
rs34835	16	28499291	A	G	0.602986	0.982966	4.20E-08	-0.1063	0.019395
rs27741	16	28504181	G	A	0.6039	0.982207	3.20E-08	-0.10732	0.019404
rs149271	16	28506872	A	G	0.6102	0.982787	2.20E-08	-0.10911	0.019494
rs180743	16	28507644	C	G	0.610086	0.982701	1.50E-08	-0.11034	0.019495
rs180744	16	28508048	A	G	0.609811	0.982214	1.70E-08	-0.10991	0.0195
rs151174	16	28508069	C	T	0.610733	0.982393	1.20E-08	-0.11113	0.019497
rs240704	16	28529949	T	C	0.600502	0.983982	4.40E-08	-0.10586	0.019344
rs4787458	16	28531287	A	G	0.634545	0.984722	4.70E-08	-0.10763	0.019709
rs3785354	16	28550667	C	T	0.647662	0.983046	1.20E-08	-0.11321	0.01986
rs4787457	16	28555400	A	G	0.647685	0.983098	1.10E-08	-0.11335	0.019861
rs9926245	16	28555706	T	A	0.604767	0.985439	3.90E-08	-0.10669	0.019407
rs4788081	16	28556147	T	C	0.647769	0.983058	1.00E-08	-0.11383	0.01986
rs4788080	16	28558081	C	T	0.647682	0.98313	1.20E-08	-0.11333	0.01986
rs4788079	16	28559363	C	A	0.647763	0.983023	1.20E-08	-0.11334	0.019861
rs4787456	16	28559573	C	T	0.647792	0.982953	1.10E-08	-0.11347	0.019863
rs4788078	16	28559981	G	A	0.647773	0.983005	1.10E-08	-0.11344	0.019863
rs13331170	16	28561982	T	C	0.604795	0.985073	3.30E-08	-0.10727	0.019414
rs62034351	16	28565489	G	A	0.648	0.982706	9.10E-09	-0.11413	0.019862
rs7191618	16	28565667	C	G	0.604994	0.984285	3.40E-08	-0.10725	0.019421
rs7187604	16	28565953	A	G	0.647188	0.982929	1.50E-08	-0.11231	0.019845
rs3859172	16	28566158	G	T	0.604978	0.984441	3.40E-08	-0.10723	0.019421
rs4788076	16	28570005	C	T	0.647809	0.981725	1.70E-08	-0.11205	0.01986
rs56272201	16	28580685	A	G	0.648	0.98157	1.60E-08	-0.11222	0.019864
rs56396270	16	28580829	C	T	0.647897	0.981691	2.00E-08	-0.1115	0.019863

rs12447461	16	28582941	C	A	0.637043	0.982737	4.40E-08	-0.10828	0.019784
rs112191041	16	28583362	A	G	0.637077	0.982628	4.60E-08	-0.10818	0.019785
rs111693583	16	28583610	C	T	0.637079	0.982618	4.60E-08	-0.10818	0.019785
rs11074911	16	28583865	C	T	0.637079	0.982618	4.60E-08	-0.10818	0.019785
rs56209193	16	28585636	C	T	0.63708	0.982625	4.30E-08	-0.10839	0.019785
rs7193402	16	28586127	T	C	0.643778	0.998521	1.90E-08	-0.11034	0.019641
rs62034358	16	28587597	G	T	0.6479	0.981682	2.00E-08	-0.11152	0.019863
rs12445823	16	28588049	A	C	0.647897	0.981691	2.00E-08	-0.1115	0.019863
rs12445744	16	28588395	A	C	0.647896	0.981692	2.00E-08	-0.1115	0.019863
rs12448429	16	28588443	C	T	0.63704	0.982744	4.50E-08	-0.10825	0.019784
rs4787455	16	28589455	G	A	0.648117	0.980934	1.90E-08	-0.11165	0.019876
rs112047757	16	28590862	C	T	0.637151	0.982128	4.10E-08	-0.1086	0.019789
rs112831778	16	28590989	C	T	0.648175	0.980824	1.40E-08	-0.11265	0.019873
rs17707300	16	28593347	T	C	0.632803	0.999981	3.90E-08	-0.10751	0.019562
rs4788074	16	28593597	G	A	0.648062	0.980927	1.80E-08	-0.11188	0.019873
rs4788073	16	28594549	A	G	0.637096	0.982126	4.00E-08	-0.10868	0.01979
rs17640009	16	28595700	A	G	0.637107	0.982111	4.00E-08	-0.1087	0.01979
rs75539558	16	28595810	C	G	0.637112	0.98209	3.90E-08	-0.10872	0.019791
rs62034359	16	28600091	C	T	0.648447	0.979017	2.10E-08	-0.11158	0.019905
rs2077031	16	28603168	T	A	0.648442	0.978499	1.80E-08	-0.11214	0.01991
rs762634	16	28603335	T	C	0.648615	0.978091	1.80E-08	-0.11209	0.019916
rs710410	16	28603342	A	G	0.648414	0.977706	2.20E-08	-0.11146	0.019922
rs1059491	16	28603655	T	G	0.64839	0.978617	2.00E-08	-0.11179	0.01991
rs113208333	16	28606160	G	T	0.637488	0.97983	4.80E-08	-0.10822	0.019822
rs4115668	16	28607532	G	A	0.671833	0.953518	4.10E-08	-0.11253	0.020507
rs762633	16	28608341	T	C	0.649409	0.973221	3.80E-08	-0.11001	0.019994
rs35175818	16	28608746	A	C	0.649328	0.972972	3.80E-08	-0.11003	0.019998
rs4149398	16	28608938	C	G	0.649363	0.972747	3.60E-08	-0.1102	0.020002
rs2925635	16	28622993	A	G	0.396312	0.883702	3.90E-08	0.113497	0.020652
rs28729187	16	28626966	T	C	0.372492	0.948165	1.20E-08	0.11349	0.019928
rs28676837	16	28627063	C	T	0.372421	0.948147	1.30E-08	0.113317	0.019927
rs6498089	16	28629300	C	T	0.372496	0.948211	1.20E-08	0.113483	0.019928
rs28410083	16	28631360	A	T	0.367907	0.947687	3.80E-08	0.109762	0.01995
rs1968751	16	28631759	T	C	0.367722	0.947943	3.70E-08	0.10981	0.019952
rs8049739	16	28632288	G	C	0.367671	0.947411	3.50E-08	0.110068	0.019967
rs12446008	16	28644459	C	T	0.727563	0.933254	1.20E-08	-0.12521	0.021945
rs9929198	16	28644980	G	A	0.726369	0.932275	1.50E-08	-0.1243	0.021937
rs111820348	16	28645127	C	T	0.729977	0.930013	1.70E-08	-0.12441	0.022063
rs3987654	16	28647568	T	C	0.72705	0.921541	1.20E-08	-0.12584	0.022068

rs72793807	16	28651732	C	T	0.728273	0.921134	1.10E-08	-0.1262	0.022105

SNP: Single Nucleotide Polymorphism; CHR: Chromosome; A1, Effect allele; A2, Other allele; FREQ: Effect allele frequency; Beta: linear regression coefficient; SE: standard error; INFO: Estimated imputation score.

Supplementary Table 20 | G4 associated SNPs at the suggestive threshold of significance (1 x 10^{-06}).

SNP	CHR	BP	A1	A2	FREQ	INFO	P	BETA	SE
rs864686	5	32498562	A	T	0.622323	0.975189	9.40E-08	-0.10461	0.019597
rs9376020	6	1.35E+08	C	A	0.775149	0.861135	7.80E-07	-0.12089	0.024473
rs6913681	6	1.35E+08	C	A	0.774394	0.849165	3.70E-07	-0.12536	0.024665
rs6490577	13	21012107	T	C	0.328958	0.999218	6.60E-07	0.098703	0.019853
rs34720574	13	21012549	C	T	0.673167	0.991886	6.90E-07	-0.0991	0.019961
rs6490578	13	21012811	T	C	0.332455	0.983647	8.90E-07	0.098114	0.019961
rs4773767	13	94571840	C	T	0.693269	0.974839	4.30E-07	-0.10389	0.020543
rs928187	13	94573380	G	T	0.692973	0.975079	4.00E-07	-0.10411	0.020535
rs928186	13	94573671	C	T	0.692867	0.975161	4.70E-07	-0.10339	0.020523
rs17174742	13	94574965	T	C	0.692742	0.975153	4.70E-07	-0.10342	0.02052
rs6492682	13	94576572	G	C	0.311995	0.951049	9.30E-07	0.101517	0.020695
rs9561473	13	94582160	T	G	0.692573	0.975374	4.70E-07	-0.10336	0.020516
rs9561477	13	94584521	A	C	0.691027	0.987405	3.50E-07	-0.10364	0.020343
rs2389077	13	94585426	G	A	0.698482	0.985949	7.10E-07	-0.10148	0.020466
rs9556341	13	94587427	G	A	0.691363	0.987258	4.10E-07	-0.10312	0.020354
rs945936	13	94587909	G	A	0.654839	0.987428	7.90E-07	-0.09788	0.019817
rs1888229	13	94588358	A	G	0.691171	0.987682	3.60E-07	-0.10345	0.020336
rs878765	13	94589164	A	G	0.691152	0.987753	3.70E-07	-0.10341	0.020334
rs9524281	13	94591226	G	T	0.316584	0.964171	5.40E-07	0.102544	0.020463
rs7332610	13	94592768	T	G	0.653819	0.988008	9.00E-07	-0.09731	0.01981
rs73541965	13	94594671	T	C	0.653772	0.987988	8.90E-07	-0.09732	0.019807
rs11619935	13	94595305	T	C	0.651069	0.999293	7.90E-07	-0.09706	0.019661
rs1591697	13	94610986	C	T	0.311335	0.968706	6.30E-07	0.102384	0.020557
rs1337828	13	94612361	C	T	0.304236	0.999821	6.70E-07	0.101164	0.020351
rs9524296	13	94629619	T	C	0.310196	0.968209	5.30E-07	0.10323	0.020584
rs2726034	16	28336882	T	C	0.684117	0.968008	7.90E-07	-0.10184	0.020625
rs12598357	16	28340945	A	G	0.59892	0.95717	5.40E-07	-0.09802	0.01956
rs2726036	16	28347140	A	C	0.618688	0.957724	8.60E-07	-0.09724	0.019763
rs140512382	16	28350730	G	A	0.803732	0.716586	2.60E-07	-0.14816	0.028782
rs62032566	16	28383243	C	T	0.605425	0.948532	8.70E-07	-0.09748	0.019813
rs149299	16	28485141	T	C	0.610634	0.982413	9.50E-09	-0.11175	0.019468
rs151179	16	28487056	A	G	0.609943	0.982619	1.40E-08	-0.11047	0.019464

rs151180	16	28487213	G	T	0.587392	0.952882	2.80E-08	-0.10897	0.019611
rs181195	16	28488369	A	T	0.610613	0.982556	9.70E-09	-0.11165	0.019466
rs34839	16	28489346	T	C	0.57279	0.981387	5.00E-08	-0.10474	0.019213
rs151181	16	28490517	T	C	0.605517	0.998356	1.30E-08	-0.10947	0.019257
rs151303	16	28492510	C	A	0.610498	0.984459	9.20E-09	-0.1117	0.019444
rs34838	16	28494350	A	G	0.572067	0.981827	4.80E-08	-0.10483	0.019202
rs42861	16	28494421	A	G	0.57205	0.981582	4.70E-08	-0.10492	0.019203
rs34836	16	28494823	A	G	0.571975	0.98181	4.60E-08	-0.10498	0.019203
rs151182	16	28495752	C	T	0.61014	0.983282	1.10E-08	-0.11104	0.019453
rs151301	16	28497059	T	A	0.61031	0.98362	9.40E-09	-0.11169	0.019452
rs34835	16	28499291	A	G	0.602986	0.982966	4.20E-08	-0.1063	0.019395
rs27741	16	28504181	G	A	0.6039	0.982207	3.20E-08	-0.10732	0.019404
rs149271	16	28506872	A	G	0.6102	0.982787	2.20E-08	-0.10911	0.019494
rs180743	16	28507644	C	G	0.610086	0.982701	1.50E-08	-0.11034	0.019495
rs180744	16	28508048	A	G	0.609811	0.982214	1.70E-08	-0.10991	0.0195
rs151174	16	28508069	C	T	0.610733	0.982393	1.20E-08	-0.11113	0.019497
rs40831	16	28508447	A	G	0.592101	0.973879	7.00E-08	-0.10444	0.019371
rs40834	16	28510393	C	T	0.555908	0.982859	2.70E-07	-0.09834	0.019115
rs40836	16	28510537	A	G	0.555693	0.98374	2.40E-07	-0.0987	0.019105
rs40837	16	28510845	A	G	0.555832	0.983885	2.50E-07	-0.09848	0.0191
rs181203	16	28512371	A	C	0.679905	0.976844	5.00E-08	-0.11187	0.020523
rs181205	16	28513129	T	C	0.668011	0.957617	2.20E-07	-0.10653	0.020545
rs181206	16	28513403	A	G	0.681938	0.974675	6.60E-08	-0.11123	0.020586
rs181209	16	28514854	G	T	0.680243	0.978748	5.60E-08	-0.11143	0.020512
rs26528	16	28517709	T	C	0.552044	0.999696	2.80E-07	-0.09725	0.018934
rs153109	16	28519096	T	C	0.55578	0.985117	2.80E-07	-0.09802	0.019091
rs56354901	16	28523144	T	C	0.675396	0.984719	2.80E-07	-0.10439	0.020328
rs240707	16	28524612	C	G	0.555267	0.984678	3.70E-07	-0.09706	0.019093
rs153106	16	28526897	T	C	0.600995	0.985513	6.10E-08	-0.10481	0.019355
rs62034318	16	28527221	C	T	0.674715	0.984879	3.40E-07	-0.10365	0.020318
rs79046494	16	28527326	G	A	0.674715	0.984879	3.40E-07	-0.10365	0.020318
rs28449958	16	28528781	G	A	0.673306	0.981393	2.90E-07	-0.10431	0.020324
rs240705	16	28529716	C	T	0.600989	0.985497	6.20E-08	-0.10479	0.019355
rs240704	16	28529949	T	C	0.600502	0.983982	4.40E-08	-0.10586	0.019344
rs4787458	16	28531287	A	G	0.634545	0.984722	4.70E-08	-0.10763	0.019709
rs240702	16	28531353	C	T	0.601383	0.985159	7.50E-08	-0.10413	0.01936
rs62034319	16	28532188	T	G	0.601645	0.984553	6.10E-08	-0.10486	0.019364
rs28698667	16	28533071	C	T	0.602295	0.985357	7.20E-08	-0.10429	0.019367
rs62034321	16	28534038	C	T	0.602339	0.985625	8.00E-08	-0.10395	0.019369

rs4788085	16	28535003	A	G	0.602247	0.985596	7.40E-08	-0.10422	0.019371
rs28772958	16	28535305	A	G	0.60225	0.985582	7.40E-08	-0.10423	0.019371
rs62034322	16	28535834	G	A	0.634009	0.985135	6.20E-08	-0.10671	0.019719
rs62034323	16	28536473	C	T	0.602338	0.985641	8.10E-08	-0.10391	0.019368
rs2106480	16	28537971	T	C	0.602232	0.985565	7.50E-08	-0.1042	0.019371
rs62034325	16	28538640	A	G	0.63463	0.985241	5.70E-08	-0.10708	0.019723
rs4788084	16	28539848	C	T	0.598895	0.999943	8.10E-08	-0.10301	0.0192
rs12446550	16	28543381	G	A	0.60238	0.985869	8.30E-08	-0.1038	0.019366
rs4788083	16	28545449	A	G	0.602226	0.985806	8.20E-08	-0.10388	0.019369
rs3785354	16	28550667	C	T	0.647662	0.983046	1.20E-08	-0.11321	0.01986
rs4787457	16	28555400	A	G	0.647685	0.983098	1.10E-08	-0.11335	0.019861
rs9926245	16	28555706	T	A	0.604767	0.985439	3.90E-08	-0.10669	0.019407
rs4788081	16	28556147	T	C	0.647769	0.983058	1.00E-08	-0.11383	0.01986
rs4788080	16	28558081	C	T	0.647682	0.98313	1.20E-08	-0.11333	0.01986
rs4788079	16	28559363	C	A	0.647763	0.983023	1.20E-08	-0.11334	0.019861
rs4787456	16	28559573	C	T	0.647792	0.982953	1.10E-08	-0.11347	0.019863
rs4788078	16	28559981	G	A	0.647773	0.983005	1.10E-08	-0.11344	0.019863
rs13331170	16	28561982	T	C	0.604795	0.985073	3.30E-08	-0.10727	0.019414
rs62034351	16	28565489	G	A	0.648	0.982706	9.10E-09	-0.11413	0.019862
rs7191618	16	28565667	C	G	0.604994	0.984285	3.40E-08	-0.10725	0.019421
rs7187604	16	28565953	A	G	0.647188	0.982929	1.50E-08	-0.11231	0.019845
rs3859172	16	28566158	G	T	0.604978	0.984441	3.40E-08	-0.10723	0.019421
rs4788076	16	28570005	C	T	0.647809	0.981725	1.70E-08	-0.11205	0.01986
rs56272201	16	28580685	A	G	0.648	0.98157	1.60E-08	-0.11222	0.019864
rs56396270	16	28580829	C	T	0.647897	0.981691	2.00E-08	-0.1115	0.019863
rs12447461	16	28582941	C	A	0.637043	0.982737	4.40E-08	-0.10828	0.019784
rs112191041	16	28583362	A	G	0.637077	0.982628	4.60E-08	-0.10818	0.019785
rs111693583	16	28583610	C	T	0.637079	0.982618	4.60E-08	-0.10818	0.019785
rs11074911	16	28583865	C	T	0.637079	0.982618	4.60E-08	-0.10818	0.019785
rs56209193	16	28585636	C	T	0.63708	0.982625	4.30E-08	-0.10839	0.019785
rs7193402	16	28586127	T	C	0.643778	0.998521	1.90E-08	-0.11034	0.019641
rs62034358	16	28587597	G	T	0.6479	0.981682	2.00E-08	-0.11152	0.019863
rs12445823	16	28588049	A	C	0.647897	0.981691	2.00E-08	-0.1115	0.019863
rs12445744	16	28588395	A	C	0.647896	0.981692	2.00E-08	-0.1115	0.019863
rs12448429	16	28588443	C	T	0.63704	0.982744	4.50E-08	-0.10825	0.019784
rs4787455	16	28589455	G	A	0.648117	0.980934	1.90E-08	-0.11165	0.019876
rs112047757	16	28590862	C	T	0.637151	0.982128	4.10E-08	-0.1086	0.019789
rs112831778	16	28590989	C	T	0.648175	0.980824	1.40E-08	-0.11265	0.019873
rs17707300	16	28593347	T	C	0.632803	0.999981	3.90E-08	-0.10751	0.019562

rs4788074	16	28593597	G	A	0.648062	0.980927	1.80E-08	-0.11188	0.019873
rs4788073	16	28594549	A	G	0.637096	0.982126	4.00E-08	-0.10868	0.01979
rs17640009	16	28595700	A	G	0.637107	0.982111	4.00E-08	-0.1087	0.01979
rs75539558	16	28595810	C	G	0.637112	0.98209	3.90E-08	-0.10872	0.019791
rs55792032	16	28599411	A	G	0.637323	0.980279	5.00E-08	-0.10805	0.019816
rs62034359	16	28600091	C	T	0.648447	0.979017	2.10E-08	-0.11158	0.019905
rs2077031	16	28603168	T	A	0.648442	0.978499	1.80E-08	-0.11214	0.01991
rs762634	16	28603335	T	C	0.648615	0.978091	1.80E-08	-0.11209	0.019916
rs710410	16	28603342	A	G	0.648414	0.977706	2.20E-08	-0.11146	0.019922
rs1059491	16	28603655	T	G	0.64839	0.978617	2.00E-08	-0.11179	0.01991
rs113208333	16	28606160	G	T	0.637488	0.97983	4.80E-08	-0.10822	0.019822
rs4149406	16	28607070	A	G	0.638314	0.973757	6.90E-08	-0.10735	0.019904
rs4115668	16	28607532	G	A	0.671833	0.953518	4.10E-08	-0.11253	0.020507
rs743590	16	28608230	G	A	0.638415	0.97399	7.60E-08	-0.10701	0.019903
rs762633	16	28608341	T	C	0.649409	0.973221	3.80E-08	-0.11001	0.019994
rs35175818	16	28608746	A	C	0.649328	0.972972	3.80E-08	-0.11003	0.019998
rs4149398	16	28608938	C	G	0.649363	0.972747	3.60E-08	-0.1102	0.020002
rs62031562	16	28609329	A	T	0.644192	0.963439	1.30E-07	-0.106	0.020063
rs11074907	16	28613965	A	G	0.629262	0.941346	5.30E-07	-0.10111	0.020168
rs7191548	16	28614734	T	C	0.628302	0.942454	4.20E-07	-0.10201	0.020157
rs6498091	16	28616259	T	G	0.627929	0.942687	3.70E-07	-0.10247	0.020152
rs4788070	16	28616399	G	A	0.628131	0.942629	4.90E-07	-0.10141	0.020156
rs4788069	16	28616665	C	A	0.628958	0.941627	4.80E-07	-0.10149	0.020168
rs4788068	16	28616723	C	T	0.62901	0.94143	5.10E-07	-0.10136	0.020176
rs1042157	16	28617057	G	A	0.629009	0.941491	5.10E-07	-0.1013	0.020174
rs1042028	16	28617514	C	T	0.677634	0.933892	9.10E-07	-0.10237	0.020852
rs4149394	16	28617574	C	T	0.631721	0.937004	3.20E-07	-0.10343	0.020235
rs79039694	16	28617795	G	A	0.631223	0.937028	5.20E-07	-0.10168	0.020252
rs4149392	16	28617813	A	G	0.631563	0.936497	5.70E-07	-0.1013	0.020259
rs75301646	16	28617885	G	A	0.647444	0.914893	9.60E-07	-0.10138	0.020692
rs116840534	16	28617890	A	G	0.647752	0.915012	7.00E-07	-0.10259	0.020683
rs41278158	16	28617993	C	A	0.771106	0.759051	6.00E-08	-0.14271	0.026341
rs74752114	16	28618068	G	A	0.741344	0.793659	2.90E-07	-0.1258	0.024509
rs9282862	16	28618237	T	C	0.629178	0.94049	4.90E-07	-0.10148	0.02018
rs3020804	16	28618636	A	G	0.628802	0.940673	4.90E-07	-0.10154	0.02018
rs3020803	16	28618914	T	A	0.629437	0.940927	3.70E-07	-0.10259	0.020179
rs2925629	16	28619076	T	C	0.629315	0.940823	3.30E-07	-0.10304	0.02018
rs2925630	16	28619132	T	C	0.629388	0.940833	3.10E-07	-0.10319	0.020177
rs1126447	16	28619911	T	C	0.628982	0.940799	4.20E-07	-0.10208	0.020182

rs9922110	16	28622154	C	G	0.600738	0.859984	5.70E-07	0.105222	0.021035
rs2925635	16	28622993	A	G	0.396312	0.883702	3.90E-08	0.113497	0.020652
rs28729187	16	28626966	T	C	0.372492	0.948165	1.20E-08	0.11349	0.019928
rs28676837	16	28627063	C	T	0.372421	0.948147	1.30E-08	0.113317	0.019927
rs6498089	16	28629300	C	T	0.372496	0.948211	1.20E-08	0.113483	0.019928
rs28410083	16	28631360	A	T	0.367907	0.947687	3.80E-08	0.109762	0.01995
rs62031607	16	28631530	A	T	0.649717	0.975208	5.20E-08	-0.10826	0.019889
rs1968752	16	28631585	T	G	0.355565	0.996915	6.80E-08	0.105811	0.019606
rs1968751	16	28631759	T	C	0.367722	0.947943	3.70E-08	0.10981	0.019952
rs2411453	16	28632021	T	G	0.397937	0.947108	1.20E-07	0.104539	0.019751
rs8049739	16	28632288	G	C	0.367671	0.947411	3.50E-08	0.110068	0.019967
rs12446008	16	28644459	C	T	0.727563	0.933254	1.20E-08	-0.12521	0.021945
rs9929198	16	28644980	G	A	0.726369	0.932275	1.50E-08	-0.1243	0.021937
rs111820348	16	28645127	C	T	0.729977	0.930013	1.70E-08	-0.12441	0.022063
rs3987654	16	28647568	T	C	0.72705	0.921541	1.20E-08	-0.12584	0.022068
rs35626515	16	28649651	C	A	0.614044	0.969915	5.90E-08	-0.10643	0.019633
rs72793807	16	28651732	C	T	0.728273	0.921134	1.10E-08	-0.1262	0.022105
rs188237080	16	28718755	A	G	0.673244	0.878849	1.50E-07	-0.11295	0.021486
rs378421	16	28754684	G	A	0.603625	0.966571	1.50E-07	-0.10276	0.019575
rs62036613	16	28823097	A	G	0.618028	0.983224	5.80E-07	-0.09772	0.019552
rs62036614	16	28824685	T	C	0.614185	0.99997	5.40E-07	-0.09695	0.019346
rs113029997	16	28825100	T	C	0.617078	0.984578	8.40E-07	-0.09621	0.019532
rs11860513	16	28825420	C	T	0.617078	0.984578	8.40E-07	-0.09621	0.019532
rs2008514	16	28825605	G	A	0.617078	0.984578	8.40E-07	-0.09621	0.019532
rs1987472	16	28825777	C	T	0.617078	0.984578	8.40E-07	-0.09621	0.019532
rs56186137	16	28825953	A	G	0.617108	0.984517	8.40E-07	-0.09621	0.019534
rs62036616	16	28826307	A	G	0.617941	0.984587	5.30E-07	-0.09796	0.019534
rs62036617	16	28827498	T	C	0.61713	0.984579	7.70E-07	-0.09652	0.019531
rs62036618	16	28828834	A	C	0.616861	0.985663	6.80E-07	-0.09697	0.019521
rs4788095	16	28831359	T	C	0.616865	0.985677	6.80E-07	-0.09696	0.019521
rs72793809	16	28832382	C	T	0.613378	0.999975	6.90E-07	-0.09605	0.019347
rs62036620	16	28833097	A	G	0.616859	0.9857	6.80E-07	-0.09696	0.019521
rs62036621	16	28833299	C	A	0.616859	0.9857	6.80E-07	-0.09696	0.019521
rs4451951	16	28836205	T	C	0.616746	0.985681	6.70E-07	-0.09702	0.019523
rs62036622	16	28837203	T	G	0.617428	0.985577	4.90E-07	-0.09821	0.019525
rs8049439	16	28837515	T	C	0.612652	0.99995	4.40E-07	-0.09772	0.019348
rs8062405	16	28837906	A	G	0.616362	0.99992	5.80E-07	-0.09681	0.019372
rs72793811	16	28839392	G	A	0.620567	0.985917	4.90E-07	-0.09835	0.019552
rs78613234	16	28839425	T	C	0.619763	0.985928	7.10E-07	-0.09691	0.019548

rs62036623	16	28839735	C	G	0.620563	0.985919	4.90E-07	-0.09834	0.019552
rs62036624	16	28839930	G	T	0.619719	0.985894	6.80E-07	-0.09709	0.019545
rs72793812	16	28840381	A	G	0.617556	0.985534	4.70E-07	-0.09835	0.019528
rs12443881	16	28841777	C	T	0.617215	0.999986	4.80E-07	-0.09758	0.019379
rs55991577	16	28843025	C	T	0.619876	0.985917	7.20E-07	-0.09691	0.01955
rs56358680	16	28843118	A	G	0.619872	0.985911	7.20E-07	-0.09691	0.01955
rs62036626	16	28844284	A	G	0.619879	0.985911	7.20E-07	-0.09691	0.01955
rs62036657	16	28844365	G	A	0.619879	0.985911	7.20E-07	-0.09691	0.01955
rs12444171	16	28845251	G	A	0.619889	0.985864	7.20E-07	-0.09691	0.019551
rs56404918	16	28846840	T	G	0.619892	0.985845	7.20E-07	-0.09691	0.019551
rs55719896	16	28846866	G	A	0.619906	0.985802	7.10E-07	-0.09693	0.019552
rs55830740	16	28847149	G	C	0.61989	0.985867	7.20E-07	-0.0969	0.019551
rs12928404	16	28847246	T	C	0.611397	0.983695	3.40E-07	-0.09932	0.019481
rs12325113	16	28848668	T	C	0.620687	0.985905	4.90E-07	-0.09835	0.019554
rs12325278	16	28848818	A	G	0.61996	0.985587	6.80E-07	-0.0971	0.019554
rs62036658	16	28850371	T	G	0.616072	0.983867	3.00E-07	-0.10005	0.019532
rs3088215	16	28853996	A	C	0.616459	0.999999	7.30E-07	-0.09599	0.019378
rs28403629	16	28854769	A	G	0.619826	0.986143	6.90E-07	-0.09703	0.019548
rs61737565	16	28855522	G	C	0.619826	0.986143	6.90E-07	-0.09703	0.019548
rs4788099	16	28855727	A	G	0.619826	0.986143	6.90E-07	-0.09703	0.019548
rs7187776	16	28857645	A	G	0.616247	0.986017	4.00E-07	-0.09895	0.019524
rs9972693	16	28859802	A	G	0.619858	0.986075	6.40E-07	-0.09733	0.019548
rs113079736	16	28860183	G	A	0.619181	0.985392	8.50E-07	-0.09619	0.01954
rs12325014	16	28860237	C	G	0.619548	0.985883	6.60E-07	-0.09713	0.019538
rs9972768	16	28861734	A	C	0.619858	0.986075	6.40E-07	-0.09733	0.019548
rs56040780	16	28861881	C	T	0.619854	0.986051	6.50E-07	-0.09727	0.019548
rs72793815	16	28863451	G	A	0.619887	0.986105	5.90E-07	-0.09762	0.019547
rs4788100	16	28864673	T	C	0.619885	0.986112	5.90E-07	-0.09761	0.019547
rs62037363	16	28865042	T	C	0.616512	0.999987	6.20E-07	-0.0966	0.019378
rs7205323	16	28865892	C	T	0.619916	0.985995	5.90E-07	-0.09766	0.019548
rs7187333	16	28865916	G	A	0.620767	0.985873	3.90E-07	-0.09921	0.019553
rs4788101	16	28867804	C	T	0.61651	0.999999	6.20E-07	-0.0966	0.019377
rs62037364	16	28868695	G	A	0.615134	0.980701	7.50E-07	-0.09697	0.019593
rs62037365	16	28868962	C	G	0.620334	0.984683	5.60E-07	-0.0979	0.019565
rs11150609	16	28870596	A	G	0.619867	0.986193	5.90E-07	-0.09763	0.019546
rs12446589	16	28870962	G	A	0.620646	0.986127	4.10E-07	-0.09905	0.019551
rs12448902	16	28871191	C	G	0.622659	0.982461	2.00E-07	-0.10195	0.019609
rs62037366	16	28871657	C	A	0.620668	0.986188	4.00E-07	-0.09909	0.01955
rs11859512	16	28871853	C	T	0.619874	0.986165	5.90E-07	-0.09761	0.019547

rs11861132	16	28871860	A	G	0.619867	0.986193	5.90E-07	-0.09763	0.019546
rs11861174	16	28871989	A	G	0.619867	0.986193	5.90E-07	-0.09763	0.019546
rs7499337	16	28872354	G	C	0.620673	0.986196	4.00E-07	-0.09908	0.01955
rs6565229	16	28872598	T	C	0.619861	0.986217	5.90E-07	-0.09764	0.019546
rs4788102	16	28873398	G	A	0.61651	1	6.20E-07	-0.0966	0.019377
rs62037367	16	28874547	C	G	0.619866	0.986195	5.90E-07	-0.09763	0.019547
rs7198606	16	28875122	T	G	0.619861	0.986217	5.90E-07	-0.09764	0.019546
rs11864750	16	28875204	A	T	0.619861	0.986217	5.90E-07	-0.09764	0.019546
rs7193733	16	28875482	A	G	0.619861	0.986217	5.90E-07	-0.09764	0.019546
rs11863370	16	28879484	A	G	0.620023	0.985719	5.70E-07	-0.09782	0.019556
rs8055982	16	28881202	A	C	0.61985	0.986309	5.90E-07	-0.09762	0.019546
rs7498603	16	28882424	T	C	0.619846	0.986305	5.90E-07	-0.09762	0.019546
rs7498665	16	28883241	A	G	0.61985	0.986309	5.90E-07	-0.09762	0.019546
rs62037369	16	28883841	C	T	0.620658	0.986427	4.00E-07	-0.09911	0.01955
rs7359397	16	28885659	C	T	0.617368	0.999966	4.20E-07	-0.09806	0.019384
rs11864107	16	28885931	T	C	0.619877	0.986343	5.80E-07	-0.09771	0.019548
rs3888190	16	28889486	C	A	0.619852	0.98642	5.80E-07	-0.09766	0.019546
rs62037371	16	28890131	C	A	0.619852	0.986418	5.80E-07	-0.09766	0.019546
rs8055138	16	28891465	C	T	0.619853	0.986373	5.90E-07	-0.09764	0.019547
rs11641216	16	28893532	A	G	0.619858	0.986395	5.80E-07	-0.09767	0.019547
rs7498555	16	28893571	T	C	0.620714	0.986399	4.30E-07	-0.09885	0.019549
rs8061590	16	28895130	A	G	0.619783	0.986205	7.10E-07	-0.09692	0.019548

SNP: Single Nucleotide Polymorphism; CHR: Chromosome; A1, Effect allele; A2, Other allele; FREQ: Effect allele frequency; Beta: coefficient from BOLT infinitesimal model; SE: standard error; P: tow-tailed p-value; INFO: Estimated imputation score.

Supplementary Table 21 | G6 associated genome-wide significant SNP list.

SNP	CHR	BP	A1	A2	FREQ	INFO	P	BETA	SE
rs11705789	3	81969196	G	T	0.641119	0.988511	4.50E-08	-0.11126	0.020347
rs10779986	3	81970818	C	G	0.641444	0.987061	4.80E-08	-0.11116	0.02036

SNP: Single Nucleotide Polymorphism; CHR: Chromosome; A1, Effect allele; A2, Other allele; FREQ: Effect allele frequency; Beta: coefficient from BOLT infinitesimal model; SE: standard error; P: two-tailed p-value; INFO: Estimated imputation score.

Supplementary Table 22 | G6 associated SNPs at the suggestive threshold of significance (1 x 10^{-06}).

SNP	CHR	BP	A1	A2	FREQ	INFO	P	BETA	SE
rs7599739	2	27363152	G	T	0.650732	0.991775	5.60E-07	0.103456	0.020678
rs28489942	2	27368588	T	C	0.649187	0.999279	5.50E-07	0.103034	0.020579
rs62128740	2	27369421	C	T	0.650713	0.993176	5.40E-07	0.103589	0.020665
rs1375087	3	81675652	G	C	0.445145	0.980831	8.20E-07	-0.09738	0.019753
rs9852545	3	81677459	T	G	0.445365	0.981274	8.60E-07	-0.0972	0.019754
rs1973805	3	81946539	C	A	0.627484	0.98604	1.20E-07	-0.10705	0.020208
rs9838747	3	81949639	G	C	0.433813	0.979746	2.20E-07	-0.10311	0.01989
rs13093082	3	81949958	C	T	0.656764	0.985314	7.50E-08	-0.11055	0.020557
rs10511101	3	81950302	A	C	0.656779	0.985315	7.60E-08	-0.11053	0.020557
rs9309881	3	81950904	C	T	0.469451	0.987349	4.80E-07	-0.099	0.019663
rs7621968	3	81951460	A	G	0.657004	0.987322	8.10E-08	-0.11016	0.020532
rs1818901	3	81953502	G	A	0.468973	0.98736	4.30E-07	-0.09943	0.019661
rs4479621	3	81954308	T	A	0.468966	0.987362	4.30E-07	-0.09943	0.019661
rs4273393	3	81954355	G	A	0.468855	0.987336	4.50E-07	-0.0992	0.019661
rs11925940	3	81954672	G	C	0.468965	0.987371	4.20E-07	-0.09944	0.019661
rs1851917	3	81955995	C	T	0.468973	0.987309	4.40E-07	-0.09934	0.019662
rs9854004	3	81958242	C	T	0.657375	0.989597	9.70E-08	-0.10934	0.020509
rs9820349	3	81958587	A	G	0.657291	0.989478	8.00E-08	-0.11011	0.020512
rs7653333	3	81960024	C	T	0.469116	0.98996	4.60E-07	-0.09901	0.019632
rs1973804	3	81962522	C	T	0.469977	0.989051	4.60E-07	-0.09902	0.019636
rs957670	3	81962838	C	T	0.467303	0.999495	4.20E-07	-0.09888	0.019539
rs1973803	3	81962978	C	T	0.4701	0.989104	4.40E-07	-0.09921	0.019634
rs6774438	3	81963379	C	T	0.470099	0.9891	4.40E-07	-0.09921	0.019634
rs9810377	3	81963864	G	A	0.498957	0.988112	2.20E-07	-0.10183	0.019649
rs4622918	3	81965382	A	G	0.657986	0.989629	7.50E-08	-0.11038	0.020518
rs4399914	3	81965477	C	T	0.624971	0.999625	5.60E-07	-0.10008	0.020004
rs4479620	3	81965494	G	C	0.628837	0.991233	2.80E-07	-0.10359	0.020156
rs4618242	3	81965535	G	C	0.628786	0.991185	2.70E-07	-0.1037	0.020158
rs2030708	3	81967436	G	A	0.453429	0.987562	1.60E-07	-0.10364	0.019757
rs1971426	3	81968285	C	T	0.453429	0.987562	1.60E-07	-0.10364	0.019757
rs6548779	3	81968512	A	G	0.612208	0.989894	1.60E-07	-0.10492	0.020029
rs1969433	3	81968869	T	C	0.61221	0.989889	1.60E-07	-0.10492	0.020029
rs11705789	3	81969196	G	T	0.641119	0.988511	4.50E-08	-0.11126	0.020347
rs10779986	3	81970818	C	G	0.641444	0.987061	4.80E-08	-0.11116	0.02036
rs10779987	3	81970856	C	T	0.612252	0.988451	1.80E-07	-0.10465	0.020043
rs6808112	3	81971675	A	G	0.453531	0.985821	1.50E-07	-0.10383	0.019767
rs6797240	3	81971856	T	C	0.612477	0.988516	1.70E-07	-0.10473	0.020041

rs6797340	3	81971954	T	G	0.453483	0.985451	1.40E-07	-0.10401	0.019773
rs11127746	3	81974175	C	T	0.641079	0.987262	5.20E-08	-0.11082	0.020361
rs9877408	3	81974558	A	G	0.61241	0.988449	1.80E-07	-0.10467	0.020043
rs1851929	3	81975266	T	A	0.453584	0.985829	1.50E-07	-0.10374	0.019765
rs1851928	3	81975395	C	T	0.612884	0.987509	1.70E-07	-0.10499	0.020057
rs6548780	3	81975649	C	T	0.453702	0.985857	1.40E-07	-0.10395	0.019764
rs13068700	3	81975817	G	C	0.641452	0.987388	5.10E-08	-0.1109	0.020358
rs1851926	3	81977231	A	C	0.612431	0.988544	1.80E-07	-0.10468	0.020042
rs1851925	3	81978614	A	C	0.453668	0.985905	1.40E-07	-0.10401	0.019765
rs9870954	3	81978850	A	G	0.612516	0.988218	1.80E-07	-0.10464	0.020047
rs1851923	3	81979057	G	A	0.45362	0.985888	1.40E-07	-0.10398	0.019764
rs1851919	3	81980402	C	T	0.448709	0.985301	3.60E-07	-0.10088	0.01982
rs1851918	3	81980557	G	A	0.613733	0.987185	4.00E-07	-0.10187	0.020093
rs35779564	3	81982771	G	A	0.630698	0.987019	1.60E-07	-0.10605	0.020248
rs9817070	3	81982936	A	G	0.612413	0.988052	3.90E-07	-0.1018	0.020054
rs9859613	3	81983550	C	T	0.448943	0.985253	3.80E-07	-0.10065	0.019817
rs9309882	3	81984281	G	A	0.448596	0.986235	4.20E-07	-0.10022	0.019812
rs7430321	3	81984643	A	C	0.448587	0.986247	4.20E-07	-0.10023	0.019812
rs9865552	3	81984976	C	T	0.448594	0.986268	4.20E-07	-0.10021	0.019812
rs6778265	3	81985745	T	C	0.612149	0.988388	6.90E-07	-0.09952	0.020044
rs6766011	3	81986136	G	A	0.612162	0.988338	6.90E-07	-0.09953	0.020045
rs7640558	3	81987410	A	G	0.447161	0.986022	3.30E-07	-0.10118	0.019826
rs12636122	3	81989008	G	A	0.447155	0.986008	3.30E-07	-0.10116	0.019825
rs6770740	3	81989081	T	C	0.446544	0.984537	4.10E-07	-0.1005	0.019842
rs6780807	3	81991806	T	G	0.44693	0.986102	3.00E-07	-0.10148	0.019816
rs4324483	3	81992049	C	T	0.446933	0.98611	3.00E-07	-0.10148	0.019816
rs4437168	3	81993039	T	C	0.446006	0.986058	2.20E-07	-0.10261	0.019812
rs4488862	3	81995377	A	G	0.446936	0.986108	3.00E-07	-0.10149	0.019816
rs4296616	3	81995482	C	T	0.446934	0.986114	3.00E-07	-0.10148	0.019816
rs9830303	3	81996166	A	G	0.446023	0.985985	2.20E-07	-0.10272	0.019813
rs4557185	3	81996236	G	A	0.44693	0.9861	3.00E-07	-0.10151	0.019816
rs4482683	3	81996949	A	C	0.446935	0.986117	3.00E-07	-0.10148	0.019816
rs6548786	3	81997510	C	T	0.4468	0.987081	3.00E-07	-0.10151	0.019809
rs6548787	3	81997877	C	T	0.4468	0.98708	3.00E-07	-0.10151	0.019809
rs7620422	3	81999023	C	T	0.446797	0.987074	3.00E-07	-0.10151	0.019809
rs12629007	3	81999266	C	T	0.44831	0.987047	3.40E-07	-0.10092	0.0198
rs4435664	3	81999689	C	T	0.448457	0.987061	4.10E-07	-0.10023	0.019797
rs4261900	3	81999927	A	T	0.446798	0.987068	3.00E-07	-0.1015	0.019809
rs12632474	3	82000232	T	C	0.446798	0.987067	3.00E-07	-0.1015	0.019809

rs13069237	3	82000423	C	T	0.446799	0.987077	3.00E-07	-0.1015	0.019809
rs7626596	3	82000680	G	A	0.446973	0.987416	3.10E-07	-0.10129	0.019798
rs4462992	3	82003569	T	C	0.445717	0.987379	2.50E-07	-0.10216	0.0198
rs4572795	3	82004356	G	A	0.446591	0.987416	3.40E-07	-0.10102	0.019807
rs6548788	3	82005088	T	C	0.445564	0.98606	2.80E-07	-0.10175	0.019816
rs4465962	3	82005704	C	T	0.448204	0.985959	4.90E-07	-0.09968	0.019811
rs4465963	3	82005780	C	T	0.448152	0.985868	5.20E-07	-0.09941	0.019812
rs9816321	3	82006853	T	C	0.445567	0.986063	2.80E-07	-0.10175	0.019816
rs4314192	3	82007411	A	G	0.445563	0.986062	2.80E-07	-0.10175	0.019816
rs4465966	3	82009703	A	G	0.445486	0.985996	2.90E-07	-0.10171	0.019817
rs9309883	3	82014428	C	A	0.445596	0.986199	2.90E-07	-0.1018	0.019837
rs7427000	3	82015818	G	T	0.445295	0.986098	2.60E-07	-0.10215	0.019838
rs9840924	3	82016600	T	C	0.445492	0.986264	3.00E-07	-0.10162	0.019837
rs9275613	6	32684105	C	T	0.889008	0.997205	9.10E-07	-0.15371	0.031299
rs9275614	6	32684257	A	G	0.888754	1	8.40E-07	-0.15385	0.031225
rs9275615	6	32684272	T	A	0.889008	0.997462	8.80E-07	-0.15387	0.031297
rs9275618	6	32684387	A	G	0.889	0.997129	8.50E-07	-0.1541	0.031302
rs3916765	6	32685550	G	A	0.889014	0.999555	5.60E-07	-0.15669	0.031302
rs9276627	6	32743835	C	T	0.918312	0.987555	3.80E-07	-0.18139	0.035725
rs3763355	6	32786882	T	C	0.936491	0.999829	8.40E-08	-0.21428	0.039994
rs4148876	6	32796793	G	A	0.930502	0.987123	4.70E-07	-0.19454	0.038599
rs8092641	18	74253251	C	T	0.421523	0.951494	1.50E-07	-0.10666	0.020313

SNP: Single Nucleotide Polymorphism; CHR: Chromosome; A1, Effect allele; A2, Other allele; FREQ: Effect allele frequency; Beta: coefficient from BOLT infinitesimal model; SE: standard error; P: two-tailed p-value; INFO: Estimated imputation score.

Supplementary Table 23 | Replication of G4 and G6 associated independent SNPs in previously published genome-wide association meta-analysis of human intelligence or general cognitive ability.

PMID	CHR:BP	A1	A2	FREQ	BETA	SE	P	INFO
**G4: rs62034351**								
29942086	16:28565489	G	A	0.648	-0.0258	0.0029	2.935E-19	0.90
28530673	16:28565489	G	A	0.626	-0.0244	0.0063	0.0001	>0.60
**G6: rs11705789**								
29942086	3:81969196	G	T	0.634	-0.0024	0.0028	0.40	0.90
28530673	3:81969196	G	T	0.626	-0.0073	0.0052	0.16	>0.60

Study specific findings were reported with unique identifier number used in PubMed (PMID), genomic coordinates (CHR:BP; GRCh37.p13/ Hg19/); A1: effect allele; A2: other allele; FREQ: effect allele frequency; Beta: coefficient from BOLT infinitesimal model; SE: standard error; P: two-tailed p-value; INFO, estimated imputation score. We harmonised summary statistics for both SNPs manually. We harmonised summary statistics for both SNPs manually. The direction of beta values was reversed as scores for the measure of general cognitive ability or intelligence were calculated using cognitive test scores where higher scores indicated better performances.

Supplementary Table 24 | List of candidate SNP IDs for G4 annotated to have an exonic function.

SNP ID	Gene	Annotation	Exonic Function	Exon	Symbol	Chromosome	Position
16:28354353:G:T	ENSG00000198156	exonic	nonsynonymous SNV	exon7	NPIPB6	16	28354353
16:28506872:A:G	ENSG00000184730	exonic	synonymous SNV	exon2	APOBR	16	28506872
16:28507644:C:G	ENSG00000184730	exonic	nonsynonymous SNV	exon2	APOBR	16	28507644
16:28508048:A:G	ENSG00000184730	exonic	synonymous SNV	exon2	APOBR	16	28508048
16:28508069:C:T	ENSG00000184730	exonic	synonymous SNV	exon2	APOBR	16	28508069
16:28508447:A:G	ENSG00000184730	exonic	synonymous SNV	exon2	APOBR	16	28508447
16:28513403:A:G	ENSG00000197272	exonic	nonsynonymous SNV	exon4	IL27	16	28513403
16:28603655:G:T	ENSG00000197165	exonic	nonsynonymous SNV	exon3	SULT1A2	16	28603655
16:28607228:A:G	ENSG00000197165	exonic	synonymous SNV	exon1	SULT1A2	16	28607228
16:28607232:A:G	ENSG00000197165	exonic	nonsynonymous SNV	exon1	SULT1A2	16	28607232
16:28619911:C:T	ENSG00000196502	exonic	synonymous SNV	exon3	SULT1A1	16	28619911
16:28619920:A:G	ENSG00000196502	exonic	synonymous SNV	exon3	SULT1A1	16	28619920
16:28649787:A:G	ENSG00000255524	exonic	nonsynonymous SNV	exon1	NPIPB8	16	28649787
16:28883241:A:G	ENSG00000178188	exonic	nonsynonymous SNV	exon5	SH2B1	16	28883241

Supplementary Table 25 | Genes prioritised by genome-wide gene-based association analysis, positional, eqtl and chromatin interaction mapping.

SYMBOL	Genes	MAGMA	Pos_Mapped	Eqtl_Mapped	Ci_Mapped
AC009133.15	ENSG00000259952				ENSG00000259952
ALDOA	ENSG00000149925				ENSG00000149925
APOBR	ENSG00000184730	ENSG00000184730	ENSG00000184730	ENSG00000184730	
APOL3	ENSG00000128284			ENSG00000128284	
ASCL2	ENSG00000183734			ENSG00000183734	
ASPHD1	ENSG00000174939				ENSG00000174939
ATP2A1	ENSG00000196296		ENSG00000196296	ENSG00000196296	ENSG00000196296
ATXN2L	ENSG00000168488	ENSG00000168488	ENSG00000168488	ENSG00000168488	ENSG00000168488
BIVM	ENSG00000134897			ENSG00000134897	
BTN3A3	ENSG00000111801			ENSG00000111801	
C14orf132	ENSG00000227051			ENSG00000227051	
C16orf93	ENSG00000196118				ENSG00000196118
C1QB	ENSG00000173369			ENSG00000173369	
C1orf61	ENSG00000125462			ENSG00000125462	
CCDC101	ENSG00000176476	ENSG00000176476	ENSG00000176476	ENSG00000176476	ENSG00000176476
CD19	ENSG00000177455		ENSG00000177455		ENSG00000177455
CD38	ENSG00000004468			ENSG00000004468	
CDC37P1	ENSG00000259982		ENSG00000259982	ENSG00000259982	
CDIPT	ENSG00000103502				ENSG00000103502
CDIPT-AS1	ENSG00000214725				ENSG00000214725
CLN3	ENSG00000188603	ENSG00000188603	ENSG00000188603	ENSG00000188603	
COCH	ENSG00000100473			ENSG00000100473	
CTB-134H23.3	ENSG00000260908			ENSG00000260908	
DAAM1	ENSG00000100592			ENSG00000100592	
DDB2	ENSG00000134574			ENSG00000134574	
DYNLT1	ENSG00000146425			ENSG00000146425	
EIF3C	ENSG00000184110	ENSG00000184110	ENSG00000184110	ENSG00000184110	
EIF3CL	ENSG00000205609	ENSG00000205609	ENSG00000205609	ENSG00000205609	
FAM26F	ENSG00000188820			ENSG00000188820	
FAM57B	ENSG00000149926				ENSG00000149926

GBP1	ENSG00000117228			ENSG00000117228	
GBP1P1	ENSG00000225492			ENSG00000225492	
GBP2	ENSG00000162645			ENSG00000162645	
GCH1	ENSG00000131979			ENSG00000131979	
GPBAR1	ENSG00000179921			ENSG00000179921	
GSDMD	ENSG00000104518			ENSG00000104518	
HAPLN3	ENSG00000140511			ENSG00000140511	
HIRIP3	ENSG00000149929				ENSG00000149929
HKDC1	ENSG00000156510			ENSG00000156510	
IL15	ENSG00000164136			ENSG00000164136	
IL15RA	ENSG00000134470			ENSG00000134470	
IL27	ENSG00000197272	ENSG00000197272	ENSG00000197272	ENSG00000197272	
INO80E	ENSG00000169592				ENSG00000169592
JAK2	ENSG00000096968			ENSG00000096968	
KIF22	ENSG00000079616				ENSG00000079616
LAP3	ENSG00000002549			ENSG00000002549	
LAT	ENSG00000213658			ENSG00000213658	ENSG00000213658
LGALS3BP	ENSG00000108679			ENSG00000108679	
LINC00324	ENSG00000178977			ENSG00000178977	
MAF	ENSG00000178573			ENSG00000178573	
MAZ	ENSG00000103495				ENSG00000103495
MIR4721	ENSG00000264455		ENSG00000264455	ENSG00000264455	
MOV10	ENSG00000155363			ENSG00000155363	
MT2A	ENSG00000125148			ENSG00000125148	
MUC1	ENSG00000185499			ENSG00000185499	
MYLPF	ENSG00000180209				ENSG00000180209
MYOF	ENSG00000138119			ENSG00000138119	
N4BP2L1	ENSG00000139597			ENSG00000139597	
NFATC2IP	ENSG00000176953		ENSG00000176953	ENSG00000176953	ENSG00000176953
NLRC5	ENSG00000140853			ENSG00000140853	
NPIPB6	ENSG00000198156	ENSG00000198156	ENSG00000198156	ENSG00000198156	
NPIPB7	ENSG00000233232	ENSG00000233232	ENSG00000233232	ENSG00000233232	
NPIPB8	ENSG00000255524	ENSG00000255524	ENSG00000255524	ENSG00000255524	
NPIPB9	ENSG00000196993	ENSG00000196993	ENSG00000196993	ENSG00000196993	
NUPR1	ENSG00000176046	ENSG00000176046	ENSG00000176046	ENSG00000176046	ENSG00000176046

ORC3	ENSG00000135336		ENSG00000135336	
PARP3	ENSG00000041880		ENSG00000041880	
PATL2	ENSG00000229474		ENSG00000229474	
PBX3	ENSG00000167081		ENSG00000167081	
PHKG2	ENSG00000156873			ENSG00000156873
PSMB10	ENSG00000205220		ENSG00000205220	
PSME1	ENSG00000092010		ENSG00000092010	
PSME2	ENSG00000100911		ENSG00000100911	
RABEP2	ENSG00000177548	ENSG00000177548	ENSG00000177548	ENSG00000177548
RARRES3	ENSG00000133321		ENSG00000133321	
RGS1	ENSG00000090104		ENSG00000090104	
RNF31	ENSG00000092098		ENSG00000092098	
RNF40	ENSG00000103549			ENSG00000103549
RP11-1348G14.1	ENSG00000240634	ENSG00000240634		
RP11-1348G14.4	ENSG00000251417	ENSG00000251417	ENSG00000251417	
RP11-1348G14.5	ENSG00000260796	ENSG00000260796	ENSG00000260796	ENSG00000260796
RP11-1348G14.6	ENSG00000270424	ENSG00000270424	ENSG00000270424	
RP11-22P6.2	ENSG00000261766	ENSG00000261766	ENSG00000261766	ENSG00000261766
RP11-22P6.3	ENSG00000260442	ENSG00000260442	ENSG00000260442	ENSG00000260442
RP11-231C14.4	ENSG00000169203		ENSG00000169203	
RP11-24N18.1	ENSG00000260570	ENSG00000260570	ENSG00000260570	
RP11-264B17.2	ENSG00000260853		ENSG00000260853	
RP11-264B17.3	ENSG00000261067		ENSG00000261067	ENSG00000261067
RP11-264B17.4	ENSG00000260367		ENSG00000260367	ENSG00000260367
RP11-426C22.5	ENSG00000260517		ENSG00000260517	
RP11-435I10.3	ENSG00000261089	ENSG00000261089	ENSG00000261089	
RP11-435I10.5	ENSG00000271623	ENSG00000271623	ENSG00000271623	
RP11-57A19.2	ENSG00000246465		ENSG00000246465	ENSG00000246465
RP11-57A19.4	ENSG00000261419	ENSG00000261419		
RP11-666O2.1	ENSG00000271495	ENSG00000271495		
RP11-693N9.2	ENSG00000235505		ENSG00000235505	
RPS6KA5	ENSG00000100784		ENSG00000100784	
RRN3P2	ENSG00000103472		ENSG00000103472	
SBK1	ENSG00000188322	ENSG00000188322	ENSG00000188322	ENSG00000188322
SDSL	ENSG00000139410		ENSG00000139410	

SEPHS2	ENSG00000179918				ENSG00000179918
SEPTIN1	ENSG00000180096				ENSG00000180096
SEZ6L2	ENSG00000174938				ENSG00000174938
SH2B1	ENSG00000178188	ENSG00000178188	ENSG00000178188	ENSG00000178188	
SMCO4	ENSG00000166002			ENSG00000166002	
SNORA43	ENSG00000252461		ENSG00000252461		ENSG00000252461
SNX10	ENSG00000086300			ENSG00000086300	
SPNS1	ENSG00000169682			ENSG00000169682	ENSG00000169682
STAT1	ENSG00000115415			ENSG00000115415	
STAT5A	ENSG00000126561			ENSG00000126561	
SULT1A1	ENSG00000196502	ENSG00000196502	ENSG00000196502	ENSG00000196502	ENSG00000196502
SULT1A2	ENSG00000197165	ENSG00000197165	ENSG00000197165	ENSG00000197165	
SULT1A4	ENSG00000213648			ENSG00000213648	
SYNE2	ENSG00000054654			ENSG00000054654	
TAOK2	ENSG00000149930				ENSG00000149930
TBC1D10B	ENSG00000169221				ENSG00000169221
TTC32	ENSG00000183891			ENSG00000183891	
TUFM	ENSG00000178952	ENSG00000178952	ENSG00000178952	ENSG00000178952	
UBE2L6	ENSG00000156587			ENSG00000156587	
VAMP5	ENSG00000168899			ENSG00000168899	
VCPIP1	ENSG00000175073			ENSG00000175073	
WARS	ENSG00000140105			ENSG00000140105	
WNT7A	ENSG00000154764			ENSG00000154764	
XPO6	ENSG00000169180			ENSG00000169180	ENSG00000169180
ZNF629	ENSG00000102870				ENSG00000102870
ZNF771	ENSG00000179965				ENSG00000179965
ZNF800	ENSG00000048405			ENSG00000048405	
snoU13	ENSG00000238684		ENSG00000238684		

Pos_mapping column presents genes prioritised by positional mapping strategy.
Eqtl_mapping column presents genes prioritised by eQTL mapping strategy.
Ci_mapping column presents genes prioritised by chromatin interaction mapping strategy.

Supplementary Table 26 | Top 10 clusters with their representative enriched terms for G4. "Log10(P)" is the p-value (computed using the cumulative hypergeometric distribution) in log base 10. "Log10(q)" is the multi-test adjusted p-value in log base 10.

Category	Term	Description	LogP	Log (q-value)	Symbols
Immunologic	M3408	GSE1432 CTRL VS IFNG 24H MICROGLIA DN	-29.7606	-25.1823	ASCL2,CD38,GBP1,GBP2,GCH1,IL15,IL15RA,JAK2, LGALS3BP,PSMB10,PSME1,PSME2,PLAAT4,STAT1, DYNLT1,WARS1,UBE2L6,BTN3A3,VAMP5,MYOF, SNX10,LAP3,SMCO4,APOL3,KIF22,STAT5A,RNF31, GSDMD,C1QB,DDB2,MUC1,N4BP2L1,PHKG2, SEPHS2,XPO6,MT2A,NLRC5,PARP3,SYNE2,RPS6KA5, GPBAR1,CDIPT,HAPLN3,PATL2,CALHM6,NUPR1, LINC00324,MOV10,APOBR,RGS1,SEZ6L2,SBK1, ORC3,MAF,SNORA17B,GBP1P1,SDSL
WikiPathways	WP4950	16p11.2 distal deletion syndrome	-19.0769	-15.3438	ATP2A1,CD19,JAK2,TUFM,ATXN2L,SH2B1,LAT, RABEP2,SPNS1,NFATC2IP,MIR4721
Immunologic	M9306	GSE40685 TREG VS FOXP3 KO TREG PRECURSOR UP	-18.4715	-14.8972	ASCL2,GBP2,GCH1,JAK2,MOV10,PSME1,PSME2, PLAAT4,STAT1,UBE2L6,BTN3A3,VAMP5,SNX10, SMCO4,NLRC5,HAPLN3,CALHM6
Immunologic	M9308	GSE40685 TREG VS FOXP3 KO TREG PRECURSOR DN	-14.0704	-10.8933	C1QB,CD38,GCH1,IL15,JAK2,LGALS3BP,PSME1, PLAAT4,STAT1,SNX10,LAP3,APOL3,NLRC5, HAPLN3,IL15RA,MOV10,MT2A,PSME2,DYNLT1, UBE2L6,GBP1,GBP2,BTN3A3,CALHM6,PATL2
Immunologic	M7320	GSE19888 ADENOSINE A3R INH PRETREAT AND ACT BY A3R VS TCELL MEMBRANES ACT MAST CELL UP	-14.0401	-10.8933	GBP2,GCH1,IL15,PSMB10,PSME1,PSME2,STAT1, WARS1,UBE2L6,PARP3,SNX10,RNF31,GSDMD, CALHM6,IL15RA,LGALS3BP,MOV10,NUPR1, NLRC5,LAP3,C1QB,JAK2,CD38,RGS1,BTN3A3, MT2A,SYNE2,MYOF,GBP1,ALDOA,APOL3, N4BP2L1,XPO6,COCH,STAT5A,MUC1,LAT,INO80E
WikiPathways	WP4949	16p11.2 proximal deletion syndrome	-12.9307	-9.84381	ALDOA,KIF22,MAZ,HIRIP3,TAOK2,CDIPT, SEZ6L2,TLCD3B,ASPHD1,INO80E,PHKG2, UBE2L6,SEPHS2
Immunologic	M9797	GSE42724 NAIVE BCELL VS PLASMABLAST UP	-12.7313	-9.65822	CD38,GBP1,GCH1,IL15RA,LGALS3BP,MOV10, PSME1,PLAAT4,STAT1,UBE2L6,XPO6,LAP3, NLRC5,DYNLT1,WARS1,IL15,N4BP2L1, RPS6KA5,SBK1,MT2A,VAMP5,SEZ6L2, BTN3A3,PSME2,C1QB
Immunologic	M7543	GSE21546 WT VS SAP1A KO DP THYMOCYTES UP	-11.3962	-8.37428	GBP1,LGALS3BP,MOV10,MT2A,PSME1,PSME2, STAT1,UBE2L6,BTN3A3,VAMP5,LAP3,NLRC5, TTC32,PATL2,MYOF,EIF3C,SH2B1,DAAM1, SEZ6L2,SEPHS2
Reactome	R-HSA-1280215	Cytokine Signaling in Immune system	-10.2207	-7.2957	GBP1,GBP2,IL15,IL15RA,JAK2,MT2A,MUC1, PSMB10,PSME1,PSME2,STAT1,STAT5A, UBE2L6,RPS6KA5,SH2B1,GSDMD,NLRC5, IL27,PLAAT4,LAP3,ZNF800,LAT,SBK1,DAAM1,

Immunologic	M6843	GSE7548 DAY7 VS DAY28 PCC IMMUNIZATION CD4 TCELL DN	-10.0034	-7.14947	SNX10,VCPIP1,MAF,RGS1,DDB2,WNT7A,<br>RNF31,NUPR1,LGALS3BP<br>ASCL2,DDB2,LGALS3BP,PSMB10,PSME1,<br>PSME2,PLAAT4,WARS1,RPS6KA5,BTN3A3,<br>VAMP5,GBP2,PHKG2,C1QB,CD38,STAT1,<br>STAT5A,XPO6,N4BP2L1,COCH,MT2A,DYNLT1,<br>MYOF,SMCO4,IL15,BIVM

Supplementary Table 27 | Estimated genetic correlation for G4 and G6 with adulthood IQ, childhood IQ, educational attainment, and Alzheimer's Disease.

		G4		G6	
Phenotypes	PMID [a]	r_g (se) [a, *]	P	r_g (se) [a, *]	P
Adulthood IQ	29942086	0.88 (0.07)	1.27e-37	0.74 (0.14)	2.13e-07
Adulthood IQ	28530673	0.88 (0.11)	3.76e-15	0.75 (0.22)	0.0006
Childhood IQ	23358156	0.84 (0.14)	2.35e-09	0.68 (0.24)	0.0046
Educational Attainment	30038396	0.71 (0.06)	1.9e-37	0.29 (0.07)	9.4e-05
Alzheimer's Disease	24162737	0.48 (0.15)	0.001	0.37 (0.23)	0.10

[a]PMID: PubMed Identifier; r_g: estimate of genetic correlation; se: Standard Error; P: two-tailed p-value.
*Positive correlations indicate worse performance of trait is positively associated with worse performance on G4 and G6.

Supplementary Table 28 | Stimuli for the vocabulary test.

1. Quarrel	2. Adhere	3. Handle	4. Sieve	5. Meander
6. Obsolete	7. Revoked	8. Ambiguous	9. Assail	10. Obsequious
11. Puerile	12. Paroxysm	13. Ubiquitous	14. Pinion	15. Audaciously
16. Putative	17. Kaolin	18. Apocryphal	19. Arcane	20. Epigone

Reference

95. Machiela, M.J. & Chanock, S.J. LDlink: a web-based application for exploring population-specific haplotype structure and linking correlated alleles of possible functional variants. *Bioinformatics* **31**, 3555-3557 (2015).
