# Supplementary material for: Dynamics of cognitive variability with age and its genetic underpinning in NIHR BioResource Genes and Cognition cohort participants
Source: Nat Med. 2024 May 14;30(6):1739–48. doi: 10.1038/s41591-024-02960-5 (PMC11186791; doi:10.1038/s41591-024-02960-5)
Supplement: Supplementary file 2 — Reporting Summary [file 41591_2024_2960_MOESM2_ESM.pdf]

Corresponding author(s): Brian D.M. Tom and Patrick F. Chinnery

Last updated by author(s): Jan 29, 2024

Reporting Summary

Nature Portfolio wishes to improve the reproducibility of the work that we publish. This form provides structure for consistency and transparency in reporting. For further information on Nature Portfolio policies, see our Editorial Policies and the Editorial Policy Checklist.

Statistics

For all statistical analyses, confirm that the following items are present in the figure legend, table legend, main text, or Methods section.

n/a	Confirmed	
☐	☒	The exact sample size (*n*) for each experimental group/condition, given as a discrete number and unit of measurement
☐	☒	A statement on whether measurements were taken from distinct samples or whether the same sample was measured repeatedly
☐	☒	The statistical test(s) used AND whether they are one- or two-sided<br>*Only common tests should be described solely by name; describe more complex techniques in the Methods section.*
☐	☒	A description of all covariates tested
☐	☒	A description of any assumptions or corrections, such as tests of normality and adjustment for multiple comparisons
☐	☒	A full description of the statistical parameters including central tendency (e.g. means) or other basic estimates (e.g. regression coefficient) AND variation (e.g. standard deviation) or associated estimates of uncertainty (e.g. confidence intervals)
☐	☒	For null hypothesis testing, the test statistic (e.g. *F*, *t*, *r*) with confidence intervals, effect sizes, degrees of freedom and *P* value noted<br>*Give P values as exact values whenever suitable.*
☒	☐	For Bayesian analysis, information on the choice of priors and Markov chain Monte Carlo settings
☒	☐	For hierarchical and complex designs, identification of the appropriate level for tests and full reporting of outcomes
☐	☒	Estimates of effect sizes (e.g. Cohen's *d*, Pearson's *r*), indicating how they were calculated

Our web collection on statistics for biologists contains articles on many of the points above.

Software and code

Policy information about availability of computer code

Data collection	Cognitive data was collected using "Cognitive Test (v4.4.7-v.5.6.7)" application and a questionnaire asking for basic lifestyle and health-related information
Data analysis	Analyses were undertaken using R and various standard publicly available statistical genetics software packages. Any tools used for data analysis are reported clearly in the paper and referenced. The packages we used included: R (v4.2.0 and v4.2.2); BOLT-LMM (V.2.3.6); BOLT-REML (v.2.4); PRSice-2 (v2.3.3); GCTA (v1.94.1); LDSR (v1.0.1); FUMA (v1.5.2); Metascape 3.5; CTG-VL (https://vl.genoma.io/); COLOC (v5); FINEMAP (v1.4.2), LDmatrix tool (https://ldlink.nih.gov/?tab=ldmatrix), and GTEx portal (https://www.gtexportal.org/). The codes used for cognitive data cleaning are available on GitHub (https://github.com/shafiqnoa/Genes-and-Cognition-Phase-1/tree/main/Phase1_Cognitive_Data_Clean).

For manuscripts utilizing custom algorithms or software that are central to the research but not yet described in published literature, software must be made available to editors and reviewers. We strongly encourage code deposition in a community repository (e.g. GitHub). See the Nature Portfolio guidelines for submitting code & software for further information.

Data

Policy information about availability of data

All manuscripts must include a data availability statement. This statement should provide the following information, where applicable:

- Accession codes, unique identifiers, or web links for publicly available datasets
- A description of any restrictions on data availability
- For clinical datasets or third party data, please ensure that the statement adheres to our policy

Summary statistics for G4 and G6 GWAS were deposited at Zenodo (10.5281/zenodo.10836380). Other data relevant to the study are included in the article or uploaded as online supplementary information. NIHR Bioresource holds individual-level genetic and phenotypic data for genes and cognitive study participants which can be accessed through https://bioresource.nihr.ac.uk/using-our-bioresource/.

Human research participants

Policy information about studies involving human research participants and Sex and Gender in Research.

Reporting on sex and gender	We used the term 'Gender' when the data was self-reported and 'Sex' when genetically inferred.
Population characteristics	The baseline characteristics of participants are presented in Table 1.
Recruitment	Volunteers were recruited from NIHR BioResource, which recruits participants from the general population and National Health Service organizations in England.
Ethics oversight	The NIHR BioResource operates under two separate set of ethics: a Study for the recruitment of Rare Disease (RD) patients (REC REF: 13/EE/0325) and a Research Tissue Bank (RTB) for the recruitment of all other participants (REC REF: 17/EE/0025). Ethical approval for the G&C study was obtained from the North of Scotland Research Ethics Committee (REC REF: 19/NS/0118).

Note that full information on the approval of the study protocol must also be provided in the manuscript.

Field-specific reporting

Please select the one below that is the best fit for your research. If you are not sure, read the appropriate sections before making your selection.

☒ Life sciences ☐ Behavioural & social sciences ☐ Ecological, evolutionary & environmental sciences

For a reference copy of the document with all sections, see nature.com/documents/nr-reporting-summary-flat.pdf

Life sciences study design

All studies must disclose on these points even when the disclosure is negative.

Sample size	No sample size calculation was required for this study. We utilized all the data from recruited volunteers that were available after the initial data cleaning process. The total sample size used in this study was 21,051. Depending on the analysis presented, the sample size varied from 9,536 to 21,051.
Data exclusions	A small number of individuals (n=123 out of 21,051) were excluded because they had a medical disorder or disability which could bias the effect estimates.
Replication	To validate GWAS findings, we reviewed two previous meta-analyses of intelligence (N=78,308-269,867). The G4-associated locus was associated in both meta-analyses. However, the G6-associated locus was not associated in either meta-analysis.
Randomization	The study was observational.
Blinding	This was an observational study, so no blinding was employed.

Reporting for specific materials, systems and methods

We require information from authors about some types of materials, experimental systems and methods used in many studies. Here, indicate whether each material, system or method listed is relevant to your study. If you are not sure if a list item applies to your research, read the appropriate section before selecting a response.

Materials & experimental systems

n/a	Involved in the study	
☒	☐	Antibodies
☒	☐	Eukaryotic cell lines
☒	☐	Palaeontology and archaeology
☒	☐	Animals and other organisms
☒	☐	Clinical data
☒	☐	Dual use research of concern

Methods

n/a	Involved in the study	
☒	☐	ChIP-seq
☒	☐	Flow cytometry
☒	☐	MRI-based neuroimaging
